# Structure–Activity Relationship Studies on Oxazolo[3,4-*a*]pyrazine Derivatives Leading to the Discovery of a Novel Neuropeptide S Receptor Antagonist with Potent *In Vivo* Activity

**DOI:** 10.1021/acs.jmedchem.0c02223

**Published:** 2021-03-18

**Authors:** Valentina Albanese, Chiara Ruzza, Erika Marzola, Tatiana Bernardi, Martina Fabbri, Anna Fantinati, Claudio Trapella, Rainer K. Reinscheid, Federica Ferrari, Chiara Sturaro, Girolamo Calò, Giorgio Amendola, Sandro Cosconati, Salvatore Pacifico, Remo Guerrini, Delia Preti

**Affiliations:** † Department of Chemical, Pharmaceutical and Agricultural Sciences, University of Ferrara, Via Luigi Borsari 46, 44121 Ferrara, Italy; ‡ Department of Neuroscience and Rehabilitation, Section of Pharmacology, University of Ferrara, Via Fossato di Mortara 17/19, 44121 Ferrara, Italy; § LTTA Laboratory for Advanced Therapies, Technopole of Ferrara, 44121 Ferrara, Italy; ∥ Institute of Pharmacology and Toxicology, Jena University Hospital, Friedrich Schiller University, 07747 Jena, Germany; ⊥ Institute of Physiology I, University Hospital Münster, University of Münster, 48149 Münster, Germany; # Department of Pharmaceutical and Pharmacological Sciences, University of Padova, Largo Meneghetti, 2, 35131 Padova, Italy; ¶ “DiSTABiF”, Università della Campania “Luigi Vanvitelli”, Via Vivaldi 43, 81100 Caserta, Italy

## Abstract

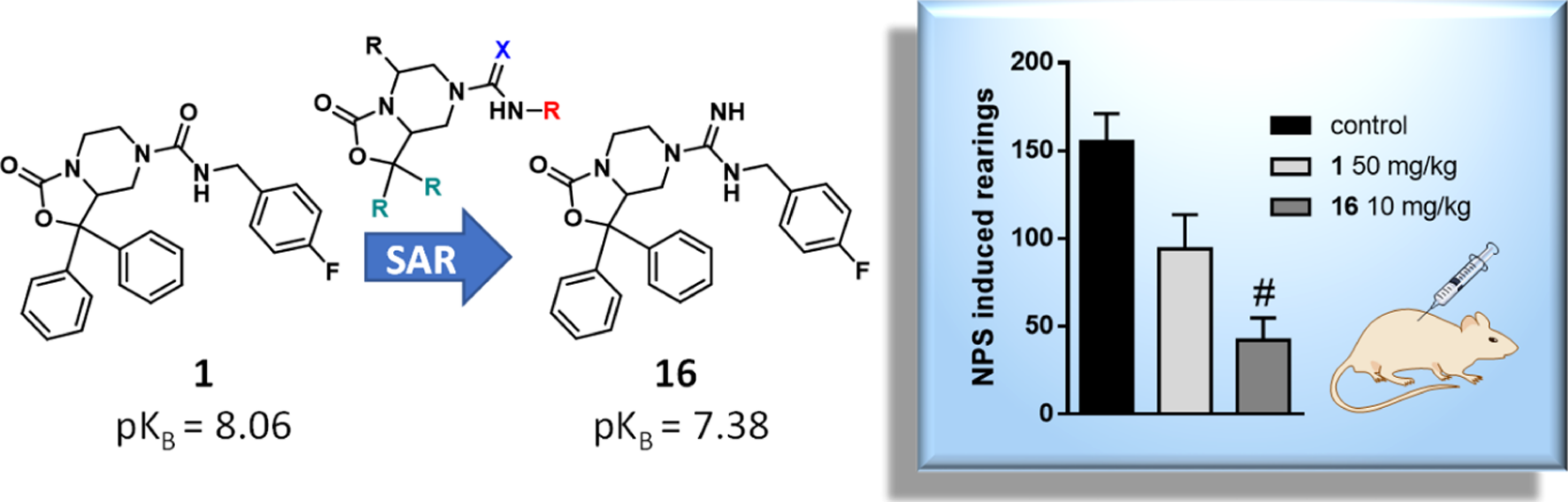

Neuropeptide S modulates important neurobiological functions including locomotion, anxiety, and drug abuse through interaction with its G protein-coupled receptor known as neuropeptide S receptor (NPSR). NPSR antagonists are potentially useful for the treatment of substance abuse disorders against which there is an urgent need for new effective therapeutic approaches. Potent NPSR antagonists *in vitro* have been discovered which, however, require further optimization of their *in vivo* pharmacological profile. This work describes a new series of NPSR antagonists of the oxazolo[3,4-*a*]pyrazine class. The guanidine derivative **16** exhibited nanomolar activity *in vitro* and 5-fold improved potency *in vivo* compared to **SHA-68**, a reference pharmacological tool in this field. Compound **16** can be considered a new tool for research studies on the translational potential of the NPSergic system. An in-depth molecular modeling investigation was also performed to gain new insights into the observed structure–activity relationships and provide an updated model of ligand/NPSR interactions.

## Introduction

Neuropeptide S (NPS), identified in 2002 by a reverse pharmacology approach,^1^ is the endogenous ligand of a previous orphan G protein-coupled receptor (GPCR), now named neuropeptide S receptor (NPSR). NPS is a 20 amino acid neuropeptide (primary sequence in humans: SFRNGVGTGMKKTSFQRAKS) highly conserved among different species, and it owes its name to the serine residue at the 1-position of the peptide sequence. NPSR shows a moderate homology with the other members of the GPCR family. The *in vitro* pharmacology of the human and mouse NPSR showed that NPS increases both intracellular calcium levels and cAMP accumulation with EC_50_ values in the low nanomolar range. This indicates that NPSR can signal via both Gq and Gs pathways to increase cellular excitability.^2,3^ In the rodent brain, NPS is expressed only in few neurons in the peri-locus coeruleus region. On the contrary, NPSR is widely expressed in several brain regions (i.e., hypothalamus, endopiriform nucleus, amygdala, subiculum, cortex, and nuclei of the thalamic midline).^4,5^*In vivo*, NPS has been shown to control several biological functions in rodents including stress, anxiety, social behavior, locomotor activity, wakefulness, food intake and gastrointestinal functions, memory processes, pain, and drug abuse.^6,7^ As far as the therapeutic potential of selective NPSR ligands is concerned, NPSR agonists may be useful as innovative anxiolytics devoid of sedative effects, analgesics, and nootropics. On the other hand, NPSR antagonists may be useful to treat substance abuse disorders against which there is an urgent need for the exploration of novel potential drug targets and for developing innovative therapeutic approaches.^8^

**Figure 1:**
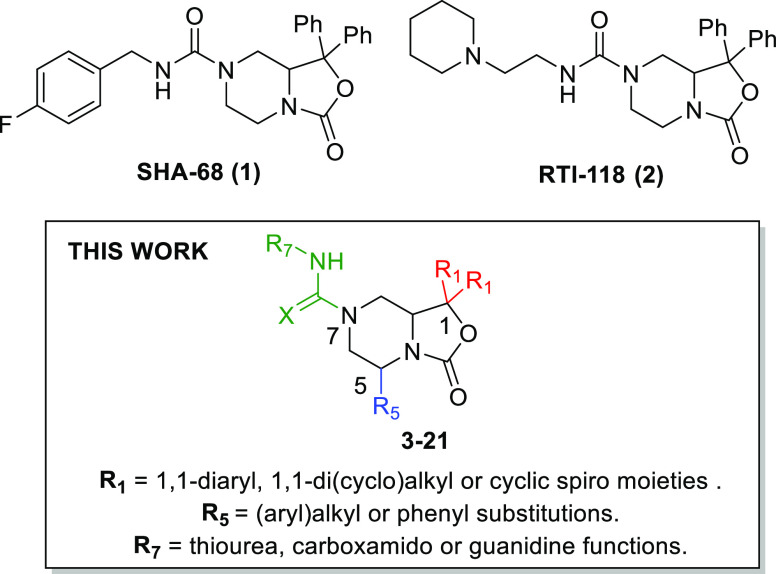
SAR extension performed in this work around the oxazolo[3,4-*a*]pyrazine nucleus of known NPSR antagonists **1** and **2**.

NPSR antagonists with potent *in vitro* activity have been developed in the last few years and a few compounds are currently in use as pharmacological tools.^7^ Among these, oxazolo[3,4-*a*]pyrazine derivatives have been first reported in 2005 by Takeda Pharmaceuticals,^9^ and **SHA-68** (**1**, Figure 1) is the most representative member of this class.^10^ Compound **1** was shown to display nanomolar antagonist potency values (p*A*_2_/p*K*_B_) ranging from 7.28 to 8.16 toward the hNPSR-Asn^107^ variant and from 7.55 to 8.03 toward the hNPSR-Ile^107^ variant. Also, compound **1** exhibited high affinity for the hNPSR in radioligand-binding experiments (p*K*_i_ = 7.32) and high selectivity over several unrelated GPCRs.^7^

The *in vivo* pharmacological profile of **1** has been explored in various animal models in which considerably variable effectiveness was observed according to different assays, which has been interpreted as due to suboptimal pharmacokinetic properties of the molecule.^10−15^ As a first attempt to overcome these limits, Hassler et al. developed the piperidine derivative **RTI-118** (**2**, Figure 1) that exhibited lower potency (hNPSR-Asn^107^ p*A*_2_ = 6.31; hNPSR-Ile^107^ Ca^2+^ pA_2_ = 6.96) *in vitro*(16) but a slightly improved *in vivo* effectiveness in reducing cocaine self-administration and seeking behavior in rats; these results were ascribed to the higher water solubility of the molecule.^17^ Nonetheless, there is a generally recognized need for further optimizing the pharmacological profile and, above all, the drug-likeness properties of oxazolo[3,4-*a*]pyrazine ligands to obtain even more potent NPSR antagonist tools to be employed *in vivo* in preclinical studies. These ligands could be extremely useful for understanding the real therapeutic potential of the NPSergic system. This prompted us to extend the structure–activity relationship studies in this field investigating new and unexplored modifications of the bicyclic piperazine nucleus of compounds **1** and **2**. Thus, in this work, we describe the synthesis and the *in vitro* and *in vivo* biological evaluation of oxazolo[3,4-*a*]pyrazine derivatives resulting from a series of substitutions at the 1-, 5-, and 7-positions, as summarized in Figure 1. Moreover, molecular modeling studies were performed to gain new insights into the structure–activity relationships observed for the newly discovered NPSR ligands and provide an updated atomistic model of ligand/NPSR interactions.

## Results and Discussion

### Chemistry

**Scheme 1:**
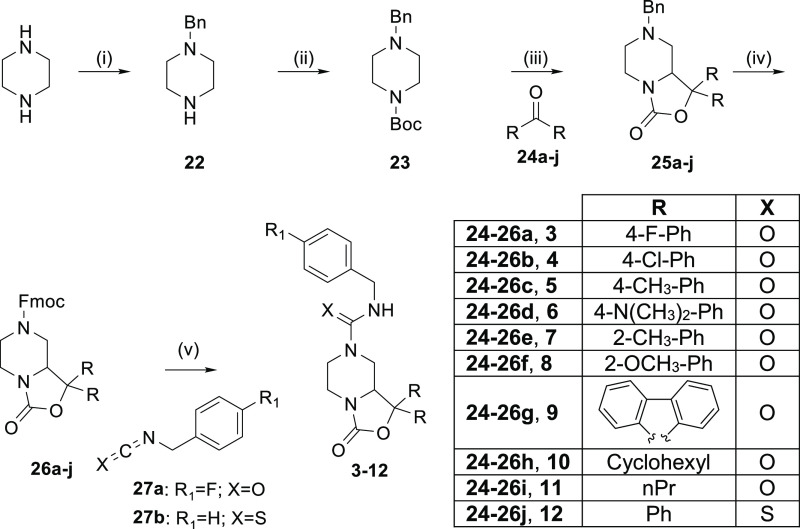
Synthesis of Final Compounds **3–12** Reagents and conditions: (i) BnBr, EtOH, 75 °C, 5 h; (ii) Boc_2_O, DMAP, tetraethylammonium, CH_2_Cl_2_, rt, 0.5 h; (iii) *sec*-BuLi, TMEDA, THF, −78 °C, 6 h; (iv) FmocCl, MeCN, 90 °C, 5 h; (v) DBU, THF, rt, 2 h.

As depicted in Scheme 1, *N*-Fmoc-oxazolo-piperazines with a general structure **26** were employed as synthetic precursors to obtain the final compounds **3–12** in analogy with the approach previously applied for the synthesis of **1** by Okamura et al.^10^ Specifically, intermediates **26** were obtained starting from unsubstituted piperazine that was first monoalkylated with benzyl-bromide and next Boc-protected on the second piperazine nitrogen to give compound **23**. Subsequently, the desired *N*-benzyl-protected oxazolo[3,4-*a*]pyrazines **25a–j** were obtained through an ortho-lithiation reaction, in the presence of *sec*-butyllithium (*sec*-BuLi) as the base and various symmetric aromatic/aliphatic ketones (**24a–j**) as electrophiles. The benzyl function was next replaced with an Fmoc-group by treatment with FmocCl and finally, in order to achieve compounds **3–11**, **26a–i** were reacted with 4-F-benzyl-isocyanate, while compound **12** was obtained from **26j** by treatment with benzyl isothiocyanate.

**Scheme 2:**
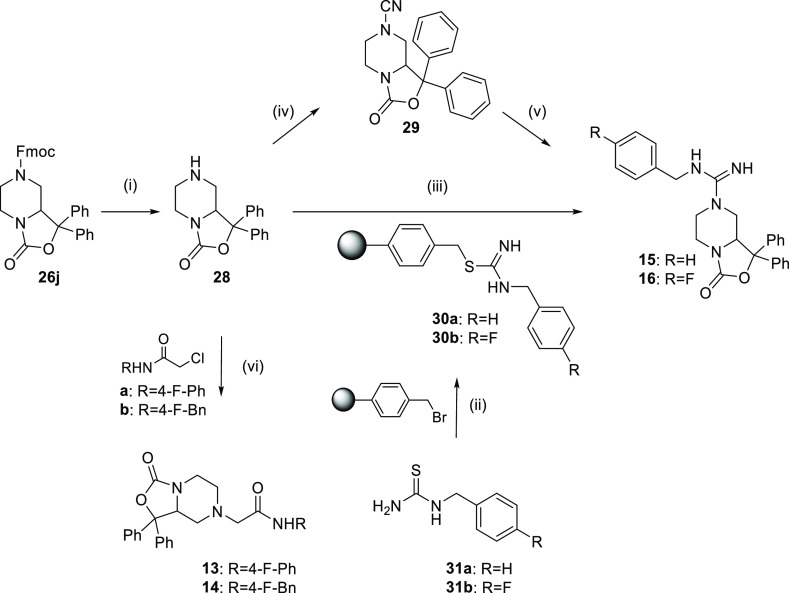
Synthesis of Final Compounds **13–16** Reagents and conditions: (i) DBU, THF, rt, 2 h; (ii) CH_2_Cl_2_–DMF (2:1), 50 °C, 4 h; (iii) HgCl_2_, CH_3_CN, 90 °C, 24 h; (iv) BrCN,CH_2_Cl_2_, NaHCO_3_, H_2_O, 30 min at 0 °C, 24 h rt; (v) benzyl amine or 4-fluoro benzylamine, *p*-TsOH, DMSO, 60 °C, 18 h; (vi) K_2_CO_3_, CH_3_CN, 90 °C, 4 h.

The amide derivatives **13–14** and the guanidine analogues **15–16** were synthesized according to Scheme 2 starting from **26j** that was first deprotected by treatment with 1,8-diazabicyclo[5.4.0]undec-7-ene (DBU). The *N*-alkylation of **28** with 2-chloro-*N*-(4-fluorophenyl)acetamide or 2-chloro-*N*-(4-fluorobenzyl)acetamide produced the final compounds **13** and **14**, respectively. In order to obtain the guanidine derivatives **15** and **16**, we explored two different synthetic strategies. The first approach, developed in the liquid phase, involved the reaction of **28** with cyanogen bromide, giving the key intermediate **29**. Then, the addition of benzylamine or 4-fluorobenzylamine in the presence of *p*-toluenesulfonic acid (*p*-TsOH) provided the desired final products.

The second pathway resulted from the optimization of a known solid-phase approach.^18^ In this case, a bromomethyl polymeric resin was functionalized with 1-(4-fluorobenzyl)thiourea or 1-(benzyl)thiourea affording **30a–b**. Subsequently, the loaded resin was reacted with **28** in the presence of HgCl_2_ to produce the desired guanidine derivatives in good yields.

**Scheme 3:**
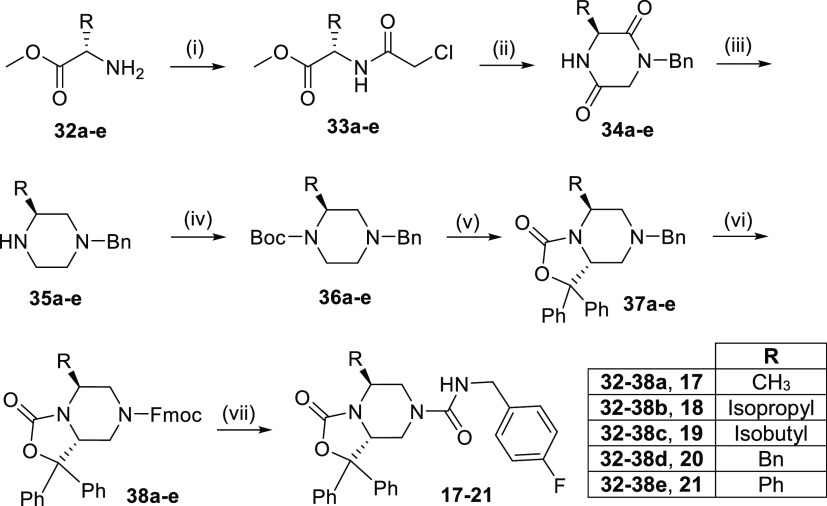
Synthesis of Final Compounds **17–21** Reagents and conditions: (i) NaHCO_3_, chloroacetyl chloride, toluene, 0 °C to rt, overnight; (ii) Et_3_N, benzylamine, dioxane, reflux, 20 h; (iii) LiAlH_4_, THF, reflux, 3 h; (iv) Boc_2_O, THF, 0 °C to rt, 1 h; (v) benzophenone, *sec*-BuLi, TMEDA, THF, −78 °C, 3 h; (vi) FmocCl, MeCN, 90 °C, 5 h and then rt, 18 h; (vii) 4-fluorobenzyl isocyanate, DBU, THF, rt, 2 h.

Finally, compounds **17–21** were prepared as depicted in Scheme 3 starting from five different commercially available l-amino acid methyl esters (**32a–e**). Specifically, the piperazine-diones **34a–e** were obtained in two simple steps involving a first amino acid acylation with chloroacetyl chloride, followed by cyclization with benzylamine.^19^ The subsequent reduction with LiAlH_4_ gave **35a–e** that were then protected with di-*tert*-butyl dicarbonate (Boc_2_O).^20^ The resulting orthogonally protected piperazines **36a–e** were employed in the ortho-lithiation reaction to give the benzyl derivatives **37a–e**, followed by treatment with FmocCl and the final addition reaction with 4-fluorobenzyl isocyanate as described above.

The ortho-lithiation step is of key importance for the stereochemical course of the synthetic approach leading to the final compounds **17–21**. This reaction takes advantage of the defined stereochemistry at C-2 of intermediates **36** that is imposed by the choice of the starting amino acid as demonstrated in the literature for analogous piperazine systems obtained through the same strategy.^21^ The spatial orientation of the substituents around the asymmetric C-8*a*, generated in the bicyclic derivatives **37** during the ortho-lithiation reaction, was driven by the absolute configuration previously introduced at C-2. A single diastereoisomer was isolated in all cases in which an antirelative stereochemistry between the substituent at the C-5 and the oxazole ring fused at C-8a was expected according to previous studies^21^ and as confirmed by NOE spectroscopy performed on the reference compound **17** (Figures S1 and S2). In this experiment, the irradiation of the methyl protons at the 5-position produced an important enhancement of the signal of the proton at the C-8*a* position which is in accordance with a syn relationship. Thus, the absolute (5*S*,8*aR*)-configuration was assigned to **17** and to the final compounds **18–21**, the latter obtained from α-amino acid with even more hindered side chains. The maintenance of a significant *in vitro* activity of these derivatives would indirectly confirm that C-8*a* would assume the absolute configuration of the eutomer of **1** that has been previously identified following the separation of its enantiomers.^22^ According to this study, the interaction of **1** with the human NPSR would be markedly enantioselective with the *R*-isomer showing a p*K*_B_ value of 8.28 (hNPSR-N^107^) in calcium mobilization experiments, while the *S*-enantiomer would display a considerably reduced potency (p*K*_B_ < 6).

### *In Vitro* Structure–Activity Relationships

**Figure 2:**
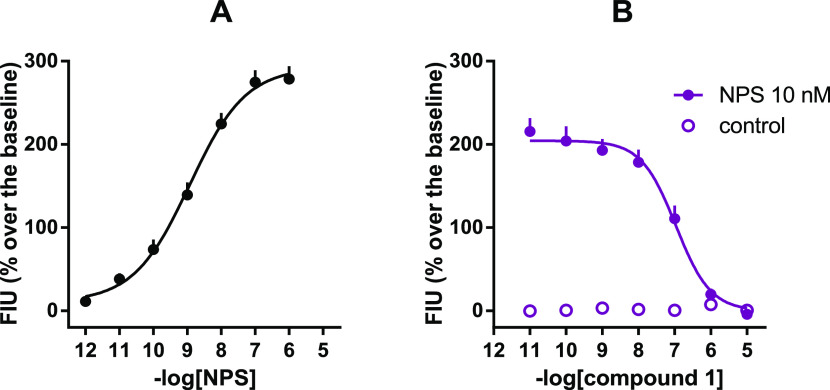
Calcium mobilization assay performed on HEK293_mNPSR_ cells. Concentration–response curve to NPS [panel (A)] and inhibition–response curves to **1** against the stimulatory effect of 10 nM NPS [panel (B)]. Data are mean ± SEM of at least five separate experiments made in duplicate.

In the calcium mobilization assay, NPS increased intracellular calcium levels in a concentration-dependent manner with pEC_50_ and *E*_max_ values of 8.95 and 287 ± 26% over the basal values, respectively. Inhibition response curves to **1** (0.1 nM to 10 μM), used as an internal reference, were performed against the stimulatory effect of 10 nM NPS, approximately corresponding to NPS EC_80_. As shown in Figure 2, compound **1** concentration-dependently inhibited 10 nM NPS stimulatory effects with a p*K*_B_ value of 8.12. These results agree with previously reported data.^11^ The pharmacological activity of compounds **3–21** was evaluated under the same experimental conditions, and the corresponding results are reported in Table 1.

**Table 1:**
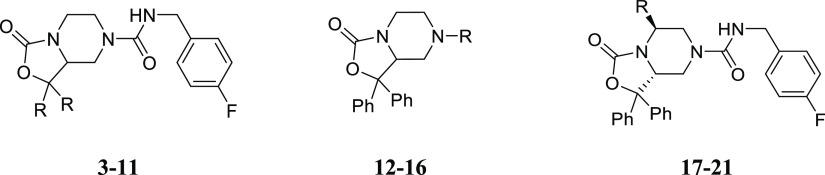

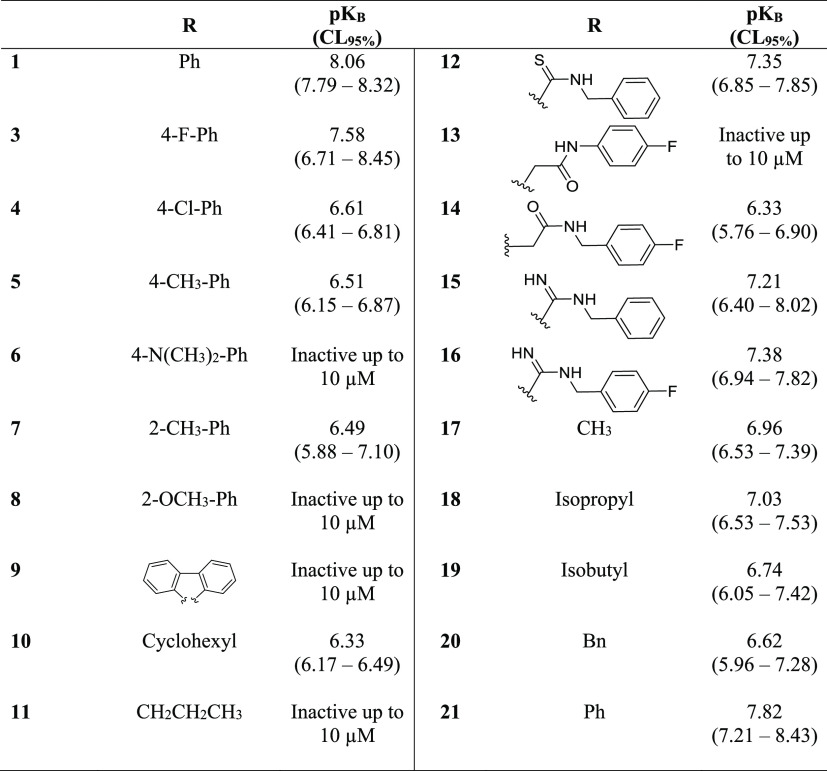
*In Vitro* Pharmacological Activity of Compounds **1**, **3–21** as NPSR Antagonists^a^

a Calcium mobilization assay performed in HEK293_mNPSR_ cells. Data are the mean of five separate experiments made in duplicate.

None of the novel compounds stimulated calcium mobilization up to 10 μM. On the other hand, the substitution or the replacement of the 1,1-diphenyl moiety of **1**, such as in compounds **3–11**, significantly affected the antagonist potency although to a different extent. In particular, the para-substitution of both the aromatic rings at the 1-position was slightly tolerated only in the case of the 4-fluoro derivative **3** which was only 3-fold less potent than **1**, while a progressive reduction of potency was observed by increasing the steric hindrance of the para-substituents. For example, the bulky dimethylamino groups of compound **6** resulted in a complete loss of activity (*K*_B_ > 10 μM). These data suggest that the phenyl rings at the 1-position could occupy the NPSR binding pocket in a region with highly stringent steric requirements.

Our results indicated that also a proper spatial orientation of the geminal phenyl groups relative to the oxazolidinone ring seems to be of particular importance to promote activity. Indeed, the ortho-substitution of the 1,1-diaryl moiety such as in compounds **7** and **8** should induce a conformational distortion with respect to the nonsubstituted **1**, determining a marked or total loss of potency. This observation was further confirmed by the inactivity of compound **9** in which the 1,1-phenyl rings were forced into a coplanar arrangement due to their inclusion in the spiro-fluorene fusion.

The aromaticity of 1,1-substituents seems to be important as well since the 1,1-dicyclohexyl derivative **10** was more than 50-fold less potent than **1**. Even more unfavorable was the replacement of the diaryl template with linear propyl chains (compound **11**, *K*_B_ > 10 μM).

In compounds **12–16**, we explored the effect of a few modifications at the 7-position of the oxazolo[3,4-*a*]pyrazine core that has not been explored before.^23^ In particular, we introduced side chains containing thiourea (**12**), N-substituted acetamide (**13**, **14**) and guanidine (**15**, **16**) functions. In this subset of molecules, compounds **13–16** have been specifically designed to modulate the hydrophilic/lipophilic balance of **1**, which might be important for its *in vivo* effectiveness as suggested in different studies.^10,11^ In particular, it has been demonstrated that **1**, at the high dose of 50 mg/kg, can only partially counteract NPS effects, with different levels of efficacy, depending on the assay used.^10−15^ These findings have been hypothetically attributed to suboptimal physicochemical properties of the compound, in particular, its high lipophilicity.^7^ Thus, in a first attempt to overcome these limits, the acetamide derivatives **13** and **14** have been synthesized as possible bioisosteres of **1** in which a methylene spacer was interposed between the piperazine nitrogen and the carbonyl function of the 7-side chain. The modification was conceived to increase the basicity of the piperazine nitrogen thus opening the possibility to obtain hydrochloride salts with improved water solubility. Nonetheless, the compounds were shown to display very low (**14**, p*K*_B_ = 6.33) or null activity (**13**, *K*_B_ > 10 μM) in the calcium mobilization assay. However, the partial recovery of activity of compound **14**, in which the 4-F phenyl moiety is not directly linked to the amide nitrogen, suggested that also the conformational freedom of this pharmacophoric portion may be important for the interaction with NPSR.

In compounds **15** and **16**, we replaced the urea moiety of **1** with a guanidine function as an alternative strategy to obtain NPSR antagonists with improved hydrophilicity. The fluorinated derivative **16** was conceived as a close analogue of **1** bearing an NH-group in place of the urea oxygen atom. This modification does not interfere with the ability of the ligand to establish polar interactions with the receptor. Of note, compounds incorporating a guanidine moiety have aroused an increasing interest for their potential in the development of novel drugs due to the ability of the guanidinium group to form strong noncovalent interactions and to provide obvious advantages in terms of hydrophilicity.^24−26^ Compound **16** displayed low nanomolar potency in antagonizing the stimulatory activity of NPS with a p*K*_B_ value of 7.38. The nonfluorinated guanidine derivative **15** was slightly less potent (p*K*_B_ = 7.21) indicating some importance of the fluorine atom at the para position of the terminal benzyl moiety.

Finally, we introduced different substitutions at the 5-position of the oxazolo[3,4-*a*]pyrazine core whose effect on NPSR modulation has not been explored before. To this aim, we developed a highly accessible diastereoselective synthesis that provided compounds **17–21** in which the 5-position was functionalized with the side chains of a series of l-amino acids employed as the starting material. The introduction of a −CH_3_ (compound **17**) or an isopropyl chain (compound **18**) determined about a 10-fold reduction of potency if compared to **1**. Even more detrimental was the introduction of a bulkier branched alkyl moiety (**19**) or a benzyl group (**20**). In contrast, the l-phenylglycine derivative **21** showed a recovery in activity becoming the most active compound of the newly reported series.

These data indicated that the 5-position tolerates substitutions with hydrophobic chemical groups of different size generating derivatives with 2 (**21**) to 30 (compound **20**)—fold reduction of potency. Intriguingly, in the latter compounds, a subtle chemical modification such as the introduction of a methylene spacer between C5 and the phenyl ring promoted a consistent reduction of bioactivity. Given these data, we cannot exclude that the C5 phenyl ring of **21** might be recognized by a previously unexplored region of the NPSR binding pocket.

**Figure 3:**
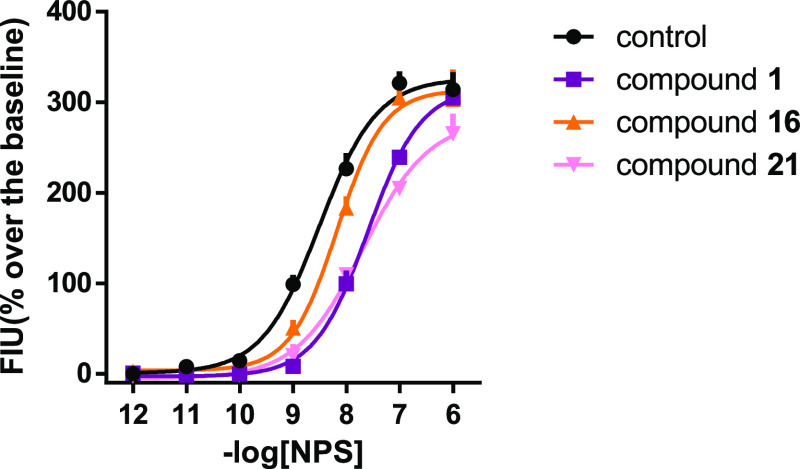
Calcium mobilization assay performed on HEK293_mNPSR_ cells. Concentration–response curve to NPS in the absence and in the presence of 100 nM of **1**, **16**, and **21**. Data are mean ± SEM of at least five separate experiments made in duplicate.

To confirm and better define the antagonist properties of compounds **16** and **21**, the concentration–response curve of NPS has been reassessed in the absence and presence of 100 nM of **1**, **16**, and **21** (Figure 3). **1**, **16**, and **21** shifted the concentration–response curve of NPS to the right without changing its maximal effects. The following p*A*_2_ values have been derived from these experiments: 7.82 (7.40–8.24) for **1**; 7.10 (6.65–7.55) for compound **16**; and 7.59 (7.08–8.10) for compound **21**. Thus, the rank order of potency of these NPSR antagonists is **1** > **21** > **16**. These results are superimposable to those obtained in inhibition experiments.

### Molecular Modeling Studies

**Figure 4:**
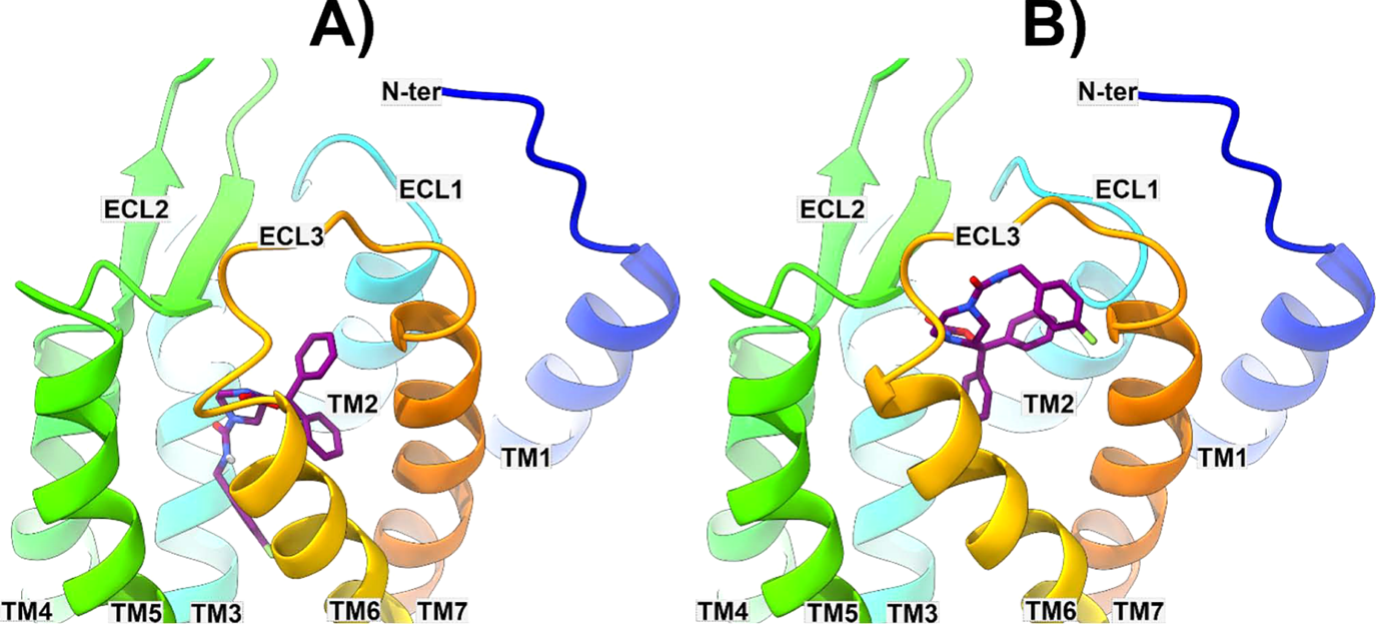
Energy-minimized docked poses of compound **1** in BM1 and BM2 (panels A and B, respectively) in the model of NPSR constructed starting from the human neuropeptide Y Y1 receptor (hNPY1R, PDB code 5ZBH).^29^**1** and the protein are represented as violet sticks and multicolored ribbons, respectively.

To gain major insights into the reasons for the structure–activity relationships (SARs) observed for the newly discovered NPSR ligands, molecular modeling studies were attempted. So far, the three-dimensional (3D) structure of the target receptor has not been determined so that homology-modeling techniques had to be employed to first construct a viable model of NPSR in its inactive state. The available SAR data were obtained from human HEK293 cells expressing the mouse NPSR (mNPSR). On the other hand, the sequence alignment between the human (hNPSR) and mNPSR revealed that the two proteins share 89.22% sequence identity with all the differences residing in the N-terminal region distant from the putative ligand-binding site. Thus, considering the high structural homology of NPSR across the two species, we decided to model the pharmacologically relevant hNPSR in its I107 variant in the present work. In 2010, Dal Ben et al.^27^ published the first model of the two NPSR variants, namely, NPSR-N107 and -I107. In this seminal work, the authors modeled the NPSR receptors starting from the X-ray crystal structure of bovine rhodopsin.^28^ The choice of using this latter structure as a template was dictated by preliminary modeling studies indicating that the NPSR extracellular loop 2 (ECL2) had a propensity to adopt a β-sheet conformation which was partially present in the bovine rhodopsin structure rather than in the structures of the human β1, β2 adrenergic and adenosine A_2A_ receptors that were available at that time. Since then, more than 340 structures of GPCR have been deposited in the protein data bank (PDB) thereby allowing for a re-evaluation of the optimal template to employ in the in silico construction of the NPSR receptor variants. Thus, the hNPSR sequence (Uniprot entry code Q6W5P4) was used to interrogate the PDB and select the solved X-ray structures sharing the highest homology with the target structures. In this analysis, we decided to retain all the structures that shared with NPSR a sequence identity higher than 20%, a sequence coverage higher than 70%, and that were crystallized in their inactive states (i.e., bound to an antagonist ligand). These criteria allowed selecting 7 human GPCR structures in which, interestingly, 6 of them turned out to be receptors for endogenous peptides and 5 of these feature a twisted β-hairpin in the ECL2 region (Table S1). Indeed, the β-hairpin motif is usually found in the ECL2 of peptide-activated GPCRs such as Neuropeptide Y Y1 receptor,^29^ orexin receptor type 1 (OX1R),^30^ chemokine receptor type 4 (CXCR4),^31^ delta,^32^ and kappa^33^ opioid receptors, protease-activated receptor 1 (PAR1),^34^ neurotensin receptor 1 (NTSR1),^35^ endothelin ETB receptor,^36^ and angiotensin receptors AT1^37^ and AT2.^38^ Unfortunately, for one of the selected 7 templates (Table S1), the human CC chemokine receptor type 9 (CCR9), ECL2 was unresolved; thus, this template structure was not considered further. Subsequently, the primary sequences of the remaining six GPCRs were all pairwise aligned to the one of the hNPSR-I107 variant, and the phylogenetic tree was calculated (see Figures S3–S9). Then, these templates were all used to construct six models of hNPSR, one for each template, using the Prime software within the Schrodinger’s Maestro suite. The constructed models were all used to perform docking calculations of all the newly identified analogues employing the Glide program. The results of these simulations were then analyzed in light of the available SAR data. In this step, we first verified whether Glide was able to find a viable binding pose for each active compound reported in Table 1 (namely, **1**, **3–5**, **7**, **10**, **12**, and **14–21**) in each of the NPSR models constructed employing the aforementioned 6 template structures. This first analysis was instrumental for the selection of the best model structure that was able to host all the newly discovered NPSR ligands. In particular, the NPSR model constructed starting from the human neuropeptide Y Y1 receptor (hNPY1R, PDB code 5ZBH)^29^ was the only one able to fulfill the above-mentioned selection criteria. Subsequently, we decided to analyze the docking results achieved for the most potent antagonists **16** and **21** as well as the control compound **1**. Interestingly, for all three ligands, Glide was able to suggest two possible binding poses (i.e. featuring comparable docking scores) in which the ligand pendant benzyl substituent is alternatively pointing downward [inside the transmembrane (TM) bundle] or upward (toward the NPSR extracellular region). In this work, the two alternative docked positions will be referred to as binding mode 1 (BM1) and 2 (BM2), respectively (Figure 4).

**Figure 5:**
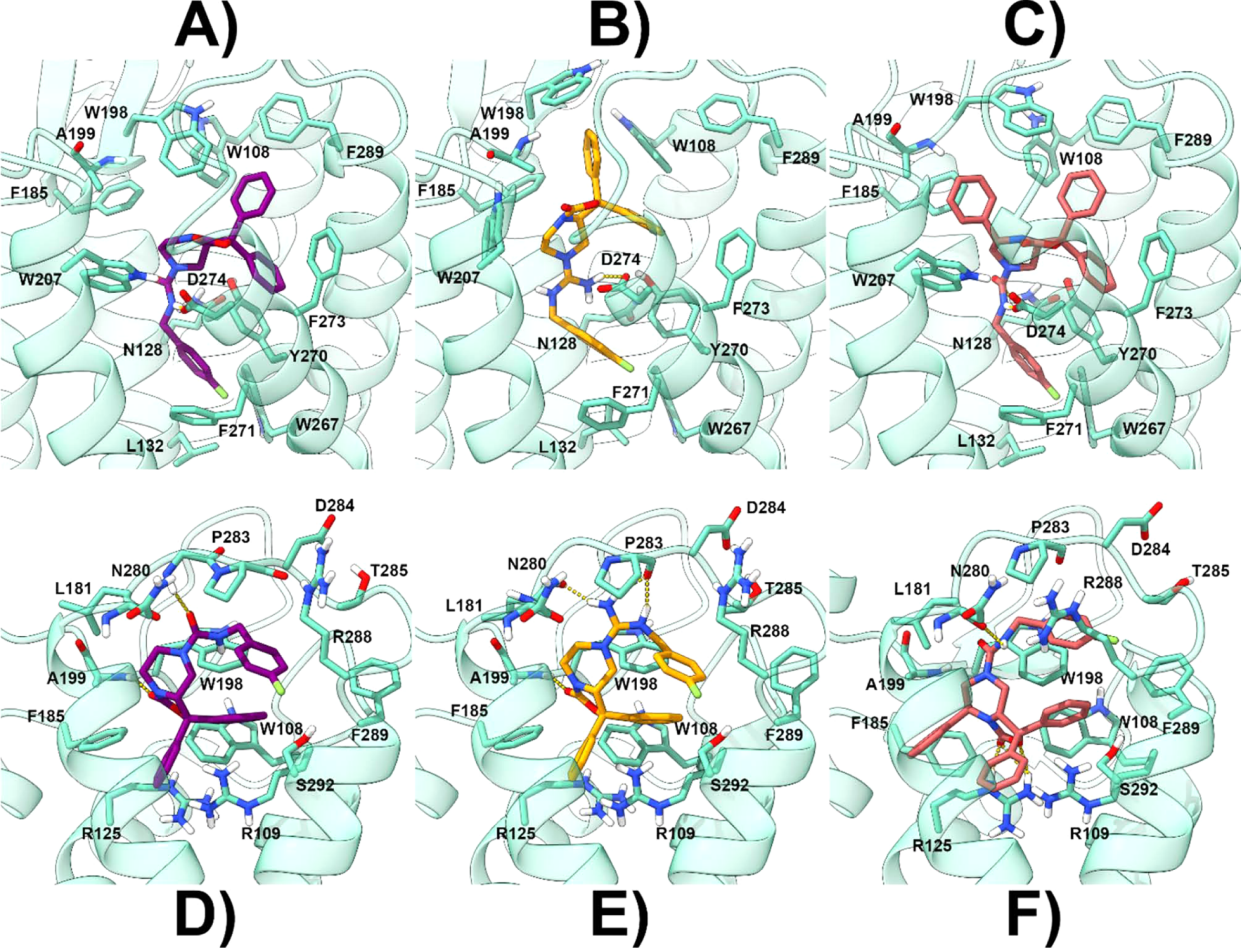
Energy-minimized docked poses of compound **1** [panel (A) for BM1 and (D) for BM2], **16** [panel (B) for BM1 and (E) for BM2], and **21** [panel (C) for BM1 and (F) for BM2] in the model of NPSR constructed starting from the human neuropeptide Y Y1 receptor (hNPY1R, PDB code 5ZBH).^29^**1**, **16**, and **21** are represented as violet, orange, and red sticks, respectively. The protein is represented as cyan ribbons and sticks. H-bonds are represented as dashed yellow lines.

In BM1, the 1,1-diphenyl moiety of **1** is pointing toward TM7 and TM2 and establishes several π–π interactions with aromatic residues present in the outer region of NPSR (W108, W198, F273, and F289) (Figures 4A and 5A). The limited extension of the cleft lodging this moiety should explain why incrementing its steric hindrance has a detrimental effect on the antagonist potency of its analogues (compounds **3–8**). Moreover, the nature of the established ligand–protein interactions (charge transfer contacts) as well as the relative position of the two phenyl rings (not coplanar) explains why compounds **9–11** are less active or devoid of an evident antagonist potency. The **1** bicyclic piperazine core orients its pendant benzylamide chain inside the TM bundle between TM3 and TM6 where its NH forms a charged-reinforced H-bond with D274 and the terminal fluorophenyl ring is lodged in a well-defined lipophilic gorge establishing π–π interactions, reinforced by the electron-withdrawing effect of the fluorine atom, with W267, Y270, F271, and van der Waal contacts with L132 (Figure 5A). The tight interactions established by the benzylamide chain in this receptor region would explain why modifications in this position result in a reduction (compound **14**) or abrogation (compound **13**) of the antagonist potency thereby indicating a limited tolerance for 7-position substituents.^23^

Almost the same binding orientation was also found for **16** with all the aforementioned interactions being maintained (Figure 5B). Here, the protonated guanidine group is now engaged in an ionic interaction with the D274 side chain. **21** is also predicted to adopt BM1 where the presence of the additional phenyl ring on the core structure allows to form supplementary charge-transfer and lipophilic interactions with F185, W198, A199, and W207 (Figure 5C). These contacts should explain why the substitution of the phenyl ring with alkyl or benzyl chains (compounds **17–20**) still allows observing an antagonist activity at NPSR, although resulting in less potent antagonists if compared to **21**.

In BM2, the 1,1-diphenyl moiety of **1** is predicted to point toward the inner part of NPSR making contact with TM2, TM6, and TM7 (Figure 4B). In this position, this moiety established π–π and cation−π contacts with W108 and R109, respectively (Figure 5D). The carbonyl oxygen of the bicyclic piperazine scaffold accepts an H-bond from the A199 backbone NH while the pendant fluorobenzylamide chain laced beneath ECL3 (Figure 4B) in a cleft lined by D284, T285, R288, F289, and S292. Almost the same interaction pattern is also predicted for **16** in BM2 (Figure 5E), and the presence of the protonated guanidine group allows the ligands to establish charge-reinforced H-bonds with N280 and P283 backbone COs as well as a cation−π interaction with W198. **21** recapitulates the same interactions established by **1** in BM2 (Figure 5F) and takes advantage of its additional phenyl ring in position 7 of the core scaffold to establish a π–π interaction with F185. Also in this case, BM2 would match the SAR data acquired in this manuscript as well as the ones already present in the literature.^23^

The binary complexes calculated using the docking program for **1**, **16**, and **21** in both BM1 and BM2 were then subjected to 100 ns molecular dynamics (MD) simulations with Desmond^39^ to refine the predicted binding geometries. Most importantly, given the dichotomy of binding modes predicted for these ligands, results of MD simulations could suggest a preferential binding orientation for each ligand. Analysis of the 6 MD simulations was attained through the Desmond SID tool which allowed us to analyze the ligand–receptor interactions during the MD trajectory. Attention was given to the ligand root-mean-square fluctuation (RMSF) (Figures S10–S15) and the stability of the ligand–residue interactions (Figures S16–S27).

The ligand RMSF was useful for characterizing changes in the ligand atom positions during the MD. Analysis of this parameter demonstrated that in BM1 and BM2, the three ligands display a different degree of flexibility. In particular, regardless of the adopted binding mode, the pendant fluorobenzylamide chain is the most flexible part of the molecule with BM1 being more stable than BM2 (see Figures S10–S15). In the latter binding position, the fluorobenzylamide moiety rapidly loses its interactions with the residues belonging to ECL3 to point toward the external part of the receptor without taking stable contacts with any NPSR residue. This is further outlined by plotting the most frequent (>30%) ligand–protein contacts (Figures S16–S27) showing that for the three ligands in BM2, the benzyl chain is always solvent exposed. On the contrary, in BM1, the same portion remains anchored to the receptor although experiencing a partial relocation during the MD. All in all, MD results would suggest that only in BM1, the fluorobenzylamide chain would play a role in the ligand–receptor recognition as underscored by the experimental SAR data^23^ available for this ligand moiety, thereby suggesting that **1**, **16,** and **21** should adopt this binding orientation. Plotting of the ligand–receptor interactions also shows that while binding of **1** is mainly governed by hydrophobic and charge-transfer contacts with the receptor, **16** is stably anchored to NPSR also through the polar interaction with D274 while **21** finds additional contacts with W207 and F185.

### *In Vivo* Characterization in the Mouse Locomotor Activity Test

**Figure 6:**
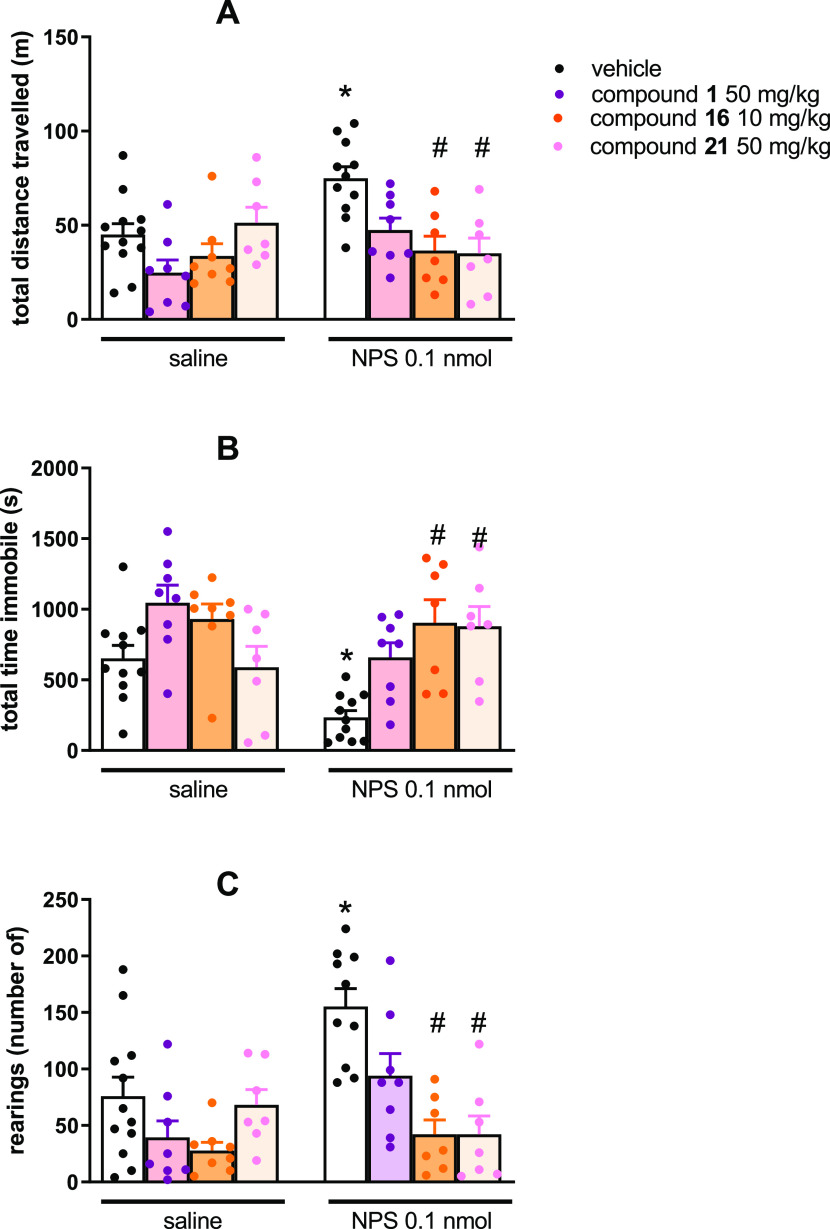
Effects of NPS, **1**, **16**, and **21** on mouse locomotor activity. The cumulative effects exerted on distance traveled are shown in panel (A), while the total time immobile and number of rearings over the 30 min observation period are shown in panels (B,C), respectively. Data are mean ± SEM of four separate experiments (vehicle + saline, 12 mice; **1** + saline, 8 mice; **16** + saline, 8 mice; **21** + saline, 7 mice; vehicle + NPS, 11 mice, **1** + NPS, 8 mice; **16** + NPS, 7 mice; and **21** + NPS, 7 mice). The two-way ANOVA NPS × antagonist revealed for the total distance traveled, an effect of NPS *F*(1,59) = 6.63 and of the interaction NPS × antagonist *F*(3,59) = 4.48; for the immobility time, an effect of NPS *F*(1,59) = 8.09 and of the interaction NPS × antagonist *F*(3,59) = 4.26; for the number of rearings, an effect of NPS *F*(1,59) = 7.35, of antagonist *F*(3,59) = 10.65, and of the interaction NPS × antagonist *F*(3,59) = 4.32. *p < 0.05 vs saline, #*p* < 0.05 vs vehicle according to Bonferroni’s test for multiple comparisons.

Among the synthesized molecules, **16** and **21** have been selected to be tested *in vivo* in the mouse locomotor activity assay. As shown in Figure 6 and in line with previous findings,^11,40−42^ NPS injected by the i.c.v. route at 0.1 nmol concentration was able to stimulate mouse locomotor activity by increasing the distance traveled (panel A) and the number of rearings (panel C) and reducing the immobility time (panel B) with statistically significant effects. **1** (50 mg/kg) did not significantly modify mice locomotor activity and only partially counteracted NPS-induced stimulant effects, confirming previous studies.^10,11^ All mice treated with 50 mg/kg of **16** displayed an important reduction of locomotor activity (data not shown); thus, in the present study, the 10 mg/kg dose was used for this compound. The doses of 10 mg/kg for **16** and 50 mg/kg for **21** did not elicit by themselves statistically significant effects on mouse locomotor activity (Figure 6).

This result is in line with previous studies performed with different NPSR antagonists^10,11,41^ and with the lack of locomotor phenotype of NPSR knockout mice,^43−45^ collectively suggesting that the endogenous NPS does not control mouse locomotor activity in the open field. When administered 30 min before NPS, both **16** and **21** were able to completely block the stimulant effects of the peptide. Compound **21** was *in vivo* slightly more effective than **1** when injected intraperitoneally (ip) at the same dose (50 mg/kg). Compound **16**, despite its lower potency *in vitro*, was at least 5-fold more potent than **1***in vivo*. It must be remarked that compound **2** (see the Introduction section) was shown to exert a significant blockade of NPS-induced locomotor activity at an i.p. dose of 50 mg/kg, thus being equipotent to the parental compound **1**.^16^ Of note, the ability of both **16** and **21** to completely block the stimulant NPS effects confirms previous studies demonstrating that the stimulant NPS effects are selectively due to the activation of the NPSR receptor (see Tables 1 and 2 of the recent review by Ruzza et al.).^7^ It is worth noting that at the higher doses tested, compound **1** was able to only partially block NPS effects. This may somewhat limit the usefulness of compound **1** as a research tool to explore the biological actions modulated by the endogenous NPS. Both compounds **16** and **21** seem to overcome this limitation; in fact, at doses statistically inactive, they were able to fully block NPS stimulant effects. This feature, that is, complete NPSR occupation and blockage, makes these compounds essential pharmacological tools for investigating those conditions in which the endogenous NPS/NPSR signaling is activated, for example, cocaine/alcohol seeking and relapse.^46−48^ On the other hand, in proposing compound **16** as an innovative research tool, we should also underline that selectivity concerns must be taken into account. In fact, at the high dose of 50 mg/kg, **16** strongly reduces mouse locomotor activity. This is most probably an off-target effect since NPSR selective peptide antagonists did not modify mouse locomotion at doses able to completely block NPS stimulatory effects,^41^ and NPSR(−/−) mice did not display a locomotor phenotype in this assay.^43^ Thus, the high potency and antagonist effectiveness of compound **16** is associated with somewhat limited selectivity of action. Further studies, for example, CEREP receptogram, are needed to eventually identify the mechanisms involved in the putative off-target effects of compound **16** at high doses. Based on these considerations, we certainly recommend the use of compound **16** but with special caution in selecting the range of doses to be used for NPSR physiopathological investigations. The *in vivo* action of **21** reflects the *in vitro* potency of the compound that was very similar to that of the reference tool **1**. On the other hand, the improved *in vivo* antagonist effectiveness of **16**, deriving from the bioisosteric replacement of the urea function of **1** with a guanidine moiety, may be attributed to its relatively higher hydrophilic properties that could compensate for the slight loss of *in vitro* potency. Lipophilicity is indeed considered one of the most important physicochemical properties to be addressed for drug design purposes. Typically, highly hydrophilic compounds suffer from poor membrane permeability and faster renal clearance. On the other hand, water solubility and metabolism are more likely to be compromised at high lipophilicity values.^49^ Noteworthily, the optimum region of lipophilicity for candidate drug molecules has been generally suggested to lie within a narrow range of log *D*_7.4_ that has been approximately determined between 1 and 3.^49^ Thus, the improved *in vivo* potency of **16** (Clog *P* = 3.43 ± 0.89, ACDLabs; Clog *D*_7.4_ = 1.87, ChemAxon predictor) with respect to the known NPSR antagonist **1** (Clog *P* = 4.32 ± 0.86, ACDLabs; Clog *D*_7.4_ = 4.05, ChemAxon predictor) can be reasonably justified in light of the optimization of lipophilicity parameters that are closer to the recommended values. This could also account for the lower *in vivo* potency of compound **21** (Clog *P* = 6.18 ± 0.87, ACDLabs; Clog *D*_7.4_ = 5.83, ChemAxon predictor) despite its higher *in vitro* activity. However, we would like to underline the fact that these hypotheses are only supported by theoretical calculations; firm conclusions on this issue can be drawn only after performing experiments investigating the pharmacokinetic profile of compound **16** eventually in comparison with compounds **1** and **21**.

## Conclusions

The therapeutic potential of selective NPSR ligands in psychiatric disorders is supported by a series of preclinical studies.^7^ In particular, NPSR antagonists were shown to reduce cocaine/alcohol seeking and relapse in animal models, and this makes them potentially useful for treating drug addiction. Patients suffering from such a condition typically show resistance to the very few treatments currently available. Thus, the identification of innovative drugs able to ameliorate these conditions, which are essentially untreated, is still urgent. Additionally, it can be speculated that NPSR antagonists may be useful also for the treatment of other types of drug abuse, although no preclinical data are currently available. NPSR antagonists may open new perspectives for addressing this unmet clinical need. Moreover, NPSR antagonists still represent important research tools to investigate the neurobiology of the NPS/NPSR system and to study those biological functions for which the NPSergic tone is important. However, even though *in vitro* potent NPSR antagonists have been reported, NPSR antagonists with a good *in vivo* pharmacological profile are still missing. This is probably due to pharmacokinetic issues and represents an undeniable limit for preclinical studies on the NPSergic system aimed at translating results from basic pharmacology into clinical utility. In the present study, the *in vitro* pharmacological activity of a new series of oxazolo[3,4-*a*]pyrazines as NPSR antagonists was investigated and a molecular-modeling study helped to rationalize the resulting SARs. The most promising compounds (**16** and **21**) in terms of *in vitro* potency and/or drug-likeness properties have been also evaluated *in vivo* for their capability to counteract NPS-induced stimulant effects on mouse locomotor activity. Our findings demonstrated that these compounds behave *in vitro* as pure NPSR antagonists with nanomolar potency in inhibiting the NPS stimulatory effects in the calcium mobilization assay (p*K*_B_ values of 7.38 and 7.82 for **16** and **21**, respectively). Importantly, the guanidine derivative **16** exhibited a significantly (5-fold) improved potency and increased antagonist effectiveness *in vivo* when compared to the reference compound **1**, although this is associated with somewhat reduced selectivity of action. Collectively, our efforts can be considered an important advancement in this research field culminating in the identification of a new pharmacological tool that combines *in vitro* and *in vivo* potency in blocking NPSR. This could be useful to investigate possible pharmacological treatments in all the pathological conditions in which the endogenous NPSergic system is activated.

## Experimental Section

### Chemistry

#### Materials and Methods

The chemicals, including the 2-(4-bromomethyl-phenoxy)ethyl polystyrene HL resin for solid-phase synthesis, were purchased from Fluorochem, Novabiochem Iris Biotech GmbH, or Sigma-Aldrich. Reaction progress and product mixtures were monitored by thin-layer chromatography (TLC) on silica gel (precoated F254 Macherey-Nagel plates) and visualized with a UV lamp (254 nm light source). Compounds were purified through silica gel flash chromatography (silica gel 60, 40–63 μm) using appropriate eluent mixtures or on a reverse-phase Waters Prep 600 HPLC system equipped with a Jupiter column C18 (250 × 30 mm, 300 Å, 15 μm spherical particle size). Reverse-phase purification of crude compounds was carried out using a gradient of CH_3_CN/H_2_O [with 0.1% trifluoroacetyl (TFA)] programed time by time, with a flow rate of 20 mL/min and a UV detector with a wavelength of 220 nm. Analytical HPLC analyses were performed on a Beckman 116 liquid chromatograph equipped with a Beckman 166 diode array detector. Analytical purity of the final compounds were assessed using a XBridge C18 column (4.6 × 150 mm, 5 μm particle size) at a flow rate of 0.7 mL/min with a linear gradient from 100% of solvent A (H_2_O + 0.1% TFA) to 100% of solvent B (CH_3_CN + 0.1% TFA) over 25 min. Analytical determinations were reported as column retention time (*T*_R_) in minutes, and the purity of final compounds was >95% as determined by HPLC analysis carried out at a wavelength of 220 nm. Mass spectra were recorded with a Waters ESI Micromass ZQ dissolving the samples in a solution of H_2_O/CH_3_CN/TFA (40:60:0.1). Melting points for purified products **3–21** were determined using glass capillaries on a Stuart Scientific electrothermal apparatus SMP3 and are uncorrected. NMR analyses were performed in CDCl_3_ or DMSO-*d*_6_ at ambient temperature using a Varian 200 or 400 MHz spectrometer. Chemical shifts (δ) are reported in parts per million (ppm) using the peak of tetramethylsilane as an internal standard in deuterated solvents, and coupling constants (*J*) are reported in Hertz. Splitting patterns are designed as s, singlet; d, doublet; t, triplet; q, quartet; m, multiplet; and b, broad. Optical rotations were measured on a Jasco P-2000 polarimeter dissolving the samples in methanol, with a path length of 1 dm, using sodium D line, 589 nm.

#### General Procedure for the Synthesis of **25a–j**

Tetramethylethylenediamine (TMEDA, 2.7 mmol) was added under an argon atmosphere at room temperature to a stirring solution of **23**(10) (1 mmol) in freshly distilled tetrahydrofuran (THF) (5 mL). After cooling at −78 °C, *sec*-BuLi (2.7 mmol) was added and the reaction was allowed to reach −30 °C over 2 h. A solution of appropriate benzophenone **24a–j** (2 mmol) in THF (5 mL) was added and the reaction solution was left stirring at −30 °C for 30 min, then it was slowly warmed to rt and stirred for 16 h. The reaction was quenched with a saturated solution of NH_4_Cl (15 mL), and the solvents were concentrated under vacuum to half volume and the aqueous phase was extracted with EtOAc (3 × 15 mL). The organic layers were combined, dried over Na_2_SO_4_, and the solvent was removed under vacuum. The resulting crude product was purified by flash column chromatography on silica gel using a mixture of EtOAc/PEt 1:2 as the eluent.

##### 7-Benzyl-1,1-bis(4-fluorophenyl)tetrahydro-1*H*-oxazolo[3,4-*a*]pyrazin-3(5*H*)-one (**25a**)

White solid (227 mg, 54% yield). ^1^H NMR (200 MHz, CDCl_3_): δ (ppm) 7.70–7.59 (m, 2H); 7.40–7.16 (m, 11H); 4.66 (dd, *J* = 8.0, 4.0 Hz, 1H); 3.62–3.49 (m, 2H); 3.38–3.21 (m, 1H); 3.16–2.98 (m, 1H); 2.63–2.58 (m, 2H); 1.88–1.69 (m, 1H); 1.52–1.38 (m, 1H). MS (ESI): *m*/*z* calcd for C_25_H_23_F_2_N_2_O_2_ [M + H]^+^, 421.47; found, 421.03.

##### 7-Benzyl-1,1-bis(4-chlorophenyl)tetrahydro-1*H*-oxazolo[3,4-*a*]pyrazin-3(5*H*)-one (**25b**)

White solid (200 mg, 44% yield). ^1^H NMR (200 MHz, CDCl_3_): δ (ppm) 7.44–7.18 (m, 13H); 4.63–4.20 (m, 1H); 3.82 (dd, *J* = 8.0, 4.0 Hz, 1H); 3.44–3.04 (m, 2H); 2.76 (d, *J* = 10.6 Hz, 1H); 2.53 (d, *J* = 11.1 Hz, 1H); 2.01–1.88 (m, 1H); 1.60 (t, *J* = 7.4 Hz, 2H). MS (ESI): *m*/*z* calcd for C_25_H_23_Cl_2_N_2_O_2_ [M + H]^+^, 454.37; found, 454.26.

##### 7-Benzyl-1,1-di-*p*-tolyltetrahydro-1*H*-oxazolo[3,4-*a*]pyrazin-3(5*H*)-one (**25c**)

Pale yellow solid (260 mg, 63% yield). ^1^H NMR (200 MHz, CDCl_3_): δ (ppm) 7.44 (d, *J* = 8.4 Hz, 2H); 7.31–7.12 (m, 11H); 4.60 (dd, *J* = 11.1, 3.7 Hz, 1H); 3.61–3.46 (m, 2H); 3.34 (s, 1H); 3.16–2.96 (m, 1H); 2.68–2.52 (m, 2H); 2.26 (d, *J* = 3.4 Hz, 6H); 1.84–1.65 (m, 1H); 1.58–1.39 (m, 1H). MS (ESI): *m*/*z* calcd for C_27_H_29_N_2_O_2_ [M + H]^+^, 413.54; found, 413.46.

##### 7-Benzyl-1,1-bis(4-(dimethylamino)phenyl)tetrahydro-1*H*-oxazolo[3,4-*a*]pyrazin-3(5*H*)-one (**25d**)

Dark blue solid (169 mg, 36% yield). ^1^H NMR (200 MHz, DMSO-*d*_6_): δ (ppm) 7.31–7.20 (m, 7H); 6.99 (d, *J* = 8.6 Hz, 2H); 6.66 (t, *J* = 8.2 Hz, 4H); 4.45 (d, *J* = 8 Hz, 1H); 3.53 (t, *J* = 4 Hz, 2H); 3.42–3.31 (m, 1H); 3.01 (d, *J* = 12 Hz, 1H); 2.87 (s, 12H); 2.63–2.41 (m, 2H); 1.78 (t, *J* = 12 Hz, 1H); 1.48 (t, *J* = 12 Hz, 1H). MS (ESI): *m*/*z* calcd for C_29_H_35_N_4_O_2_ [M + H]^+^, 471.63; found, 471.44.

##### 7-Benzyl-1,1-di-*o*-tolyltetrahydro-1*H*-oxazolo[3,4-*a*]pyrazin-3(5*H*)-one (**25e**)

White solid (82 mg, 28% yield). ^1^H NMR (200 MHz, CDCl_3_): δ (ppm) 7.89–7.79 (m, 1H); 7.67–7.55 (m, 1H); 7.40–6.96 (m, 11H); 4.88 (d, *J* = 10.5 Hz, 1H); 3.86 (d, *J* = 11.2 Hz, 2H); 3.52 (d, *J* = 12.1 Hz, 1H); 3.32–3.10 (m, 2H); 2.79–2.58 (m, 1H); 2.11–1.95 (m, 2H); 1.79 (d, *J* = 9.8 Hz, 6H). MS (ESI): *m*/*z* calcd for C_27_H_29_N_2_O_2_ [M + H]^+^, 413.54; found, 413.44.

##### 7-Benzyl-1,1-bis(2-methoxyphenyl)tetrahydro-1*H*-oxazolo[3,4-*a*]pyrazin-3(5*H*)-one (**25f**)

White solid (169 mg, 38% yield). ^1^H NMR (200 MHz, CDCl_3_): δ (ppm) 7.49–7.21 (m, 8H); 6.96–6.75 (m, 5H); 4.54 (d, *J* = 8 Hz, 1H); 3.90 (dd, *J* = 8.0, 4.0 Hz, 1H); 3.51 (s, 6H); 3.40–3.05 (m, 2H); 2.81–2.52 (m, 2H); 2.20–2.01 (m, 1H); 1.75–1.56 (m, 2H). MS (ESI): *m*/*z* calcd for C_27_H_29_N_2_O_4_ [M + H]^+^, 445.54; found, 445.30.

##### 7′-Benzyl-6′,7′,8′,8a′-tetrahydrospiro[fluorene-9,1′-oxazolo[3,4-*a*]pyrazin]-3′(5′*H*)-one (**25g**)

Yellow solid (279 mg, 73% yield). ^1^H NMR (200 MHz, CDCl_3_): δ (ppm) 7.85–7.79 (m, 4H); 7.61–7.09 (m, 9H); 4.20 (dd, *J* = 10.6, 3.2 Hz, 1H); 3.72 (d, *J* = 11.1 Hz, 1H); 3.43–3.36 (m, 2H); 3.20–3.05 (m, 1H); 2.80 (d, *J* = 8.2 Hz, 1H); 2.31–1.85 (m, 3H). MS (ESI): *m*/*z* calcd for C_25_H_23_N_2_O_2_ [M + H]^+^, 383.47; found, 383.29.

##### 7-Benzyl-1,1-dicyclohexyltetrahydro-1*H*-oxazolo[3,4-*a*]pyrazin-3(5*H*)-one (**25h**)

White solid (162 mg, 41% yield). ^1^H NMR (200 MHz, CDCl_3_): δ (ppm) 7.40–7.22 (m, 4H); 7.02 (s, 1H); 3.78–3.64 (m, 2H); 3.58–3.42 (m, 2H); 3.34 (s, 1H); 2.90–2.61 (m, 3H) 2.24 (t, *J* = 12 Hz, 1H); 1.97–1.41 (m, 13H); 1.28–0.98 (m, 9H). MS (ESI): *m*/*z* calcd for C_25_H_37_N_2_O_2_ [M + H]^+^, 397.58; found, 397.31.

##### 7-Benzyl-1,1-dipropyltetrahydro-1*H*-oxazolo[3,4-*a*]pyrazin-3(5*H*)-one (**25i**)

White solid (136 mg, 43% yield). ^1^H NMR (200 MHz, CDCl_3_): δ (ppm) 7.76 (d, *J* = 6.8 Hz, 1H); 7.44–7.25 (m, 4H); 4.80–4.44 (m, 2H); 4.30–4.00 (m, 2H); 3.67 (d, *J* = 12.0 Hz, 1H); 3.29–3.15 (m, 1H); 3.00–2.56 (m, 3H); 1.61–1.00 (m, 8H); 0.94–0.84 (m, 6H). MS (ESI): *m*/*z* calcd for C_19_H_29_N_2_O_2_ [M + H]^+^, 317.45; found, 317.46.

##### 7-Benzyl-1,1-diphenyltetrahydro-1*H*-oxazolo[3,4-*a*]pyrazin-3(5*H*)-one (**25j**)

White solid (277 mg, 72% yield). ^1^H NMR (200 MHz, CDCl_3_): δ (ppm): 7.60–7.49 (m, 1H); 7.38–7.16 (m, 14H); 4.52 (dd, *J* = 8.0, 4.0 Hz, 1H); 3.72–3.59 (m, 2H); 3.48–3.21 (m, 1H); 3.16–2.98 (m, 1H); 2.63–2.58 (m, 2H); 1.88–1.69 (m, 1H); 1.52–1.38 (m, 1H). MS (ESI): *m*/*z* calcd for C_25_H_25_N_2_O_2_ [M + H]^+^, 385.49; found, 385.28.

#### General Procedure for the Synthesis of **26a–j**

9-Fluorenylmethoxycarbonyl chloride (FmocCl, 1.1 mmol) was added to a solution of **25a–j** (1 mmol) in CH_3_CN (5 mL). The reaction solution was heated at 90 °C for 5 h and then stirred at room temperature for 18 h. The solvent was evaporated giving a residue that was dissolved in EtOAc (30 mL), and the resulting organic phase was washed with water (3 × 15 mL), dried over anhydrous Na_2_SO_4_, and concentrated to dryness. All crude residues were finally purified via flash column chromatography on a silica gel using the appropriate mixture of petroleum ether and EtOAc as an eluent (see below).

##### (9*H*-Fluoren-9-yl)methyl 1,1-Bis(4-fluorophenyl)-3-oxotetrahydro-3*H*-oxazolo[3,4-*a*]pyrazine-7(1*H*)-carboxylate (**26a**)

White solid (448 mg, 81% yield). Eluent for chromatography purification: EtOAc/PEt 1:1; ^1^H NMR (200 MHz, DMSO-*d*_6_): δ (ppm) 7.94–7.84 (m, 2H); 7.62–7.18 (m, 14H); 4.65–4.39 (m, 3H); 4.25 (t, *J* = 5.4 Hz, 1H); 3.82 (d, *J* = 10.5 Hz, 1H); 3.56 (dd, *J* = 10.6, 2.2 Hz, 1H); 3.05–2.85 (m, 1H); 2.83–2.64 (m, 2H); 2.31–2.16 (m, 1H). MS (ESI): *m*/*z* calcd for C_33_H_27_F_2_N_2_O_4_ [M + H]^+^, 553.59; found, 553.57.

##### (9*H*-Fluoren-9-yl)methyl 1,1-Bis(4-chlorophenyl)-3-oxotetrahydro-3*H*-oxazolo[3,4-*a*]pyrazine-7(1*H*)-carboxylate (**26b**)

White solid (298 mg, 51% yield). Eluent for chromatography purification: EtOAc/PEt 1:2; ^1^H NMR (200 MHz, DMSO-*d*_6_): δ (ppm) 7.90 (t, *J* = 6.8 Hz, 2H); 7.59–7.28 (m, 14H); 4.43–4.39 (m, 2H); 4.24 (t, *J* = 5.6 Hz, 2H); 3.82 (d, *J* = 11.2 Hz, 1H); 3.56 (d, *J* = 10.6 Hz, 1H); 3.19 (s, 1H); 3.07–2.64 (m, 2H); 2.24 (t, *J* = 12.4 Hz, 1H). MS (ESI): *m*/*z* calcd for C_33_H_27_Cl_2_N_2_O_4_ [M + H]^+^, 586.49; found, 586.63.

##### (9*H*-Fluoren-9-yl)methyl 3-Oxo-1,1-di-*p*-tolyltetrahydro-3*H*-oxazolo[3,4-*a*]pyrazine-7(1*H*)-carboxylate (**26c**)

White solid (327 mg, 60% yield). Eluent for chromatography purification: EtOAc/PEt 1:4; ^1^H NMR (200 MHz, DMSO-*d*_6_): δ (ppm) 7.90 (t, *J* = 7.4 Hz, 2H); 7.59 (d, *J* = 7.0 Hz, 2H); 7.44–7.16 (m, 10H); 6.99 (s, 2H); 4.77–4.59 (m, 1H); 4.50–4.35 (m, 1H); 4.26 (s, 1H); 4.16–4.09 (m, 1H); 3.80 (d, *J* = 10.6 Hz, 1H); 3.53 (dd, *J* = 11.2, 1.8 Hz, 1H); 3.22–3.05 (m, 1H); 2.95–2.58 (m, 2H); 2.30 (d, *J* = 4.6 Hz, 6H); 2.20–2.08 (m, 1H). MS (ESI): *m*/*z* calcd for C_35_H_33_N_2_O_4_ [M + H]^+^, 545.66; found, 545.59.

##### (9*H*-Fluoren-9-yl)methyl 1,1-Bis(4-(dimethylamino)phenyl)-3-oxotetrahydro-3*H*-oxazolo[3,4-*a*]pyrazine-7(1*H*)-carboxylate (**26d**)

Pink solid (380 mg, 63% yield). Eluent for chromatography purification: EtOAc/PEt 1:4; ^1^H NMR (200 MHz, DMSO-*d*_6_): δ (ppm) 7.88 (t, *J* = 6.6 Hz, 2H); 7.59 (d, *J* = 7.2 Hz, 2H); 7.44–7.32 (m, 5H); 7.11 (s, 2H); 6.75–6.63 (m, 5H); 4.79–4.61 (m, 1H); 4.52–4.33 (m, 1H); 4.26 (t, *J* = 5.6 Hz, 1H); 4.01–3.90 (m, 1H); 3.82–3.60 (m, 1H); 3.53 (d, *J* = 10.6 Hz, 1H); 3.22–3.05 (m, 1H); 3.11–2.98 (m, 1H); 2.90 (d, *J* = 4.8 Hz, 12H); 2.83–2.77 (m, 1H); 2.22–2.06 (m, 1H). MS (ESI): *m*/*z* calcd for C_37_H_39_N_4_O_4_ [M + H]^+^, 603.74; found, 603.51.

##### (9*H*-Fluoren-9-yl)methyl 3-Oxo-1,1-di-*o*-tolyltetrahydro-3*H*-oxazolo[3,4-*a*]pyrazine-7(1*H*)-carboxylate (**26e**)

White solid (408 mg, 75% yield). Eluent for chromatography purification: EtOAc/PEt 3:7; ^1^H NMR (200 MHz, DMSO-*d*_6_): δ (ppm) 7.89–7.79 (m, 2H); 7.67–7.55 (m, 2H); 7.40–6.96 (m, 12H); 2.51–4.07 (m, 1H); 4.88 (d, *J* = 10.5 Hz, 1H); 3.86 (d, *J* = 11.2 Hz, 2H); 3.52 (d, *J* = 12.1 Hz, 1H); 3.32–3.10 (m, 2H); 2.79–2.58 (m, 1H); 2.11–1.95 (m, 2H); 1.79 (d, *J* = 9.8 Hz, 6H). MS (ESI): *m*/*z* calcd for C_35_H_33_N_2_O_4_ [M + H]^+^, 545.66; found, 545.25.

##### (9*H*-Fluoren-9-yl)methyl 1,1-Bis(2-methoxyphenyl)-3-oxotetrahydro-3*H*-oxazolo[3,4-*a*]pyrazine-7(1*H*)-carboxylate (**26f**)

White solid (363 mg, 63% yield). Eluent for chromatography purification: EtOAc/PEt 1:1; ^1^H NMR (200 MHz, CDCl_3_): δ (ppm): 7.72 (d, *J* = 7.2 Hz, 2H); 7.48–7.29 (m, 10H); 7.01–6.88 (m, 4H); 4.58–4.35 (s, 2H); 4.24–4.06 (m, 4H); 4.01–3.36 (m, 7H); 2.89–2.69 (m, 2H); 2.64–2.42 (m, 1H). MS (ESI): *m*/*z* calcd for C_35_H_33_N_2_O_6_ [M + H]^+^, 577.66; found, 577.65.

##### (9*H*-Fluoren-9-yl)methyl 3′-Oxo-5′,6′,8′,8a′-tetrahydro-3′*H*,7′*H*-spiro[fluorene-9,1′-oxazolo[3,4-*a*]pyrazine]-7′-carboxylate (**26g**)

White solid (283 mg, 55% yield). Eluent for chromatography purification: EtOAc/PEt 1:4; ^1^H NMR (200 MHz, DMSO-*d*_6_): δ (ppm) 7.95 (s, 2H); 7.90–7.02 (m, 14H); 4.56–4.25 (m, 2H); 4.19–4.05 (s, 1H); 3.86 (d, *J* = 12.40 Hz, 2H); 3.69 (d, *J* = 12.90 Hz, 2H); 2.98–2.64 (m, 3H). MS (ESI): *m*/*z* calcd for C_33_H_27_N_2_O_4_ [M + H]^+^, 515.59; found, 515.50.

##### (9*H*-Fluoren-9-yl)methyl 1,1-Dicyclohexyl-3-oxotetrahydro-3*H*-oxazolo[3,4-*a*]pyrazine-7(1*H*)-carboxylate (**26h**)

White solid (434 mg, 82% yield). Eluent for chromatography purification: EtOAc/PEt 1:4; ^1^H NMR (200 MHz, DMSO-*d*_6_): δ (ppm) 7.88 (d, *J* = 7.5 Hz, 2H); 7.65 (t, *J* = 7.0 Hz, 2H); 7.42 (t, *J* = 7.4 Hz, 2H); 7.33 (dd, *J* = 13.8, 7.1 Hz, 2H); 4.63–4.42 (m, 2H); 4.28 (t, *J* = 5.6 Hz, 1H); 3.94–3.58 (m, 2H); 3.50–3.18 (m, 3H); 2.98–2.63 (m, 2H); 1.78–1.03 (m, 22H). MS (ESI): *m*/*z* calcd for C_33_H_41_N_2_O_4_ [M + H]^+^, 529.70; found, 529.66.

##### (9*H*-Fluoren-9-yl)methyl 3-Oxo-1,1-dipropyltetrahydro-3*H*-oxazolo[3,4-*a*]pyrazine-7(1*H*)-carboxylate (**26i**)

White solid (166 mg, 37% yield). Eluent for chromatography purification: EtOAc/PEt 3:7; ^1^H NMR (200 MHz, CDCl_3_): δ (ppm) 7.76 (d, *J* = 6.8 Hz, 2H); 7.55 (d, *J* = 7.2 Hz, 2H); 7.44–7.25 (m, 4H); 4.80–4.44 (m, 2H); 4.30–4.00 (m, 3H); 3.67 (d, *J* = 12.0 Hz, 1H); 3.29–3.15 (m, 1H); 3.00–2.56 (m, 3H); 1.61–1.00 (m, 8H); 0.94–0.84 (m, 6H). MS (ESI): *m*/*z* calcd for C_27_H_33_N_2_O_4_ [M + H]^+^, 449.57; found, 449.55.

##### (9*H*-Fluoren-9-yl)methyl 3-Oxo-1,1-diphenyltetrahydro-3*H*-oxazolo[3,4-*a*]pyrazine-7(1*H*)-carboxylate (**26j**)

White solid (305 mg, 59% yield). Eluent for chromatography purification: EtOAc/PEt 1:1; ^1^H NMR (200 MHz, DMSO-*d*_6_): δ (ppm) 7.89 (t, *J* = 7.4 Hz, 2H); 7.59 (d, *J* = 7.4 Hz, 2H); 7.54–7.03 (m, 14H); 4.71–4.38 (m, 2H); 4.26 (t, *J* = 5.4 Hz, 2H); 3.79 (d, *J* = 10.6 Hz, 1H); 3.56 (dd, *J* = 11.4, 1.8 Hz, 2H); 3.00–2.84 (m, 2H); 2.28–2.09 (m, 1H). MS (ESI): *m*/*z* calcd for C_33_H_29_N_2_O_4_ [M + H]^+^, 517.61; found, 517.29.

#### General Procedure for the Synthesis of Final Compounds **3–12**

The appropriate iso(thio)cyanate **27a–b** (2 mmol) and DBU (1.2 mmol) were sequentially added at room temperature to a stirring solution of **26a–j** (1 mmol) in anhydrous THF (5 mL). The reaction solution was continued stirring for 2 h after which it was quenched with a saturated solution of NH_4_Cl (10 mL). The mixture was concentrated under vacuum to half volume, and the aqueous phase was extracted with EtOAc (3 × 15 mL). The organic layers were combined, dried over Na_2_SO_4_, and concentrated under vacuum. The crude products were purified by preparative RP-HPLC.

##### *N*-(4-Fluorobenzyl)-1,1-bis(4-fluorophenyl)-3-oxotetrahydro-1*H*-oxazolo[3,4-*a*]pyrazine-7(3*H*)-carboxamide (**3**)

White solid (289 mg, 60% yield); mp 104–107 °C; ^1^H NMR (400 MHz, DMSO-*d*_6_): δ (ppm) 7.65–7.54 (m, 2H); 7.40–7.19 (m, 9H); 7.18–7.05 (m, 2H); 4.51 (dd, *J* = 11.2, 3.6 Hz, 1H); 4.21 (d, *J* = 5.5 Hz, 2H); 3.93 (d, *J* = 10.5 Hz, 1H); 3.82 (dd, *J* = 13.1, 2.9 Hz, 1H); 3.59 (dd, *J* = 13.2, 2.6 Hz, 1H); 3.05 (td, *J* = 12.8, 3.6 Hz, 1H); 2.68 (td, *J* = 12.9, 3.4 Hz, 1H); 2.17–2.00 (m, 1H). ^13^C NMR (DMSO-*d*_6_): δ 162.94, 162.70, 162.12, 160.49, 160.27, 159.71, 157.05, 154.80, 138.44, 136.75, 134.48, 128.89, 128.09, 127.78, 115.45, 115.24, 114.70, 84.07, 59.51, 45.51, 42.79, 42.52, 41.09. MS (ESI): *m*/*z* calcd for C_26_H_23_F_3_N_3_O_3_ [M + H]^+^, 482.48; found, 482.21. *T*_R_ = 24.77 min.

##### 1,1-Bis(4-chlorophenyl)-*N*-(4-fluorobenzyl)-3-oxotetrahydro-1*H*-oxazolo[3,4-*a*]pyrazine-7(3*H*)-carboxamide (**4**)

White solid (412 mg, 80% yield); mp 77–79 °C; ^1^H NMR (400 MHz, DMSO-*d*_6_): δ (ppm) 7.63–7.55 (m, 2H); 7.53–7.44 (m, 4H); 7.37–7.22 (m, 5H); 7.15–7.07 (m, 2H); 4.52 (dd, *J* = 11.2, 3.6 Hz, 1H); 4.21 (d, *J* = 5.2 Hz, 2H); 3.93 (d, *J* = 11.6 Hz, 1H); 3.83 (dd, *J* = 12.9, 2.9 Hz, 1H); 3.59 (dd, *J* = 13.1, 2.7 Hz, 1H); 3.05 (td, *J* = 12.7, 3.6 Hz, 1H); 2.68 (td, *J* = 13.0, 3.4 Hz, 1H); 2.11 (dd, *J* = 12.8, 11.4 Hz, 1H). ^13^C NMR (DMSO-*d*_6_): δ 162.14, 159.73, 157.07, 154.68, 140.92, 136.96, 136.73, 133.18, 132.84, 128.92, 128.55, 127.77, 127.47, 114.71, 83.94, 59.33, 45.45, 42.81, 42.50, 41.14. MS (ESI): *m*/*z* calcd for C_26_H_23_Cl_2_FN_3_O_3_ [M + H]^+^, 515.39; found, 515.45. *T*_R_ = 22.16 min.

##### *N*-(4-Fluorobenzyl)-3-oxo-1,1-di-*p*-tolyltetrahydro-1*H*-oxazolo[3,4-*a*]pyrazine-7(3*H*)-carboxamide (**5**)

White solid (312 mg, 66% yield); mp 89–90 °C; ^1^H NMR (400 MHz, DMSO-*d*_6_): δ (ppm): 7.41–7.39 (m, 3H); 7.30–7.25 (m, 3H); 7.21–7.09 (m, 7H); 4.43 (dd, *J* = 11.1, 3.7 Hz, 1H); 4.20 (d, *J* = 5.3 Hz, 2H); 3.92 (d, *J* = 11.6 Hz, 1H); 3.84 (dd, *J* = 12.9, 2.9 Hz, 1H); 3.59 (dd, *J* = 13.3, 2.9 Hz, 1H); 3.02 (td, *J* = 12.7, 3.6 Hz, 1H); 2.64 (td, *J* = 12.9, 3.4 Hz, 1H); 2.29 (s, 3H); 2.27 (s, 3H), 2.08–2.02 (m, 1H). ^13^C NMR (DMSO-*d*_6_): δ 162.66, 160.26, 157.63, 155.68, 140.15, 138.08, 137.59, 137.33, 136.28, 129.46, 129.38, 126.18, 125.93, 115.36, 115.15, 85.31, 60.08, 46.32, 43.33, 43.12, 41.52, 21.01. MS (ESI): *m*/*z* calcd for C_28_H_29_FN_3_O_3_ [M + H]^+^, 474.56; found, 474.43. *T*_R_ = 18.53 min.

##### 1,1-Bis(4-(dimethylamino)phenyl)-*N*-(4-fluorobenzyl)-3-oxotetrahydro-1*H*-oxazolo[3,4-*a*]pyrazine-7(3*H*)-carboxamide (**6**)

White solid (500 mg, 94% yield); mp 110–113 °C; ^1^H NMR (400 MHz, DMSO-*d*_6_): δ (ppm) 7.33–7.19 (m, 5H); 7.17–7.06 (m, 2H); 7.01–6.98 (m, 2H); 6.70–6.67 (m, 4H); 4.29 (dd, *J* = 11.2, 3.5 Hz, 1H); 4.22–4.17 (m, 2H); 3.93 (d, *J* = 11.2 Hz, 1H); 3.79 (dd, *J* = 13.0, 2.7 Hz, 1H); 3.59 (dd, *J* = 12.9, 2.7 Hz, 1H); 3.01 (td, *J* = 12.8, 3.4 Hz, 1H); 2.90–2.86 (m, 12H); 2.63 (td, *J* = 13.2, 3.5 Hz, 1H); 2.06 (dd, *J* = 13.0, 11.4 Hz, 1H). ^13^C NMR (DMSO-*d*_6_): δ 162.11, 157.10, 155.50, 149.86, 149.44, 136.81, 129.87, 128.93, 128.85, 126.74, 126.41, 114.82, 114.61, 111.65, 111.54, 85.23, 59.63, 46.00, 42.77, 42.65, 40.84. MS (ESI): *m*/*z* calcd for C_30_H_35_FN_5_O_3_ [M + H]^+^, 532.64; found, 532.41. T_R_ = 11.44 min.

##### *N*-(4-Fluorobenzyl)-3-oxo-1,1-di-*o*-tolyltetrahydro-1*H*-oxazolo[3,4-*a*]pyrazine-7(3*H*)-carboxamide (**7**)

White solid (260 mg,55% yield); mp 217–219 °C; ^1^H NMR (400 MHz, DMSO-*d*_6_): δ (ppm) 7.92–7.81 (m, 1H); 7.68 (d, *J* = 7.5 Hz, 1H); 7.35–7.19 (m, 7H); 7.18–7.05 (m, 4H); 4.95 (dd, *J* = 11.1, 3.7 Hz, 1H); 4.24 (dd, *J* = 15.3, 5.7 Hz, 1H); 4.13 (dd, *J* = 15.1, 5.3 Hz, 1H); 3.88 (d, *J* = 11.4 Hz, 1H); 3.61 (dd, *J* = 13.3, 2.9 Hz, 1H); 3.38–3.22 (m, 2H); 2.81 (td, *J* = 13.3, 3.4 Hz, 1H); 2.19 (dd, *J* = 12.9, 11.2 Hz, 1H); 1.76 (s, 3H); 1.74 (s, 3H). ^13^C NMR (DMSO-*d*_6_): δ 162.12, 159.73, 157.08, 154.97, 137.30, 137.00, 136.80, 136.21, 133.44, 132.71, 131.69, 128.98, 128.90, 128.69, 128.14, 126.74, 126.55, 125.54, 114.80, 114.60, 85.57, 56.28, 45.03, 43.11, 42.81, 41.26, 21.01, 20.45. MS (ESI): *m*/*z* calcd for C_28_H_29_FN_3_O_3_ [M + H]^+^, 474.56; found, 474.34. *T*_R_ = 19.20 min.

##### *N*-(4-Fluorobenzyl)-1,1-bis(2-methoxyphenyl)-3-oxotetrahydro-1*H*-oxazolo[3,4-*a*]pyrazine-7(3*H*)-carboxamide (**8**)

White solid (435 mg, 86% yield); mp 172–173 °C; ^1^H NMR (400 MHz, DMSO-*d*_6_): δ (ppm) 7.45–7.42 (m, 1H); 7.39–7.22 (m, 4H); 7.20–7.15 (m, 1H); 7.14–6.89 (m, 7H); 4.37 (d, *J* = 8.1 Hz, 1H); 4.27–4.22 (m, 1H); 4.16–4.10 (m, 1H); 3.99–3.86 (m, 2H); 3.64 (s, 3H); 3.62 (s, 4H); 3.06 (td, *J* = 12.9, 3.6 Hz, 1H); 2.73 (td, *J* = 13.2, 3.6 Hz, 1H); 2.36–2.27 (m, 1H). ^13^C NMR (DMSO-*d*_6_): δ 162.09, 159.68, 156.90, 156.78, 155.95, 154.76, 136.84, 129.64, 129.56, 128.90, 128.82, 128.20, 127.00, 125.98, 120.18, 119.93, 114.76, 114.55, 112.54, 111.90, 83.94, 59.04, 55.35, 55.29, 44.76, 42.78, 42.62, 40.57. MS (ESI): *m*/*z* calcd for C_28_H_29_FN_3_O_5_ [M + H]^+^, 506.55; found, 506.52. *T*_R_ = 18.71 min.

##### *N*-(4-Fluorobenzyl)-3′-oxo-5′,6′,8′,8a′-tetrahydrospiro[fluorene-9,1′-oxazolo[3,4-*a*]pyrazine]-7′(3′*H*)-carboxamide (**9**)

White solid (421 mg, 95% yield); mp 120–123 °C; ^1^H NMR (400 MHz, DMSO): δ (ppm) 7.91–7.78 (m, 3H); 7.67 (d, *J* = 7.5 Hz, 1H); 7.56–7.47 (m, 2H); 7.42–7.36 (m, 2H); 7.20–7.15 (m, 3H); 7.11–6.95 (m, 2H); 4.19–4.00 (m, 4H); 3.74 (dd, *J* = 12.4, 2.4 Hz, 1H); 3.52 (dd, *J* = 13.0, 2.6 Hz, 1H); 3.08 (td, *J* = 12.3, 3.5 Hz, 1H); 2.88 (td, *J* = 13.1, 3.4 Hz, 1H); 2.70 (dd, *J* = 12.8, 11.4 Hz, 1H). ^13^C NMR (DMSO-*d*_6_): δ 162.08, 156.81, 156.01, 142.93, 140.31, 139.82, 139.63, 136.66, 130.62, 130.52, 128.84, 128.77, 128.61, 128.00, 125.84, 124.86, 120.94, 120.45, 114.77, 114.56, 86.35, 58.93, 44.40, 42.66, 42.21, 41.00. MS (ESI): *m*/*z* calcd for C_26_H_23_FN_3_O_3_ [M + H]^+^, 444.49; found, 444.26. *T*_R_ = 18.91 min.

##### 1,1-Dicyclohexyl-*N*-(4-fluorobenzyl)-3-oxotetrahydro-1*H*-oxazolo[3,4-*a*]pyrazine-7(3*H*)-carboxamide (**10**)

White solid (105 mg, 23% yield); mp 94–95 °C; ^1^H NMR (400 MHz, DMSO-*d*_6_): δ (ppm) 7.37–7.24 (m, 3H); 7.18–7.05 (m, 2H); 4.31–4.16 (m, 2H); 4.06–3.93 (m, 2H); 3.55–3.42 (m, 2H); 2.98–2.72 (m, 3H); 2.00–1.40 (m, 12H); 1.38–0.85 (m, 10H). ^13^C NMR (DMSO-*d*_6_): δ 162.14, 159.74, 157.13, 155.32, 136.82, 128.96, 128.89, 114.82, 114.61, 85.71, 55.26, 43.74, 42.82, 42.18, 40.90, 27.61, 26.54, 26.20, 25.84, 25.77, 25.53, 25.38. MS (ESI): *m*/*z* calcd for C_26_H_37_FN_3_O_3_ [M + H]^+^, 458.60; found, 458.47. *T*_R_ = 21.11 min.

##### *N*-(4-Fluorobenzyl)-3-oxo-1,1-dipropyltetrahydro-1*H*-oxazolo[3,4-*a*]pyrazine-7(3*H*)-carboxamide (**11**)

White solid (189 mg, 50% yield); mp 74–77 °C; ^1^H NMR (400 MHz, DMSO-*d*_6_): δ (ppm) 7.34–7.21 (m, 3H); 7.15–7.10 (m, 2H); 4.23 (d, *J* = 5.5 Hz, 2H); 4.00 (t, *J* = 14.1 Hz, 2H); 3.52 (dd, *J* = 12.7, 2.6 Hz, 1H); 3.42–3.30 (m, 1H); 2.95–2.63 (m, 3H); 1.72–1.43 (m, 4H); 1.42–1.15 (m, 4H); 0.92–0.88 (m, 6H). ^13^C NMR (DMSO-*d*_6_): δ 162.14, 159.73, 156.99, 155.22, 136.86, 128.92, 128.84, 114.84, 114.63, 83.14, 59.10, 44.12, 42.77, 42.58, 33.80, 16.11, 15.88, 14.26, 14.13. MS (ESI): *m*/*z* calcd for C_20_H_29_FN_3_O_3_ [M + H]^+^, 378.47; found, 378.38. *T*_R_ = 13.08 min.

##### *N*-Benzyl-3-oxo-1,1-diphenyltetrahydro-1*H*-oxazolo[3,4-*a*]pyrazine-7(3*H*)-carbothioamide (**12**)

White solid (306 mg, 69% yield); mp 184–186 °C; ^1^H NMR (400 MHz, DMSO-*d*_6_): δ (ppm) 8.50 (s, 1H); 7.73–7.00 (m, 15H); 4.85–4.76 (m, 3H); 4.59–4.54 (m, 2H); 3.64 (d, *J* = 12.6 Hz, 1H); 3.15–2.98 (m, 2H); 2.40 (t, *J* = 12.2 Hz, 1H). ^13^C NMR (DMSO-*d*_6_): δ 182.31, 155.12, 142.22, 139.22, 138.34, 128.54, 128.45, 128.36, 128.07, 127.99, 126.99, 126.58, 125.70, 125.42, 84.66, 59.42, 50.63, 48.53, 46.14, 40.82. MS (ESI): *m*/*z* calcd for C_26_H_26_N_3_O_2_S [M + H]^+^, 444.57; found, 444.23. *T*_R_ = 15.84 min.

##### 1,1-Diphenylhexahydro-3*H*-oxazolo[3,4-*a*]pyrazin-3-one (**28**)

DBU (1.2 mmol) was added to a stirring solution of **26j** (1 mmol) in anhydrous THF (10 mL). The reaction solution was allowed stirring at room temperature for 18 h and monitored by TLC. The solvent was removed under vacuum, and the residue was dissolved in EtOAc (20 mL) and washed with water (20 mL). The organic phase was separated, dried over Na_2_SO_4_, and the solvent was evaporated to give a residue that was purified via flash column chromatography on silica gel using a 1:3 mixture of petroleum ether and EtOAc as the eluent.

Off-white solid (256 mg, 87% yield). ^1^H NMR (200 MHz, DMSO-*d*_6_): δ (ppm) 7.52 (d, *J* = 7.2 Hz, 2H); 7.41–7.24 (m, 8H); 4.32 (d, *J* = 10.1 Hz, 1H); 4.10–3.71 (m, 2H); 3.16 (t, *J* = 5.6 Hz, 1H); 2.97–2.76 (m, 1H); 2.28 (td, *J* = 11.6, 3.4 Hz, 1H); 1.83–1.75 (m, 1H), 1.21 (br s, 1H). MS (ESI): *m*/*z* calcd for C_18_H_19_N_2_O_2_ [M + H]^+^, 295.36; found, 295.38.

#### General Procedure for the Synthesis of Final Compounds **13** and **14**

2-Chloro-*N*-(4-fluorobenzyl)acetamide or 2-chloro-*N*-(4-fluorophenyl)acetamide (1 mmol) was added to a mixture of compound **28** (1 mmol) and K_2_CO_3_ (1.5 mmol) in CH_3_CN (15 mL). The reaction mixture was heated at 90 °C for 4 h after which the solvent was removed under vacuum, and the residue was partitioned between water (15 mL) and CH_2_Cl_2_ (15 mL). The aqueous phase was further extracted with CH_2_Cl_2_ (2 × 15 mL), and the combined organic layers were washed with brine (10 mL) and dried over Na_2_SO_4_. After evaporation, the residue was purified by flash column chromatography on silica gel using a 1:1 mixture of EtOAc/PEt as the eluent.

##### *N*-(4-Fluorophenyl)-2-(3-oxo-1,1-diphenyltetrahydro-3*H*-oxazolo[3,4-*a*]pyrazin-7(1*H*)-yl)acetamide (**13**)

White solid (196 mg, 44% yield); mp 205–208 °C; ^1^H NMR (400 MHz, DMSO-*d*_6_): δ (ppm) 9.76 (s, 1H); 7.62–7.60 (m, 4H); 7.44–7.29 (m, 8H); 7.17–7.13 (m, 2H); 4.77 (dd, *J* = 11.2, 3.6 Hz, 1H); 3.59 (dd, *J* = 13.1, 2.9 Hz, 1H); 3.23–3.13 (m, 3H); 2.74 (d, *J* = 10.5 Hz, 1H); 2.61 (dd, *J* = 13.2, 2.6 Hz, 1H); 2.19 (td, *J* = 12.9, 3.4 Hz, 1H); 1.78 (t, *J* = 12.2 Hz, 1H). ^13^C NMR (DMSO-*d*_6_): δ 167.92, 159.26, 156.88, 155.21, 142.80, 138.58, 134.74, 128.51, 128.36, 128.19, 127.72, 125.53, 125.18, 121.46, 115.24, 115.02, 84.54, 60.85, 60.11, 54.19, 50.66, 41.24. MS (ESI): *m*/*z* calcd for C_26_H_25_FN_3_O_3_ [M + H]^+^, 446.50; found, 446.69. *T*_R_ = 18.30.

##### *N*-(4-Fluorobenzyl)-2-(3-oxo-1,1-diphenyltetrahydro-3*H*-oxazolo[3,4-*a*]pyrazin-7(1*H*)-yl)acetamide (**14**)

White solid (170 mg, 37% yield); mp 137–140 °C; ^1^H NMR (400 MHz, DMSO-*d*_6_): δ (ppm) 8.41–8.37 (m, 1H); 7.56–7.54 (m, 2H); 7.43–7.39 (m, 2H); 7.37–7.25 (m, 8H); 7.17–7.13 (m, 2H); 4.76 (dd, *J* = 10.9, 3.4 Hz, 1H); 4.30–4.22 (m, 2H); 3.62–3.56 (m, 1H); 3.21–3.08 (m, 1H); 3.04 (d, *J* = 13.2 Hz, 1H); 2.88 (d, *J* = 12.9 Hz, 1H); 2.67–2.63 (m, 1H); 2.49–2.44 (m, 1H); 2.29–2.05 (m, 1H); 1.60 (t, *J* = 12.2 Hz, 1H). ^13^C NMR (DMSO-*d*_6_): δ 168.94, 162.24, 155.12, 142.77, 138.49, 135.83, 129.10, 128.51, 128.28, 128.22, 127.72, 125.46, 125.10, 115.38, 101.57, 84.43, 60.51, 59.96, 54.32, 50.97, 41.14. MS (ESI): *m*/*z* calcd for C_27_H_27_FN_3_O_3_ [M + H]^+^, 460.53; found, 460.20. *T*_R_ = 18.16.

#### Method A for the Synthesis of Guanidine Derivatives **15** and **16**

A solution of NaHCO_3_ (1.7 mmol) in H_2_O (1 mL) was added at 0 °C to a stirring solution of **28** (1 mmol) in CH_2_Cl_2_ (5 mL). At the same temperature, a solution of cyanogen bromide (1.2 mmol) in CH_2_Cl_2_ (5 mL) was added. The heterogeneous mixture was vigorously stirred at 0 °C for 30 min, then warmed to room temperature, and stirred for further 24 h. After this time, the layers were separated and the organic phase was washed with a saturated solution of NaHCO_3_ (2 × 10 mL), dried with anhydrous Na_2_SO_4_, and concentrated under vacuum to give a residue from which compound **29** was purified via flash column chromatography on silica gel using a 1:1 mixture of petroleum ether and EtOAc as an eluent.

White solid (160 mg, 50% yield). ^1^H NMR (200 MHz, DMSO-*d*_6_): δ (ppm) 7.52–7.25 (m, 10H), 4.60 (dd, *J* = 11.2, 3.6 Hz, 1H), 3.98–3.88 (m, 1H), 3.37–3.22 (m, 2H), 3.10–3.02 (m, 2H), 1.27–1.18 (m, 1H); MS (ESI): *m*/*z* calcd for C_19_H_18_N_3_O_2_ [M + H]^+^, 320.37; found, 320.40.

Benzylamine or 4-fluorobenzylamine (3 mmol) was added to a stirring solution of **29** (1 mmol) in DMSO (3 mL) in the presence of a catalytic amount of *p*-toluenesulfonic acid. After 18 h of stirring at 60 °C, the reaction solution was diluted with water (10 mL) and extracted with EtOAc (3 × 15 mL). The organic layers were dried over Na_2_SO_4_ and concentrated *in vacuum* after which crude products were purified by flash column chromatography on silica gel using a 4:1 mixture of CH_2_Cl_2_ and MeOH.

#### Method B for the Synthesis of Guanidine Derivatives **15** and **16**

Compounds **15** and **16** were alternatively synthetized according to a manual solid-phase synthesis approach described previously.^18^ Briefly, compounds **31a** or **31b** (0.62 mmol) was added to a suspension of 2-(4-bromomethyl-phenoxy)ethyl polystyrene HL resin (substitution: 1.23 mmol/g, 0.62 mmol) in a 2:1 mixture of CH_2_Cl_2_/DMF (3 mL). The mixture was heated at 50 °C until starting material consumption was observed (4 h). After that, each of the two differently functionalized resins was filtered, washed with DMF (2 × 5 mL) and CH_2_Cl_2_ (2 × 5 mL), and dried. Subsequently, the respective functionalized resin (0.62 mmol) was suspended in CH_3_CN (2 mL) before adding compound **28** (1.55 mmol) and HgCl_2_ (0.93 mmol). After heating at 90 °C for 24 h, a simple filtration was performed and the filtrates were purified by flash column chromatography on silica gel using a 4:1 mixture of CH_2_Cl_2_ and MeOH.

##### *N*-Benzyl-3-oxo-1,1-diphenyltetrahydro-3*H*-oxazolo[3,4-*a*]pyrazine-7(1*H*)-carboximidamide (**15**)

White solid (method A: 56 mg, 21% yield; method B: 103 mg, 39% yield); mp 68–69 °C; ^1^H NMR (400 MHz, DMSO-*d*_6_): δ (ppm) 8.38 (m, 1H), 7.90 (s, 2H), 7.61–7.54 (m, 2H), 7.46–7.25 (m, 14H), 4.77 (dd, *J* = 11.3, 3.5 Hz, 1H), 4.43 (d, *J* = 5.7 Hz, 2H), 3.78–3.58 (m, 2H), 3.23–3.17 (m, 1H), 3.08–3.03 (m, 1H), 2.64–2.57 (m, 1H). ^13^C NMR (DMSO-*d*_6_): δ 156.79, 155.36, 142.90, 138.39, 137.37, 129.02, 128.56, 128.00, 127.57, 126.10, 85.18, 59.76, 49.02, 45.63, 45.56, 41.20. MS (ESI): *m*/*z* calcd for C_26_H_27_N_4_O_2_ [M + H]^+^, 427.53; found, 427.44. T_R_ = 17.68.

##### *N*-(4-Fluorobenzyl)-3-oxo-1,1-diphenyltetrahydro-1*H*-oxazolo[3,4-*a*]pyrazine-7(3*H*)-carboximidamide (**16**)

White solid (method A: 28 mg, 10% yield; method B: 85 mg, 31% yield); mp 82–85 °C; ^1^H NMR (400 MHz, DMSO-*d*_6_): δ (ppm) 8.43 (br s, 1H), 7.95 (s, 2H), 7.60 (d, *J* = 7.4 Hz, 2H), 7.52–7.30 (m, 9H), 7.29–7.09 (m, 3H), 4.84–4.74 (m, 1H), 4.42 (d, *J* = 5.5 Hz, 2H), 3.85–3.53 (m, 3H), 3.23–3.12 (m, 1H), 2.71–2.57 (m, 1H). ^13^C NMR (DMSO-*d*_6_): δ 162.69, 156.14, 154.85, 142.41, 137.86, 133.08, 129.26, 129.18, 128.48, 128.03, 125.54, 115.24, 84.65, 59.26, 54.27, 48.49, 45.03, 44.37, 40.66. MS (ESI): *m*/*z* calcd for C_26_H_26_FN_4_O_2_ [M + H]^+^, 445.52; found, 445.39. *T*_R_ = 18.09.

#### General Procedure for the Synthesis of **36a–e**

Enantiomerically pure monosubstituted piperazines **35a–e** were prepared starting from commercial chiral amino-esters following previously reported procedures, and the analytical data for intermediates **33–35a–e** are in agreement with data from the literature.^19,50^ A solution of di-*tert*-butyl dicarbonate (1.1 mmol) in anhydrous THF was added at 0 °C to a solution of compounds **35a–e** (1 mmol) in dry THF (10 mL). The reaction mixture was warmed at room temperature and further stirred for 1 h. The solvent was removed in vacuo, and the residue was dissolved in CH_2_Cl_2_ (15 mL) and washed with water (3 × 10 mL) and brine (1 × 10 mL). The organic phase was dried over anhydrous Na_2_SO_4_ and concentrated to dryness. Flash column chromatography on silica gel using a 0.5:9.5 mixture of EtOAc/PEt as the eluent provided the desired compounds with good yields.

##### (*S*)-*tert*-Butyl 4-Benzyl-2-methylpiperazine-1-carboxylate (**36a**)

Colorless oil (192 mg, 66% yield). ^1^H NMR (200 MHz, CDCl_3_-*d*): δ (ppm) 7.41–7.20 (m, 5H), 4.28–4.10 (m, 1H), 3.89–3.73 (m, 1H), 3.63–3.33 (m, 2H), 3.23–3.00 (m, 1H), 2.87–2.67 (m, 1H), 2.67–2.50 (m, 1H), 2.23–1.90 (m, 2H), 1.45 (s, 9H), 1.24 (d, *J* = 6.6 Hz, 3H). MS (ESI): *m*/*z* calcd for C_17_H_27_N_2_O_2_ [M + H]^+^, 291.42; found, 291.04.

##### (*S*)-*tert*-Butyl 4-Benzyl-2-isopropylpiperazine-1-carboxylate (**36b**)

Colorless oil (223 mg, 70% yield). ^1^H NMR (200 MHz, DMSO-*d*_6_): δ (ppm) 7.37–7.17 (m, 5H), 3.88–3.73 (m, 1H), 3.60–3.43 (m, 2H), 3.28 (s, 1H), 3.00–2.70 (m, 3H), 2.40–2.20 (m, 1H), 2.00–1.77 (m, 2H), 1.37 (s, 9H), 0.77–0.70 (m, 6H). MS (ESI): *m*/*z* calcd for C_19_H_31_N_2_O_2_ [M + H]^+^, 319.47; found, 319.46.

##### (*S*)-*tert*-Butyl 4-Benzyl-2-isobutylpiperazine-1-carboxylate (**36c**)

Colorless oil (239 mg, 72% yield). ^1^H NMR (200 MHz, DMSO-*d*_6_): δ (ppm) 7.39–7.17 (m, 5H), 4.00 (br s, 1H), 3.83–3.70 (m, 1H), 3.60–3.47 (m, 1H), 3.31 (s, 1H), 3.03–2.85 (m, 1H), 2.80–2.70 (m, 1H), 2.60–2.50 (m, 1H), 2.00–1.82 (m, 2H), 1.60–1.47 (m, 2H), 1.38 (s, 9H), 1.30–1.20 (m, 1H), 0.88–0.80 (m, 6H). MS (ESI): *m*/*z* calcd for C_20_H_33_N_2_O_2_ [M + H]^+^, 333.50; found, 333.07.

##### (*S*)-*tert*-Butyl 2,4-Dibenzylpiperazine-1-carboxylate (**36d**)

Colorless oil (136 mg, 37% yield). ^1^H NMR (200 MHz, DMSO-*d*_6_): δ (ppm) 7.42–7.22, (m, 5H), 7.22–7.10 (m, 3H), 7.03–6.95 (m, 2H), 4.13–4.00 (m, 1H), 3.87–3.72 (m, 1H), 3.63–3.52 (m, 1H), 3.27–3.16 (m, 1H), 3.00–2.73 (m, 4H), 2.60 (m, 1H), 2.10–1.83 (m, 2H), 1.26 (s, 9H). MS (ESI): *m*/*z* calcd for C_23_H_31_N_2_O_2_: [M + H]^+^, 367.51; found, 367.02.

##### (*S*)-*tert*-Butyl 4-Benzyl-2-phenylpiperazine-1-carboxylate (**36e**)

Colorless oil (229 mg, 65% yield). ^1^H NMR (200 MHz, DMSO-*d*_6_): δ (ppm) 7.34–7.18 (m, 10 H), 5.17–5.06 (m, 1H), 3.90–3.73 (m, 1H), 3.60–3.44 (m, 2H), 3.30–3.20 (m, 1H), 3.00–2.83 (m, 1H), 2.83–2.70 (m, 1H), 2.37–2.25 (m, 1H), 2.13–1.93 (m, 1H), 1.39 (s, 9H). MS (ESI): *m*/*z* calcd for C_22_H_29_N_2_O_2_ [M + H]^+^, 353.49; found, 353.45.

#### General Procedure for the Synthesis of **37a–e**

TMEDA (2.7 mmol) was added under an argon atmosphere at room temperature to a stirring solution of **36a-e** (1 mmol) in freshly distilled THF (5 mL). After cooling at −78 °C, *sec*-BuLi (2.7 mmol) was added, and the reaction solution was allowed to reach −30 °C over 2 h. A solution of benzophenone (2 mmol) in THF (5 mL) was added, and the reaction mixture was left stirring at −30 °C for 30 min, then slowly warmed to room temperature, and stirred for 18 h. The reaction was quenched with a saturated solution of NH_4_Cl (15 mL), and the solvents were concentrated under vacuum to half volume giving a residue, which was extracted with EtOAc (3 × 15 mL). The organic layers were combined, dried over Na_2_SO_4_, and the solvent was removed under vacuum. The resulting crude product was purified by flash column chromatography on silica gel using a 1:9 mixture of EtOAc/PEt as the eluent.

##### (5*S*,8a*R*)-7-Benzyl-5-methyl-1,1-diphenylhexahydro-3*H*-oxazolo[3,4-*a*]pyrazin-3-one (**37a**)

Yellow oil (100 mg, 25% yield). ^1^H NMR (200 MHz, CDCl_3_): δ (ppm) 7.56–7.44 (m, 2H), 7.44–7.20 (m, 13H), 4.70 (d, *J* = 9.1 Hz, 1H), 4.15–4.01 (m, 1H), 3.60–3.45 (m, 1H), 3.33–3.22 (m, 1H), 2.63–2.47 (m, 2H), 2.11–1.97 (m, 1H), 1.62–1.50 (m, 2H), 1.39–1.29 (m, 2H). MS (ESI): *m*/*z* calcd for C_26_H_27_N_2_O_2_ [M + H]^+^, 399.51; found, 399.44.

##### (5*S*,8a*R*)-7-Benzyl-5-isopropyl-1,1-diphenylhexahydro-3*H*-oxazolo[3,4-*a*]pyrazin-3-one (**37b**)

Colorless oil (77 mg, 18% yield). ^1^H NMR (200 MHz, CDCl_3_): δ (ppm) 7.70–7.60 (m, 2H), 7.60–7.14 (m, 13H), 4.73–4.40 (m, 1H), 3.70–3.03 (m, 3H), 2.91–2.70 (m, 1H), 2.61–2.47 (m, 1H), 2.34–2.18 (m, 1H), 1.94–1.91 (m, 1H), 1.27–1.22 (m, 1H), 0.79 (s, 3H), 0.78 (s, 3H). MS (ESI): *m*/*z* calcd for C_28_H_31_N_2_O_2_ [M + H]^+^, 427.57; found, 427.48.

##### (5*S*,8a*R*)-7-Benzyl-5-isobutyl-1,1-diphenylhexahydro-3*H*-oxazolo[3,4-*a*]pyrazin-3-one (**37c**)

Yellow oil (79 mg, 18% yield). ^1^H NMR (200 MHz, CDCl_3_): δ (ppm) 7.57–7.47 (m, 2H), 7.43–7.29 (m, 13H), 4.76–4.64 (m, 1H), 4.06–3.86 (m, 1H), 3.56–3.47 (m, 1H), 3.27–3.16 (m, 1H), 2.69–2.25 (m, 2H), 2.11–1.98 (m, 1H), 1.65–1.63 (m, 2H), 1.03–0.89 (m, 2H), 0.86 (dd, *J* = 6.5, 3.7 Hz, 6H). MS (ESI): *m*/*z* calcd for C_29_H_33_N_2_O_2_ [M + H]^+^, 441.60; found, 441.46.

##### (5*S*,8a*R*)-5,7-Dibenzyl-1,1-diphenylhexahydro-3*H*-oxazolo[3,4-*a*]pyrazin-3-one (**37d**)

Yellow oil (247 mg, 52% yield). ^1^H NMR (200 MHz, CDCl_3_): δ (ppm) 7.46–7.44 (m, 2H), 7.39–7.27 (m, 13H), 7.15–7.08 (m, 3H), 7.01–6.94 (m, 2H), 4.77 (dd, *J* = 11.1, 3.3 Hz, 1H), 4.19–4.07 (m, 1H), 3.54 (d, *J* = 12.9 Hz, 1H), 3.23 (d, *J* = 12.9 Hz, 1H), 3.12–2.98 (m, 2H), 2.63 (d, *J* = 11.5 Hz, 2H), 1.95 (dd, *J* = 11.7, 3.9 Hz, 1H), 1.73–1.60 (m, 1H). MS (ESI): *m*/*z* calcd for C_32_H_31_N_2_O_2_ [M + H]^+^, 475.61; found, 475.35.

##### (5*S*,8a*R*)-7-Benzyl-1,1,5-triphenylhexahydro-3*H*-oxazolo[3,4-*a*]pyrazin-3-one (**37e**)

C_31_H_28_N_2_O_2_ (88 mg, 19% yield); ^1^H NMR (400 MHz, CDCl_3_): δ (ppm) 7.61–7.58 (m, 2H), 7.37–7.28 (m, 6H), 7.24–6.88 (m, 11H), 3.84–3.75 (m, 1H), 3.55–3.38 (m, 2H), 3.32–3.29 (m, 2H), 2.96–2.80 (m, 1H), 2.64–2.47 (m, 1H), 2.35–2.20 (m, 1H), 1.36–1.22 (m, 1H). MS (ESI): *m*/*z* calcd for C_31_H_29_N_2_O_2_ [M + H]^+^, 461.59; found, 461.49.

#### General Procedure for the Synthesis of **38a–e**

9-Fluorenylmethoxycarbonyl chloride (FmocCl, 1.1 mmol) was added to a solution of **37a–e** (1 mmol) in CH_3_CN (5 mL). The reaction solution was heated at 90 °C for 5 h and then stirred at room temperature for 18 h. The solvent was evaporated giving a residue that was dissolved in EtOAc (15 mL), and the resulting organic phase was washed with water (3 × 10 mL), dried over anhydrous Na_2_SO_4_, and concentrated to dryness. All crude residues were finally purified via flash column chromatography on silica gel using a 1:4 mixture of EtOAc/PEt as the eluent.

##### (9*H*-Fluoren-9-yl)methyl (5*S*,8a*R*)-5-Methyl-3-oxo-1,1-diphenyltetrahydro-3*H*-oxazolo[3,4-*a*]pyrazine-7(1*H*)-carboxylate (**38a**)

White solid (440 mg, 83% yield); ^1^H NMR (400 MHz, DMSO): δ (ppm) 7.97–7.84 (m, 3H), 7.47–7.22 (m, 13H), 7.06–6.93 (m, 2H), 5.02–4.91 (m, 1H), 4.51–4.33 (m, 2H) 4.33–4.21 (m, 1H), 4.13–4.05 (m, 1H), 3.11–3.00 (m, 1H), 3.00–2.87 (m, 1H), 2.87–2.75 (m, 1H), 2.33–2.11 (m, 1H), 0.87–0.79 (d, *J* = 7.2 Hz, 3H); ^13^C NMR (400 MHz, DMSO): δ 154.61, 144.05, 141.94, 140.67, 137.74, 128.64, 128.46, 127.86, 127.60, 127.54, 127.07, 127.00, 125.43, 124.95, 120.02, 84.15, 64.96, 55.01, 47.27, 46.36, 45.96, 45.02, 24.02. MS (ESI): *m*/*z* calcd for C_34_H_31_N_2_O_4_ [M + H]^+^, 531.63; found, 531.52.

##### (9*H*-Fluoren-9-yl)methyl (5*S*,8a*R*)-5-Isopropyl-3-oxo-1,1-diphenyltetrahydro-3*H*-oxazolo[3,4-*a*]pyrazine-7(1*H*)-carboxylate (**38b**)

White solid (318 mg, 57% yield); ^1^H NMR (400 MHz, CDCl_3_): δ (ppm): 7.87–7.73 (m, 2H), 7.64–7.16 (m, 14H), 7.07–6.96 (m, 2H), 5.29–5.20 (dd, 1H), 4.56–4.40 (m, 1H), 4.24–4.11 (m, 2H), 3.40–3.16 (m, 1H), 2.80–2.64 (m, 1H), 2.26–2.01 (m, 1H), 1.81–1.56 (m, 2H), 1.16–1.04 (m, 1H), 0.94 (d, *J* = 6.4 Hz, 3H), 0.62 (d, *J* = 6.8 Hz, 3H); ^13^C NMR (400 MHz, CDCl_3_): δ 155.96, 144.29, 143.23, 142.11, 141.41, 138.79, 138.02, 128.61, 127.96, 127.85, 127.18, 125.64, 125.34, 124.56, 120.00, 119.63, 84.87, 64.89, 57.60, 56.77, 48.27, 46.09, 43.89, 25.64, 19.98, 19.47. MS (ESI): *m*/*z* calcd for C_36_H_35_N_2_O_4_ [M + H]^+^, 559.69; found, 559.57.

##### (9*H*-Fluoren-9-yl)methyl (5*S*,8a*R*)-5-Isobutyl-3-oxo-1,1-diphenyltetrahydro-3*H*-oxazolo[3,4-*a*]pyrazine-7(1*H*)-carboxylate (**38c**)

White solid (309 mg, 54% yield). ^1^H NMR (400 MHz, CDCl_3_): δ (ppm): 7.78–7.75 (m, 2H), 7.64–7.16 (m, 14H), 7.07–6.96 (m, 2H), 5.29–5.20 (m, 1H), 4.56–4.40 (m, 1H), 4.24–4.11 (m, 2H), 3.40–3.16 (m, 1H), 2.80–2.64 (m, 1H), 2.26–2.01 (m, 1H), 1.81–1.56 (m, 2H), 1.40–1.23 (m, 2H), 1.16–1.04 (m, 1H), 0.98–0.91 (d, *J* = 6.4 Hz, 3H), 0.67–0.60 (d, *J* = 6.8 Hz, 3H). MS (ESI): *m*/*z* calcd for C_37_H_37_N_2_O_4_ [M + H]^+^, 573.71; found, 573.73.

##### (9*H*-Fluoren-9-yl)methyl (5*S*,8a*R*)-5-Benzyl-3-oxo-1,1-diphenyltetrahydro-3*H*-oxazolo[3,4-*a*]pyrazine-7(1*H*)-carboxylate (**38d**)

White solid (376 mg, 62% yield). ^1^H NMR (400 MHz, CDCl_3_): δ (ppm) 7.84–7.71 (m, 2H), 7.71–7.01 (m, 21H), 5.22–5.11 (m, 1H), 4.63–4.47 (m, 2H), 4.26–4.15 (m, 1H), 4.13–3.89 (m, 2H), 3.42–3.31 (m, 1H), 2.89–2.82 (m, 1H), 2.82–2.61 (m, 1H), 2.61–2.44 (m, 1H), 2.27–2.00 (m, 1H). MS (ESI): *m*/*z* calcd for C_40_H_35_N_2_O_4_ [M + H]^+^, 607.73; found, 607.49.

##### (9*H*-Fluoren-9-yl)methyl (5*S*,8a*R*)-3-Oxo-1,1,5-triphenyltetrahydro-3*H*-oxazolo[3,4-*a*]pyrazine-7(1*H*)-carboxylate (**38e**)

White solid (350 mg, 59% yield); ^1^H NMR (400 MHz, CDCl_3_): δ (ppm) 7.95–6.88 (m, 23H), 5.10–4.98 (m, 1H), 4.68–4.52 (m, 1H), 4.40–4.30 (m, 1H), 4.30–4.18 (m, 1H), 4.18–4.09 (m, 1H), 3.83–3.78 (m, 1H), 3.78–3.67 (m, 1H), 2.93–2.73 (m, 2H). MS (ESI): *m*/*z* calcd for C_39_H_33_N_2_O_4_ [M + H]^+^, 593.70; found, 593.50.

#### General Procedure for the Synthesis of Final Compounds **17–21**

4-Fluorobenzyl isocyanate (2 mmol) and DBU (1.2 mmol) were sequentially added at room temperature to a stirring solution of **38a–e** (1 mmol) in anhydrous THF (5 mL). The reaction solution was stirred for 2 h and then it was quenched with a saturated solution of NH_4_Cl (10 mL). The mixture was concentrated under vacuum to half volume and the aqueous phase was extracted with EtOAc (3 × 15 mL). The organic layers were combined, dried over Na_2_SO_4_, and concentrated in vacuum. The desired products were purified by preparative RP-HPLC.

##### (5*S*,8a*R*)-*N*-(4-Fluorobenzyl)-5-methyl-3-oxo-1,1-diphenyltetrahydro-1*H*-oxazolo[3,4-*a*]pyrazine-7(3*H*)-carboxamide (**17**)

White solid (225 mg, 49% yield); mp 153–156 °C; ^1^H NMR (400 MHz, DMSO-*d*_6_): δ (ppm) 7.57–7.55 (m, 2H), 7.44–7.25 (m, 11H), 7.14–7.09 (m, 2H), 4.70 (dd, *J* = 11.3, 3.8 Hz, 1H), 4.28–4.14 (m, 2H), 3.98–3.84 (m, 2H), 3.79 (d, *J* = 13.7 Hz, 1H), 2.87 (dd, *J* = 13.7, 3.9 Hz, 1H), 2.15–2.01 (m, 1H), 1.15 (d, J = 6.9 Hz, 3H). ^13^C NMR (DMSO-*d*_6_): δ 162.12, 157.39, 154.69, 142.36, 138.46, 136.92, 128.83, 128.75, 128.55, 128.40, 128.24, 127.84, 125.61, 125.35, 114.83, 114.62, 84.82, 55.76, 46.83, 46.11, 45.45, 42.80, 15.33. MS (ESI): *m*/*z* calcd for C_26_H_27_FN_3_O_3_ [M + H]^+^, 460.53; found, 460.49. *T*_R_ = 23.10. [α]_D_^22^ = +1059.13 (*c* 0.023).

##### (5*S*,8a*R*)-*N*-(4-Fluorobenzyl)-5-isopropyl-3-oxo-1,1-diphenyltetrahydro-1*H*-oxazolo[3,4-*a*]pyrazine-7(3*H*)-carboxamide (**18**)

White solid (229 mg, 47% yield); mp 186–187 °C; ^1^H NMR (400 MHz, DMSO-*d*_6_): δ (ppm) 7.65 (d, *J* = 7.5 Hz, 2H); 7.43–7.38 (m, 6H); 7.35–7.28 (m, 2H); 7.28–7.20 (m, 3H); 7.13–7.08 (m, 2H); 4.67 (dd, *J* = 11.2, 3.9 Hz, 1H); 4.19 (dd, *J* = 15.1, 5.4 Hz, 2H); 4.01 (d, *J* = 14.2 Hz, 1H); 3.71 (dd, *J* = 12.8, 3.6 Hz, 1H); 3.26 (dd, *J* = 12.3, 3.8 Hz, 1H); 2.88 (dd, *J* = 13.8, 3.9 Hz, 1H); 2.36–2.21 (m, 1H); 1.93–1.76 (m, 1H); 0.92 (d, *J* = 6.6 Hz, 3H); 0.60 (d, *J* = 6.6 Hz, 3H). ^13^C NMR (DMSO-*d*_6_): δ 162.11, 159.71, 157.18, 155.27, 142.90, 138.34, 136.94, 128.82, 128.75, 128.50, 128.43, 128.15, 127.79, 125.25, 125.17, 114.80, 114.59, 84.77, 57.01, 56.58, 45.15, 43.05, 42.82, 26.10, 19.47, 18.98. MS (ESI): *m*/*z* calcd for C_29_H_31_FN_3_O_3_ [M + H]^+^, 488.58; found, 488.54. *T*_R_ = 26.07. [α]_D_^22^ = +1058.5 (*c* 0.012).

##### (5*S*,8a*R*)-*N*-(4-Fluorobenzyl)-5-isobutyl-3-oxo-1,1-diphenyltetrahydro-1*H*-oxazolo[3,4-*a*]pyrazine-7(3*H*)-carboxamide (**19**)

White solid (100 mg, 20% yield); mp 216–219 °C; ^1^H NMR (400 MHz, DMSO-*d*_6_): δ (ppm) 7.61–7.55 (m, 2H), 7.42–7.07 (m, 13H), 4.69 (dd, *J* = 11.1, 3.6 Hz, 1H), 4.29–4.13 (m, 3H), 3.85–3.75 (m, 2H), 2.91 (dd, *J* = 13.6, 3.9, 1H), 2.17 (m, 1H), 1.63–1.52 (m, 1H), 1.40–1.23 (m, 2H), 0.80 (d, *J* = 6.4, 3H), 0.76 (d, *J* = 6.8, 3H). ^13^C NMR (DMSO-*d*_6_): δ 162.12, 157.29, 155.77, 155.01, 142.58, 138.36, 136.94, 128.96, 128.85, 128.78, 128.44, 128.19, 127.79, 125.42, 125.28, 115.13, 114.96, 114.75, 114.57, 84.79, 55.89, 48.69, 45.99, 45.16, 42.75, 37.37, 24.13, 22.64, 21.65. MS (ESI): *m*/*z* calcd for C_30_H_33_FN_3_O_3_ [M + H]^+^, 502.61; found, 502.40. *T*_R_ = 26.28. [α]_D_^22^ = + 794.29 (*c* 0.022).

##### (5*S*,8a*R*)-*N*-(4-Fluorobenzyl)-5-benzyl-3-oxo-1,1-diphenyltetrahydro-1*H*-oxazolo[3,4-*a*]pyrazine-7(3*H*)-carboxamide (**20**)

White solid (134 mg, 25% yield); mp 95–98 °C; ^1^H NMR (400 MHz, DMSO-*d*_6_): δ (ppm) 7.51–7.48 (m, 2H); 7.43–7.25 (m, 11H); 7.16–7.04 (m, 7H); 4.87 (dd, *J* = 11.2, 3.7 Hz, 1H); 4.29–4.18 (m, 2H); 4.03–3.99 (m, 1H); 3.94–3.88 (m, 1H); 3.82 (dd, *J* = 13.1, 3.2 Hz, 1H); 2.99–2.79 (m, 3H); 2.26–2.14 (m, 1H). ^13^C NMR (DMSO-*d*_6_): δ 162.12, 159.73, 157.43, 154.95, 142.44, 138.22, 137.71, 136.86, 128.96, 128.85, 128.37, 128.29, 128.05, 127.93, 127.79, 126.05, 125.57, 125.33, 114.82, 114.61, 84.76, 56.42, 52.19, 45.66, 45.35, 42.87, 34.85. MS (ESI): *m*/*z* calcd for C_33_H_31_FN_3_O_3_ [M + H]^+^, 536.63; found, 536.38. *T*_R_ = 26.10. [α]_D_^23^ = +539.8 (*c* 0.024).

##### (5*S*,8a*R*)-*N*-(4-Fluorobenzyl)-3-oxo-1,1,5-triphenyltetrahydro-1*H*-oxazolo[3,4-*a*]pyrazine-7(3*H*)-carboxamide (**21**)

White solid (83 mg, 16% yield); mp 105–107 °C; ^1^H NMR (400 MHz, DMSO-*d*_6_): δ (ppm) 7.63 (d, *J* = 7.9 Hz, 2H); 7.50 (t, *J* = 7.7 Hz, 2H); 7.39 (t, *J* = 7.1 Hz, 1H); 7.24 (t, *J* = 6.9 Hz, 2H); 7.15–6.99 (m, 11H); 6.97–6.88 (m, 2H); 5.03 (d, *J* = 13.9 Hz, 1H); 4.17–4.06 (m, 2H); 3.75–3.62 (m, 2H); 2.85–2.80 (m, 1H), 2.71–2.55 (m, 2H). ^13^C NMR (DMSO-*d*_6_): δ 162.04, 159.64, 155.95, 139.25, 138.42, 136.73, 134.11, 128.60, 128.01, 127.42, 125.92, 125.61, 114.69, 114.47, 87.97, 71.18, 47.37, 42.64, 42.11. MS (ESI): *m*/*z* calcd for C_32_H_29_FN_3_O_3_ [M + H]^+^, 522.60; found, 522.46. *T*_R_ = 26.72. [α]_D_^23^ = +8.8 (*c* 0.025).

### Pharmacology

#### Calcium Mobilization Assay

HEK293_mNPSR_ cells were generated as previously described^3^ and maintained in DMEM supplemented with 10% fetal bovine serum, 2 mM l-glutamine, and hygromycin B (100 mg/L) and cultured at 37 °C in 5% CO_2_ humidified air. HEK293_mNPSR_ cells were seeded at a density of 50,000 cells/well into poly-d-lysine coated 96-well black, clear-bottom plates. The following day, the cells were incubated with medium supplemented with 2.5 mM probenecid, 3 μM of the calcium sensitive fluorescent dye Fluo-4 AM, and 0.01% pluronic acid, for 30 min at 37 °C. After that time, the loading solution was aspirated and 100 μL/well of assay buffer (Hanks’ balanced salt solution; HBSS) supplemented with 20 mM 4-(2-hydroxyethyl)-1-piperazineethanesulfonic acid (HEPES), 2.5 mM probenecid, and 500 μM Brilliant Black (Sigma-Aldrich) was added. Concentrated solution (1 mM) of NPS was made in bidistilled water and kept at −20 °C. Concentrated solutions (10 mM) of NPSR antagonists were made in DMSO and kept at −20 °C. Serial dilutions were carried out in HBSS/HEPES (20 mM) buffer (containing 0.02% bovine serum albumin fraction V). After placing both plates (cell culture and master plate) into the fluorometric imaging plate reader FlexStation II (Molecular Devices, Sunnyvale, CA), fluorescence changes were measured. On-line additions were carried out in a volume of 50 μL/well. To facilitate drug diffusion into the wells in antagonist type experiments, the present studies were performed at 37 °C, and three cycles of mixing (25 μL from each well moved up and down 3 times) were performed immediately after antagonist injection to the wells. Inhibition response curves were determined against the stimulatory effect of 10 nM NPS. Additionally, the concentration–response curve to NPS has been tested in the absence and in the presence of 100 nM of **1**, compound **16**, and compound **21** (Figure 3). NPSR antagonists were injected into the wells 24 min before adding NPS.

#### Mouse Locomotor Activity Test

All animal care and experimental procedures conformed with the European Communities Council Directives (2010/63/EU) and national regulations (D.L. 26/2014). Studies involving animals are reported in accordance with the ARRIVE guidelines.^51^ This study was approved by the Italian Ministry of Health (authorization number 120/2014-PR). The experiments were performed with CD-1 mice (2–4 month old, from the Laboratory for Preclinical Research (LARP) of the University of Ferrara, Italy). Mice were housed under standard conditions (22 °C, 55% humidity, 12 h light/dark cycle, light on at 7:00 am), with free access to food and water. Appropriate environmental enrichment was present in each cage. Mice were killed with CO_2_ overdose. Each animal was used only once. Experiments were performed during the light cycle (between 09.00 and 13.00) according to Guerrini et al. (2009).^42^ For *in vivo* studies, **1**, **16,** and **21** were solubilized in water containing 1% DMSO and 10% Cremophor EL (Sigma-Aldrich). NPS was solubilized in saline solution. Vehicle, **1**, **16**, and **21** were injected by the ip route 30 min before saline or NPS injection. NPS or saline were given by the i.c.v. route 15 min before the beginning of the test and the locomotor activity was recorded for 30 min. The i.c.v. injections (2 μL/mouse) were given under light (just enough to produce loss of the righting reflex) isofluorane anesthesia into the left ventricle according to the procedure described by Laursen and Belknap (1986)^52^ and routinely adopted in our laboratory. For these experiments, the ANY-maze video tracking system was used (Ugo Basile, application version 4.52c Beta). Mice were positioned in a square plastic cage (40 × 40 cm), one mouse per cage. Four mice were monitored in parallel. Mouse horizontal activity was monitored by a camera while vertical activity was measured by an infrared beam array. The parameters measured were cumulative distance traveled (total distance in m that the animal traveled during the test), immobility time (the animal is considered immobile when 90% of it remains in the same place for a minimum of 2.5 s), and the number of rearings (the number of beam breaks due to the vertical movements; this input is triggered only when the beam is interrupted for at least 200 ms). Previous studies performed under the present experimental conditions demonstrated that NPS stimulated locomotor activity in a dose-dependent way;^42^ from these studies, the dose of 0.1 nmol was selected as the lower dose inducing statistically significant effects.

#### Data Analysis and Terminology

Data are expressed as means ± standard error of the mean (SEM) of n experiments. Nonlinear regression analysis using GraphPad Prism software (v.4.0) allowed logistic iterative fitting of the resultant responses and the calculation of agonist potencies and maximal effects. Agonist potency was expressed as pEC_50_, which is the negative logarithm to base 10 of the agonist molar concentration that produces 50% of the maximal possible effect of that agonist. In inhibition response experiments (i.e., increasing concentrations of antagonist vs a fixed concentration of agonist), the antagonist potency was expressed as p*K*_B_, derived from the following equation
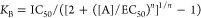
where IC_50_ is the concentration of antagonist that produces 50% inhibition of the agonist response, [A] is the concentration of agonist, EC_50_ is the concentration of agonist producing a 50% maximal response, and *n* is the Hill coefficient of the concentration–response curve to the agonist. When the concentration–response curve to NPS has been tested in the absence and in the presence of antagonists, the antagonist potency was expressed as p*A*_2_, derived from the following equation
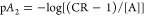
where CR means the ratio between agonist potency in the presence and absence of antagonist and [A] the molar concentration of the antagonist.

*In vivo* data are expressed as mean ± SEM of n animals. Data were analyzed using two-way ANOVA, followed by Bonferroni’s post-hoc test. Differences were considered statistically significant when *p* < 0.05.

### Molecular Modeling

#### hNPSR Model Construction

The query sequence of the hNPSR in its I107 variant (Q6W5P4, FASTA format) was downloaded from the Universal Protein Resource.^53^ BLAST (Basic Local Alignment Search Tool) was used to search the homologous sequences to be used as template structures. The sequences of templates were obtained from the Uniprot web server. The human C5a anaphylatoxin chemotactic receptor 1 (P21730, PDB 6C1R),^54^ the human κ opioid receptor (P41145, PDB 4DJH),^33^ the human M2 muscarinic receptor (P08172, PDB 5ZKC),^55^ the human neuropeptide Y Y1 Receptor (Q15761, PDB 5ZBH),^29^ the human orexin-1 receptor (O43613, PDB 6TOD),^56^ and the human type-2 angiotensin receptor (P50052, PDB 4ZUD)^38^ were chosen as the templates. The sequence identity and coverage between hNPSR and the six templates are reported in Table S1 along with the phylogenetic tree (Figure S3) and pairwise sequence alignments (Figures S4–S9). The choice of templates was dictated by the resolution and sufficient similarity sequence coverage with the target. The alignments reported in Figures S4–S9 were used to build six hNPSR models using the Prime module within Schrodinger (Prime; Schrödinger, LLC, version 2020-1). The X-ray structures of the templates were obtained from the Protein Data Bank. Homology built models were obtained using the knowledge-based method. Validation of the model was carried by generating the Ramachandran plots for each model. This analysis allowed supporting the viability of the constructed models that all had >90% of the residues in the allowed regions of the plot.

#### Docking Calculations

Docking calculations were attained employing the Glide tool implemented in Maestro 12.4.^57^ The 3D structures of **1**, **3–5**, **7**, **10**, **12**, and **14–21** were generated with the Maestro fragment Build tool and then energetically minimized with Macromodel.^58^ The 6 models were all prepared through the Protein Preparation Wizard of the Maestro 12.4 graphical user interface, which assigns bond orders, adds hydrogen atoms, and generates appropriate protonation states.

The docking grid box was centered on the residues lining the putative binding orthosteric binding, with a grid box dimension equal to 31 Å × 31 Å × 31 Å. Finally, docking runs were carried out using the standard precision method. Pictures were rendered employing UCSF Chimera software.^59^

#### MD Simulation System Setup

The complexes obtained from docking experiments of **1**, **16**, and **21** in both BM1 and BM2 employing the hNPSR model built using the human neuropeptide Y Y1 receptor (hNPY1R, PDB code 5ZBH) structure as a template were used to build an MD simulation system. The complex was embedded in a membrane of phosphatidylcholine lipids^60^ using Maestro’s system builder and was located in the membrane using the default parameters. Next, the system was solvated in an orthorhombic water box with a buffer distance of 10 Å.^61^ The system was neutralized with 9 Cl^–^ ions for the complexes with **1** and **21** while 10 Cl^–^ ions were included in the **16**/hNPSR complexes. The salt concentration was set to 0.15 M NaCl. The OPLS3 force field was used for the constructed receptor/ligand/membrane system.^62^

#### MD Simulation Protocols

The Desmond module within the Schrödinger suite was used for MD simulations.^63−65^ First, the system was relaxed using the following relaxation protocol for membrane proteins:

1. minimization with restraints on all solute-heavy atoms;
2 unrestrained minimization;
3 Brownian Dynamics in NVT ensemble (constant number of particles, constant volume, constant temperature of 300 K) using small timesteps and restraints on solute heavy atoms (50 kcal/mol) at 10 K for 50 ps;
4 *NPT* ensemble simulation (constant number of particles, constant pressure of 1 bar, and constant temperature of 100 K) Gaussian barrier potential on water molecules, membrane restrained in *z*, protein restrained (20 kcal/mol) for 20 ps;
5 *NP*γ*T* ensemble simulation (constant number of particles, constant pressure of 1 bar, constant temperature of 100 K, and lateral surface tension of membranes) Gaussian Barrier potential on water molecules, membrane restrained in *z*, protein restrained (10 kcal/mol) for 100 ps;
6 *NP*γ*T* ensemble simulation with increasing temperature from 100 to 300 K, Gaussian Barrier potential on water molecules, and gradual release of restraints for 150 ps.
7 *NVT* production run (constant number of particles, constant volume, and constant temperature of 300 K) without any restraint for 100 ps.

After the relaxation, 100 ns production runs were conducted under the *NP*γ*T* ensemble for each of the six systems using the default protocol. In detail, the temperature was controlled using the Nosé–Hoover thermostat^66,67^ with a coupling constant of 1.0 ps. The pressure was controlled using the Martyna–Tuckerman–Klein barostat^68,69^ with a coupling constant of 2.0 ps. The cutoff distance for short-range nonbonded interactions was 9 Å, and the long-range van der Waals interactions were based on a uniform density approximation. To minimize the computation time, nonbonded forces were calculated using an r-RESPA integrator^70^ where the short-range forces were updated every two steps and the long-range forces were updated every six steps. The trajectories were saved at 100.0 ps. The Desmond SID tool was used to analyze the receptor–ligand interactions during the MD trajectory. Particular attention was given to ligand–residue interactions and ligand RMSF.

## Supporting Information Available

^1^H NMR and NOE NMR analyses of compound **17**; sequence identity and coverage between the hNPSR and selected hGPCRs; phylogenetic tree of the hNPSR and the six selected template structures; pairwise sequence alignments of the hNPSR and the six selected template structures; ligand-root-mean-square fluctuation of compounds **1**, **16**, and **21**; and ligand–NPSR interactions for compounds **1**, **16**, and **21** (PDF)
Molecular formula strings (CSV)
3D coordinates of the energy-minimized docked pose of **1** in the constructed model of NPSR during MD simulation in BM1 (PDB)
3D coordinates of the energy-minimized docked pose of **1** in the constructed model of NPSR during MD simulation in BM2 (PDB)
3D coordinates of the energy-minimized docked pose of **16** in the constructed model of NPSR during MD simulation in BM1 (PDB)
3D coordinates of the energy-minimized docked pose of **16** in the constructed model of NPSR during MD simulation in BM2 (PDB)
3D coordinates of the energy-minimized docked pose of **21** in the constructed model of NPSR during MD simulation in BM1 (PDB)
3D coordinates of the energy-minimized docked pose of **21** in the constructed model of NPSR during MD simulation in BM2 (PDB)

## Supplementary Material

jm0c02223_si_002.csv

jm0c02223_si_003.pdb

jm0c02223_si_004.pdb

jm0c02223_si_005.pdb

jm0c02223_si_006.pdb

jm0c02223_si_007.pdb

jm0c02223_si_008.pdb

## Abbreviations

BLAST: Basic Local Alignment Search Tool
BM: binding mode
Boc_2_O: di-*tert*-butyl dicarbonate
CL_95%_: 95% confidence limit
CCR9: CC chemokine receptor type 9
CXCR4: chemokine receptor type 4
DMEM: Dulbecco’s modified Eagle’s medium
ECL: extracellular loop
EtOAc: ethyl acetate
FmocCl: Fmoc chloride
HBSS: Hanks’ balanced salt solution
HEPES: 4-(2-hydroxyethyl)-1-piperazineethanesulfonic acid
hNPY1R: human neuropeptide Y Y1 receptor
NPS: neuropeptide S
NPSR: neuropeptide S receptor
NTSR1: neurotensin receptor 1
OX1R: orexin receptor type 1
PAR1: protease-activated receptor 1
PEt: petroleum ether
*p*-TsOH: *p*-toluenesulfonic acid
RMSF: root-mean-square fluctuation
*sec*-BuLi: *sec*-butyllithium
SEM: standard error of the mean
TM: transmembrane
