# Supplementary material for: Structure–Activity Relationship Studies on Oxazolo[3,4-a]pyrazine Derivatives Leading to the Discovery of a Novel Neuropeptide S Receptor Antagonist with Potent In Vivo Activity
Source: J Med Chem. 2021 Mar 18;64(7):4089–108. doi: 10.1021/acs.jmedchem.0c02223 (PMC8041306; doi:10.1021/acs.jmedchem.0c02223)

Structure-Activity Relationship Studies on Oxazolo[3,4-*a*]Pyrazine Derivatives Leading to the Discovery of a Novel Neuropeptide S Receptor Antagonist With Potent In Vivo Activity

Valentina Albanese[1#], Chiara Ruzza[2,3#], Erika Marzola[1], Tatiana Bernardi[1], Martina Fabbri[1], Anna Fantinati[1], Claudio Trapella[1,3], Rainer K. Reinscheid[4,5], Federica Ferrari[2], Chiara Sturaro[2], Girolamo Calò[6], Giorgio Amendola[7], Sandro Cosconati[7*], Salvatore Pacifico[1*], Remo Guerrini[1,3] and Delia Preti.[1]

[1] Department of Chemical, Pharmaceutical and Agricultural Sciences, University of Ferrara, Via Luigi Borsari 46, 44121 Ferrara, Italy. [2] Department of Neuroscience and Rehabilitation, Section of Pharmacology, University of Ferrara Via Fossato di Mortara 17/19, 44121 Ferrara, Italy. [3]Technopole of Ferrara, LTTA Laboratory for Advanced Therapies, Ferrara, Italy. [4] Institute of Pharmacology and Toxicology, Jena University Hospital, Friedrich Schiller University, Jena, Germany. [5] Institute of Physiology I, University Hospital Münster, University of Münster, Münster, Germany. [6] Department of Pharmaceutical and Pharmacological Sciences, University of Padova, Largo Meneghetti, 2 - 35131 Padova, Italy. [7] "DiSTABiF", Università della Campania "Luigi Vanvitelli", Via Vivaldi 43, 81100 Caserta, Italy.

SUPPORTING INFORMATION

**CONTENTS**	Pag.
Figures S1-S2: ^{1}H-NMR and NOE NMR analysis for compound **17**	S2-S3
Table S1: Human GPCRs sharing with NPSR a sequence identity higher than 20%, a sequence coverage higher than 70%, and that were crystallized in their inactive states.	S4
Figure S3: Phylogenetic tree of the human NPSR and the six selected human GPCRs used as template structures.	S5
Figures S4-S9: pairwise sequence alignments of the human NPSR and the six selected human GPCRs used as template structures.	S6-S11
Figures S10-S15: Ligand Root Mean Square Fluctuation (RMSF) of compounds **1**, **16** and **21**	S12-S14
Figures S16-S27: Ligand-NPSR interactions for compounds **1**, **16** and **21**	S15-S26
HPLC traces of the final compounds **3-21**	S27-S36

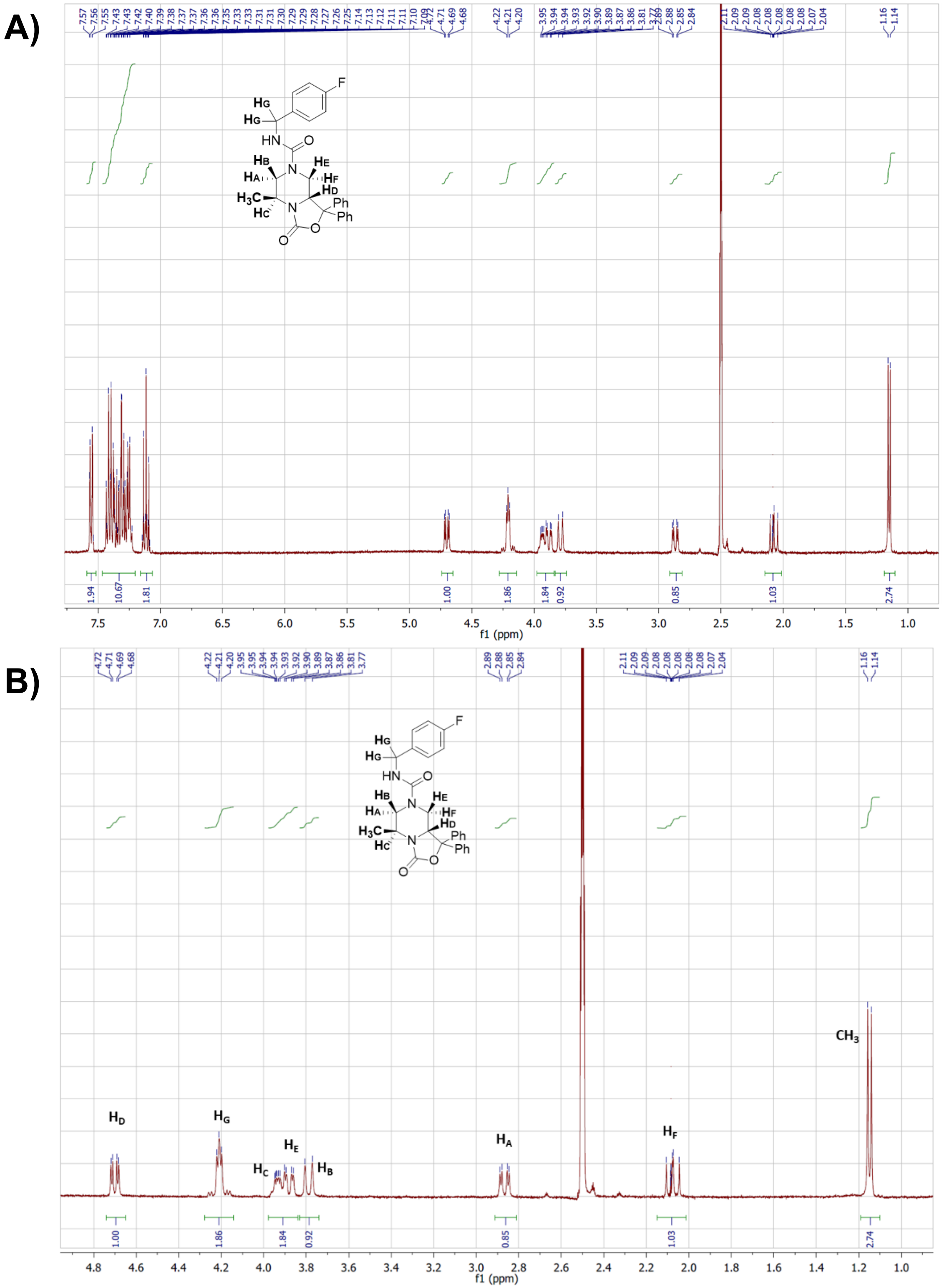

Figure S1. A) Full ^{1}H-NMR spectrum of compound **17**; **B**) Aliphatic region of the ^{1}H-NMR spectrum of compound **17** with proton assignments.

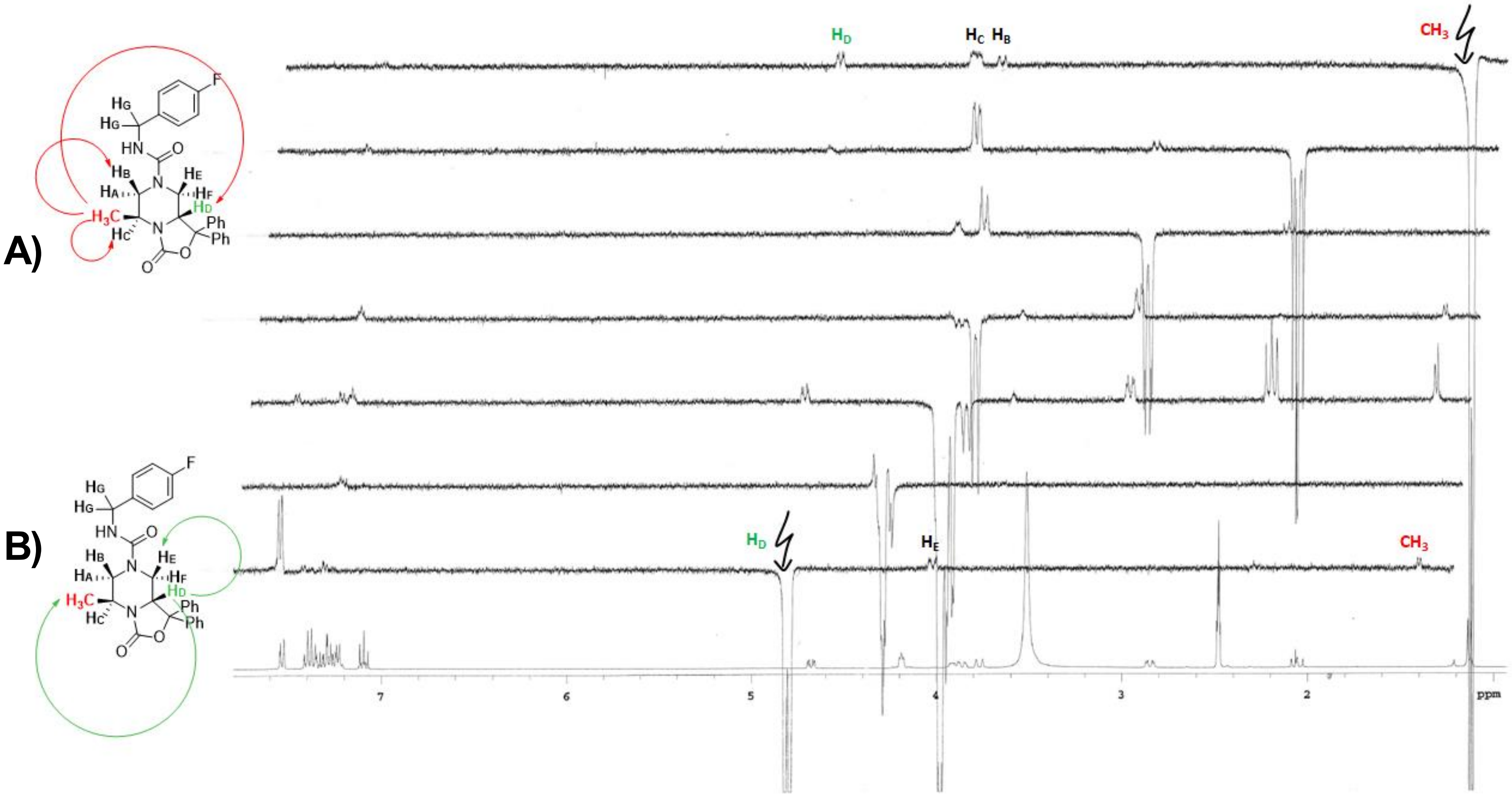

Figure S2. NOE ^{1}H-NMR analysis of compound **17**. **A)** Red arrows indicate that, as visible in the upper trace, irradiating the CH_3 protons produced an enhancement of the signals of $\mathbf{H_C}$ (geminal proton), $\mathbf{H_B}$ and $\mathbf{H_D}$ (that have a *syn* relationship with the irradiated methyl group). **B**) The green arrows show that the irradiation of H_D, produced an enhancement of the signals of the CH_3 protons and of $\mathbf{H_E}$ that have a *syn* relationship with H_D (see the bottom trace).

Table S1. Human GPCRs sharing with NPSR a sequence identity higher than 20%, a sequence coverage higher than 70%, and that were crystallized in their inactive states.

Receptor	Considered PDB	Query Coverage	Sequence Identity	ECL2 conformatio n
**Human C5a anaphylatoxin chemotactic receptor 1**	6C1R	79%	23%	β-hairpin
**Human CC chemokine receptor type 9**	5LWE	73%	21%	Not Present
**Human κ opioid receptor**	4DJH	73%	24%	β-hairpin
**Human M2 muscarinic receptor**	5ZKC	74%	26%	Random Coil
**Human Neuropeptide Y Y1 Receptor**	5ZBH	76%	22%	β-hairpin
**Human orexin-1 receptor**	6TOD	75%	22%	β-hairpin
**Human type-2 angiotensin receptor**	4ZUD	74%	26%	β-hairpin

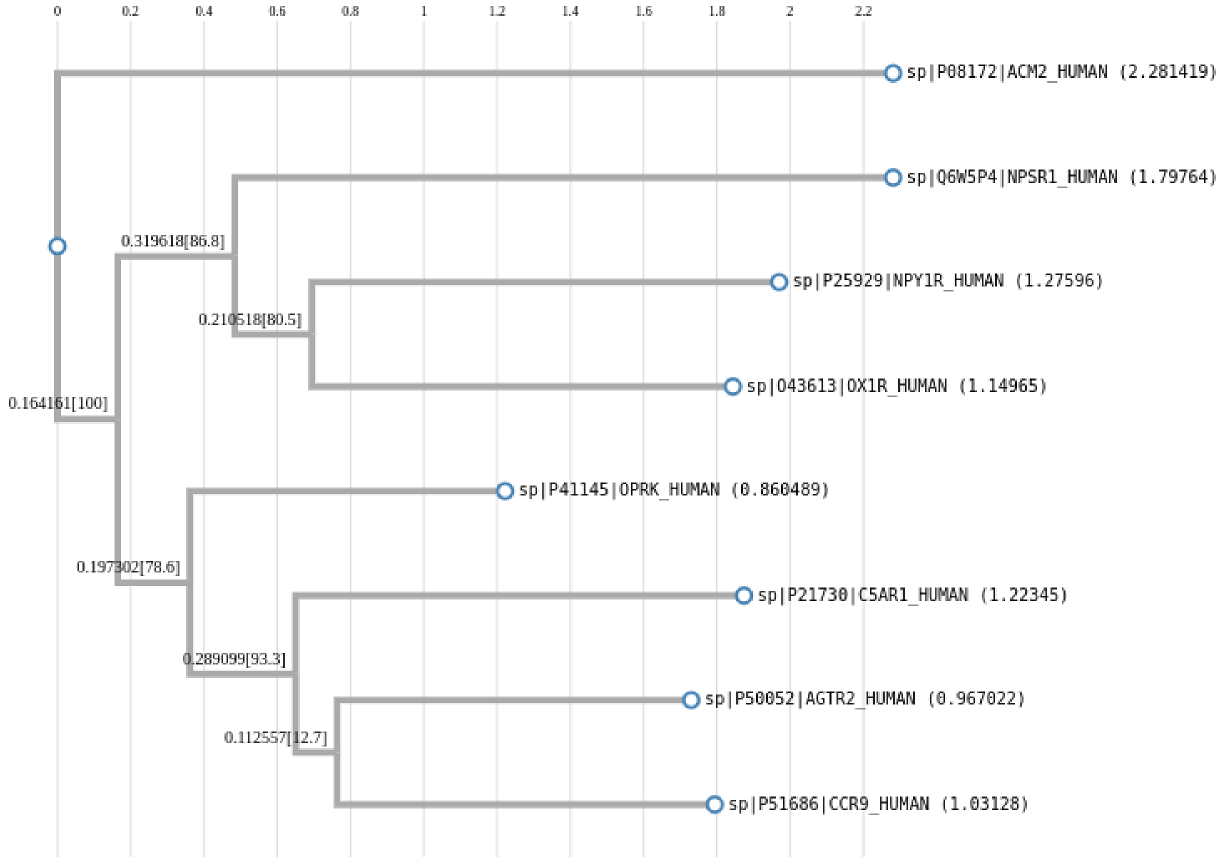

Figure S3. Phylogenetic tree of the human NPSR and the six selected human GPCRs used as template structures.

```
acm2_human      -------------------------------MNNSTNSSNNSLALTSPYKTFEVVFIVLV   29
npsr1_human     MPANFTEGSFDSSGTGQTLDSSPVACTETVTFTEVVEGKEWGSFYYSFK-----TEQLIT   55
                                               :.: .:..: .    *       .  ::.

acm2_human      AGSLSLVTIIGNILVMVSIKVNRHLQTVNNYFLFSLACADLIIGVFSMNLYTLYTV-IGY   88
npsr1_human     LWVLFVFTIVGNSVVLFSTWRRK-KKSRMTFFVTQLAITDSFTGLV-NILTDIIWRFTGD   113
                   * :.**:** :*:.*   .:  ::  .:*: .** :* : *:.   *  :     *

acm2_human      WPLGPVVCDLWLALDYVVSNASVMNLLIISFDRYFCVTKPLTYPVKRTTKMAGMMIAAAW   148
npsr1_human     FTAPDLVCRVVRYLQVVLLYASTYVLVSLSIDRYHAIVYPMKFL--QGEKQARVLIVIAW   171
                :    :** :   *: *:  **.  *: :*:***..:. *:.:   :  * * ::*. **

acm2_human      VLSFILWAPAILFWQFIVGVRTVEDGECYI---QFFSNAAVTFGTAIAAFYLPVIIMTVL   205
npsr1_human     SLSFLFSIPTLII--FGKRTLSNGEVQCWALWPDDSYWTPYMTIVAFLVYFIPLTIISIM   229
                 ***::  *::::  *   . :  : :*:    :    :     .*: .:::*: *::::

acm2_human      YWHISRASKSRIKKDKKEPVANQDPVSPSLVQGRIVKPNNNNMPSSDDGLEHNKIQNGKA   265
npsr1_human     YGIVIRTIWIKSKTYETVISN---------------------------------------   250
                *  : *:   : *. :.

acm2_human      PRDPVTENCVQGEEKESSNDSTSVSAVASNMRDDEITQDENTVSTSLGHSKDENSKQTCI   325
npsr1_human     --------CS--------------------------------------------------   252
                        *

acm2_human      RIGTKTPKSDSCTPTNTTVEVVGSSGQNGDEKQNIVARKIVKMTKQPAKKKPPPSREKKV   385
npsr1_human     ---------------------------------------DGKLCSSYNRGLISKAKIKA   272
                                                             .  ::    . : *.

acm2_human      TRTILAILLAFIITWAPYNVMVLINTFCAPC---IPNTVWTIGYWLCYINSTINPACYAL   442
npsr1_human     IKYSIIIILAFICCWSPYFLFDILDNFNLLPDTQERFYASVIIQNLPALNSAINPLIYCV   332
                 :  : *:****  *:** :: :::.*           . .*   *  :**:***  *.:

acm2_human      CNATFKKTFKHLLM-CHYKNIGATR--------------         466
npsr1_human     FSSSISFPCREQRSQDSRMTFRERTERHEMQILSKPEFI         371
                 .:::.   :.        .:
```

Figure S4. Pairwise sequence alignment as calculated through the gpcrdb.org webserver between human M2 muscarinic receptor and the human neuropeptide S receptor.

```
agtr2_human      -----MKGNSTLATTSKNITSGLHFGLVNISGNNESTLNCSQKPSDKHLDAIPILYYIIF   55
npsr1_human      MPANFTEGSFDSSGTGQTLDSSPVACTETVTFTEVVEGKEWGSFYYSFKTEQLITLWVLF   60
                       :*.   : *.:.: *.      .:: .:    :   .   ..     *  :::*

agtr2_human      VIGFLVNIVVVTLFCCQKGPKKVSSIYIFNLAVADLLLLATLPLWATYYSYRYDWLFGPV   115
npsr1_human      VFTIVGNSVVLFSTWRRKK-KSRMTFFVTQLAITDSFTGLVNILTDIIWRFTGDFTAPDL   119
                 *: :: * **:     :*  *.  :::: :**::* :   .  *    : :  *:    :

agtr2_human      MCKVFGSFLTLNMFASIFFITCMSVDRYQSVIYPFLSQRRNPWQASYIVPLVWCMACLSS   175
npsr1_human      VCRVVRYLQVVLLYASTYVLVSLSIDRYHAIVYPMKFL-QGEKQARVLIVIAWSLSFLFS   178
                 :*:*.  : .: ::** :.:..:*:***::::**:    :.  **  :: :.*.:: * *

agtr2_human      LPTFYFRDVRTIEYLGVNACIMAFPPEKYAQWSAGIALMKNILGFIIPLIFIATCYFGIR   235
npsr1_human      IPTLIIFGKRTLSN-GEVQCWALWPDD---SYWTPYMTIVAFLVYFIPLTIISIMYGIVI   234
                 :**: : . **:.  *   *   :* :   .: :    :  :* ::*** :*:  *  :

agtr2_human      KHLLKTN-------------------SYGKNRITRDQVLKMAAAVVLAFIICWLPFHVLT   276
npsr1_human      RTIWIKSKTYETVISNCSDGKLCSSYNRGLISKAKIKAIKYSIIIILAFICCWSPYFLFD   294
                 : :  ..                   . *    :: :.:* :  ::**** ** *:.::

agtr2_human      FLDALAWMGVINSCEVIAVIDLALPFAILLGFTNSCVNPFLYCFVGNRFQQKLRSVFRVP   336
npsr1_human      ILDNFNLL------PDTQERFYASVIIQNLPALNSAINPLIYCVFSSSISFPCREQRSQ-   347
                 :** :  :              *  :   *   **.:**::**.... :.   *.

agtr2_human      ITWLQGKRESMSCRKSSSLREMETFVS    363
npsr1_human      DSRMTFRERTERHEMQILSKPEFI---    371
                  : :  :..:   . .   :
```

Figure S5. Pairwise sequence alignment as calculated through the gpcrdb.org webserver between human type-2 angiotensin receptor and the human neuropeptide S receptor.

```
c5ar1_human     ----------------MDSFNYTTPDYGHYDDKDTLDLNTPVDKTSNTLRVPDILALVIF   44
npsr1_human     MPANFTEGSFDSSGTGQTLDSSPVACTETVTFTEVVEGKEWGSFYYSFK----TEQLITL   56
                                    .  .        .:.:: :   .   .         *: :

c5ar1_human     AVVFLVGVLGNALVVWVTAFEAKRTINAIWFLNLAVADFLSCLALPILFTSIVQHHHWPF   104
npsr1_human     WVLFVFTIVGNSVVLFSTWRRKKKSRMTFFVTQLAITDSFTGLVNILTDIIWRFTGDFTA   116
                 *:*:. ::**::*:: *  . *::  :::. :**::* :: *.  :         .:

c5ar1_human     GGAACSILPSLILLNMYASILLLATISADRFLLVFKPIWCQNFRGAGLAWIACAVAWGLA   164
npsr1_human     PDLVCRVVRYLQVVLLYASTYVLVSLSIDRYHAIVYPMKFL--QGEKQARVLIVIAWSLS   174
                 . .* ::  * :: :***  :*.::* **:  :. *:     :*   * :  .:**.*:

c5ar1_human     LLLTIPSFLYRV---VREEYFPPKVLCGVDYSHDKRRERAVAIVRLVLGFLWPLLTLTIC   221
npsr1_human     FLFSIPTLIIFGKRTLSNGEV----QCWALWP-DDSYWTPYMTIVAFLVYFIPLTIISIM   229
                :*::**:::      : :  .     * . :  *.        :  .* :: **  ::*

c5ar1_human     YTFILLRTWSRRAT-----------------------RSTKTLKVVVAVVASFFIFWLP   257
npsr1_human     YGIVIRTIWIKSKTYETVISNCSDGKLCSSYNRGLISKAKIKAIKYSIIIILAFICCWSP   289
                * :::   * :  *                         . *::*  : :: :*:  * *

c5ar1_human     YQVTGIMMSFLEPSSPTFLLLKKLDSLCVSFAYINCCINPIIYVVAGQGFQGRLRK----   313
npsr1_human     YFLFDILDNFNLL--PDTQERFYASVIIQNLPALNSAINPLIYCVFSSSISFPCREQRSQ   347
                * : .*: .*     *        . :  .:  :*..***:** * ...:.   *:

c5ar1_human     SLPSLLRNVLTEESVVRESKSFTRSTVDTMAQKTQAV  350
npsr1_human     DSRMTFRERTERHEMQILSKPEFI-------------  371
                .    :*:   ...:   **
```

Figure S6. Pairwise sequence alignment as calculated through the gpcrdb.org webserver between human C5a anaphylatoxin chemotactic receptor 1 and the human neuropeptide S receptor.

```
npsr1_human      MPANFTEGSFDSSGTGQTLDSSPVACTETVTFTEVVEGKEWGSFYYSFK-----TEQLIT   55
npy1r_human      --------------MNSTLFSQVENHSVHSNFSEKNAQLLAFENDDCHLPLAMIFTLALA   46
                                ..** *.    :   .*:*        .   ..          ::

npsr1_human      LWVLFVFTIVGNSVVLFSTWRRKK-KSRMTFFVTQLAITDSFTGL-VNILTDIIWRFTGD   113
npy1r_human      YGAVIILGVSGNLALIIIILKQKEMRNVTNILIVNLSFSDLLVAIMCLPFTFVYTLMD-H   105
                   .:::: : ** .:::   ::*: :.  .:::.:*:::* :..:    :* :   :  .

npsr1_human      FTAPDLVCRVVRYLQVVLLYASTYVLVSLSIDRYHAIVYPMKFLQGEKQARVLIVIAWSL   173
npy1r_human      WVFGEAMCKLNPFVQCVSITVSIFSLVLIAVERHQLIINPRGWRPNNRHAYVGIAVIWVL   165
                 :.  : :*::  ::* * : .* : ** ::::*:: *: *  :  .:::* * *.: * *

npsr1_human      SFLFSIPTLIIFGKRT---------LSNGEVQCWALWPDDSYWTPYMTIVAFLVYFIPLT   224
npy1r_human      AVASSLPFLIYQVMTDEPFQNVTLDAYKDKYVCFDQFPSDSHRLSYTTLLLVLQYFGPLC   225
                 :.  *:* **                 :.:  *:  :*.**:   * *:: .* ** **

npsr1_human      IISIMYGIVIRTIWIKSKTYETVISNCSDGKLCSSYNRGLISKAKIKAIKYSIIIILAFI   284
npy1r_human      FIFICYFKIYIRLKRRNNMMDKMR------------DNKYRSSETKRINIMLLSIVVAFA   273
                 :* * *  :   :  :.:  :.:             :.   *. . :     : *::**

npsr1_human      CCWSPYFLFDILDNFNLL-PDTQERFYASVIIQNLPALNSAINPLIYCVFSSSISFPCRE   343
npy1r_human      VCWLPLTIFNTVFDWNHQIIATCNHNLLFLLCHLTAMISTCVNPIFYGFLNKNFQRDLQF   333
                  ** *  :*: : ::*     * ::    :: :    :.:.:**::* .:...:.   :

npsr1_human      QRSQDSRMTFRERTERHEMQILSKPEFI-------------------------  371
npy1r_human      FF--NFCDFRSRDDDYETIAMSTMHTDVSKTSLKQASPVAFKKINNNDDNEKI  384
                     :      .  : . : : :    :
```

Figure S7. Pairwise sequence alignment as calculated through the gpcrdb.org webserver between human neuropeptide Y Y1 receptor and the human neuropeptide S receptor.

```
npsr1_human      -------MPANFTEGSFDSSGTGQTLDSSPVACTETVTFTEVVEGKEWGSFYYSFK---T   50
oprk_human       MDSPIQIFRGEPGPTCAPSACLPPNSSAWFPGWAEPDSNGSAGSEDAQLEPAHISPAIPV   60
                        : .:    .  *:    . .:   . :*  :  .. . .   .  :      .

npsr1_human      EQLITLWVLFVFTIVGNSVVLFSTWRRKK-KSRMTFFVTQLAITDSFTGLVNILTDIIWR   109
oprk_human       IITAVYSVVFVVGLVGNSLVMFVIIRYTKMKTATNIYIFNLALADALVTTTMPFQSTVYL   120
                     .  *:**. :****:*:*   * .* *:  .::: :**::*::.  .  : . ::

npsr1_human      FTGDFTAPDLVCRVVRYLQVVLLYASTYVLVSLSIDRYHAIVYPMKFL--QGEKQARVLI   167
oprk_human       M-NSWPFGDVLCKIVISIDYYNMFTSIFTLTMMSVDRYIAVCHPVKALDFRTPLKAKIIN   179
                 : ..:   *::*::*  ::   :::* :.*. :*:*** *: :*:* *  :   :*:::

npsr1_human      VIAWSLSFLFSIPTLIIFGKR-TLSNGEVQCWALWPDDSY---WTPYMTIVAFLVYFIPL   223
oprk_human       ICIWLLSSSVGISAIVLGGTKVREDVDVIECSLQFPDDDYSWWDLFMKICVFIFAFVIPV   239
                 :  * **  ..* :::: *.:   . . ::*   :***.*          * ::.:.**:

npsr1_human      TIISIMYGIVIRTIWIKSKTYETVISNCSDGKLCSSYNRGLISKAKIKAIKYSIIIILAF   283
oprk_human       LIIIVCYTLMILRLKSVRLL----------------SGSREKDRNLRRITRLVLVVVAVF   283
                  ** : * ::*  :                       .    .:   :  :  :::: .*

npsr1_human      ICCWSPYFLFDILDNFNLLPDTQERFYASVIIQNLPALNSAINPLIYCVFSSSISFPCRE   343
oprk_human       VVCWTPIHIFILVEALGSTSHSTAALSSYYFCIALGYTNSSLNPILYAFLDENFKRCFRD   343
                 : **:* .:* ::: :.   .:   : :  :   *   **::**::*..:...:.   *:

npsr1_human      QRSQDSRMTFRERTERHEMQILSKPEFI---------- 371
oprk_human       FCF-PLKMRMERQSTSRVRNTVQDPAYLRDIDGMNKPV 380
                       :* :..::  :  : :..* ::
```

Figure S8. Pairwise sequence alignment as calculated through the gpcrdb.org webserver between human κ opioid receptor and the human neuropeptide S receptor.

```
npsr1_human      MPANFTEGSFDSSGTGQTLDSSPVACTETVTFTEVVEGKEWGSFYYSFK-TEQLITLWVL   59
ox1r_human       ----MEPSATPGAQMGVPPGSREPSPVPPDYEDEFLRYLWRDYLYPKQYEWVLIAAYVAV   56
                     :  .:  .:  *   .*   : .      *.:.    . :* .      : :  .:

npsr1_human      FVFTIVGNSVVLFSTWRRKK-KSRMTFFVTQLAITDSFTGL-VNILTDIIWRFTGDFTAP   117
ox1r_human       FVVALVGNTLVCLAVWRNHHMRTVTNYFIVNLSLADVLVTAICLPASLLVDI-TESWLFG   115
                 **.::***::* ::.**.:: ::  .:*:.:*:::* :.       : ::   * .:

npsr1_human      DLVCRVVRYLQVVLLYASTYVLVSLSIDRYHAIVYPMKFLQGEKQARVLIVIAWSLSFLF   177
ox1r_human       HALCKVIPYLQAVSVSVAVLTLSFIALDRWYAICHPLLFKSTARRARGSILGIWAVSLAI   175
                 . :*:*: ***.* : .:. .*  :::**::** :*: * .  ::**  *:  *::*: :

npsr1_human      SIPTLIIFGKRTL-------SNGEVQCWALWPDDSYWTPYMTIVAFLVYFIPLTIISIMY   230
ox1r_human       MVPQAAVMECSSVLPELANRTRLFSVCDERWADDLYPKIYHSCFFIVTYLAPLGLMAMAY   235
                  :*   ::   ::       :.    *   * ** * . * : . ::.*: ** :::: *

npsr1_human      GIVIRTIWIKSKTYETVI---------------SNCSDGKLCSSYN----RGLISKAKIK   271
ox1r_human       FQIFRKLWGRQIPGTTSALVRNWKRPSDQLGDLEQGLSGEPQPRARAFLAEVKQMRARRK   295
                   ::*.:* :.    *                 .:  .*:     .    .    :*: *

npsr1_human      AIKYSIIIILAFICCWSPYFLFDILDNFNLL----PDTQERFYASVIIQNLPALNSAINP   327
ox1r_human       TAKMLMVVLLVFALCYLPISVLNVLKRVFGMFRQASDREAVYACFTFSHWLVYANSAANP   355
                 : *  ::::*.*  *: *  ::::*...  :     * :  : . .: : *   *** **

npsr1_human      LIYCVFSSSISFPCREQRSQDSRMTFRERTERHEMQILSKPEFI----------------   371
ox1r_human       IIYNFLSGKFREQFKAAF--SCCLPGLGPCGSLKAPSPRSSASHKSLSLQSRCSISKISE   413
                 :** .:*..:    :     .. :         :     .

npsr1_human      ------------   371
ox1r_human       HVVLTSVTTVLP   425
```

Figure S9. Pairwise sequence alignment as calculated through the gpcrdb.org webserver between human orexin-1 receptor and the human neuropeptide S receptor.

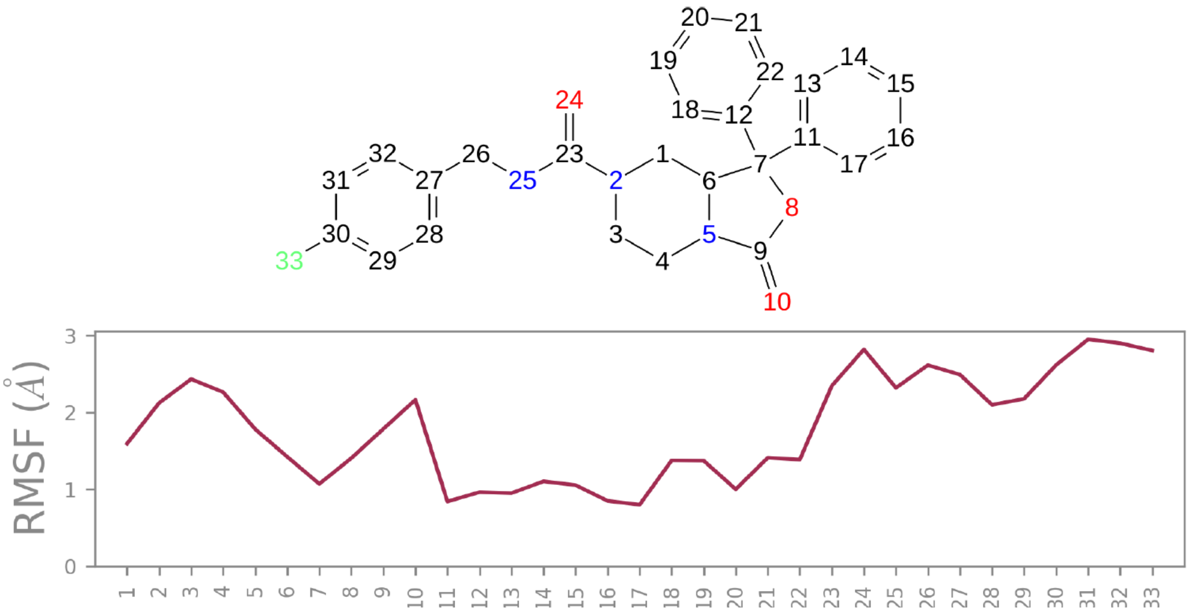

Figure S10. Ligand Root Mean Square Fluctuation (L-RMSF) of **1** in MD1 broken down by atom, corresponding to the 2D structure in the top panel.

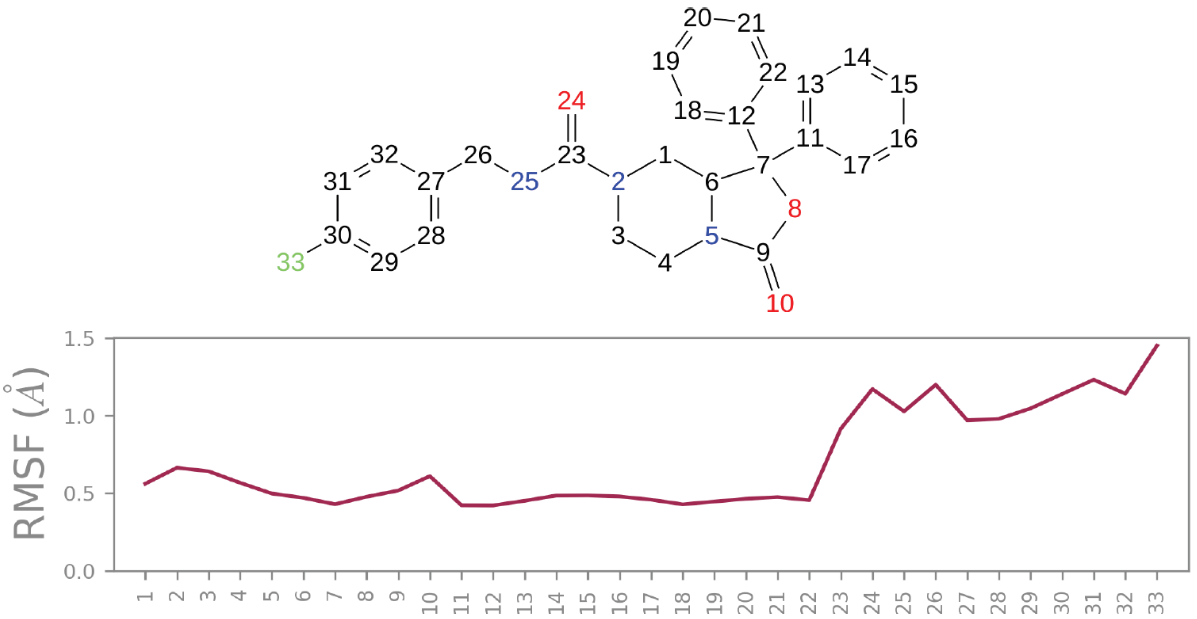

Figure S11. Ligand Root Mean Square Fluctuation (L-RMSF) of **1** in MD2 broken down by atom, corresponding to the 2D structure in the top panel.

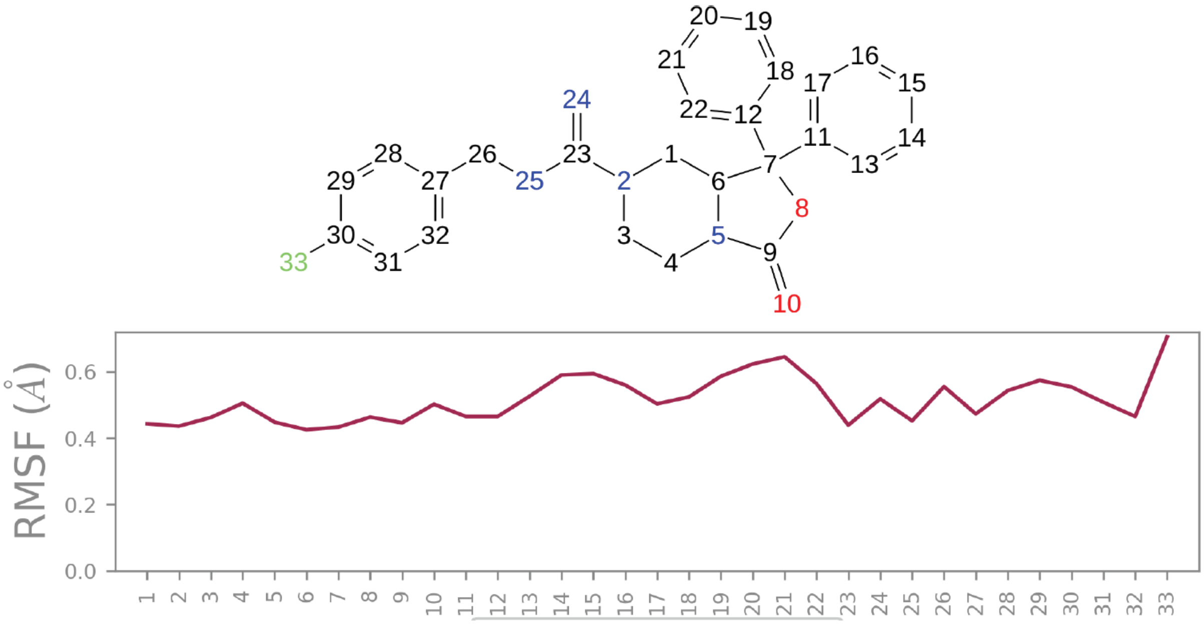

Figure S12. Ligand Root Mean Square Fluctuation (L-RMSF) of **16** in MD1 broken down by atom, corresponding to the 2D structure in the top panel.

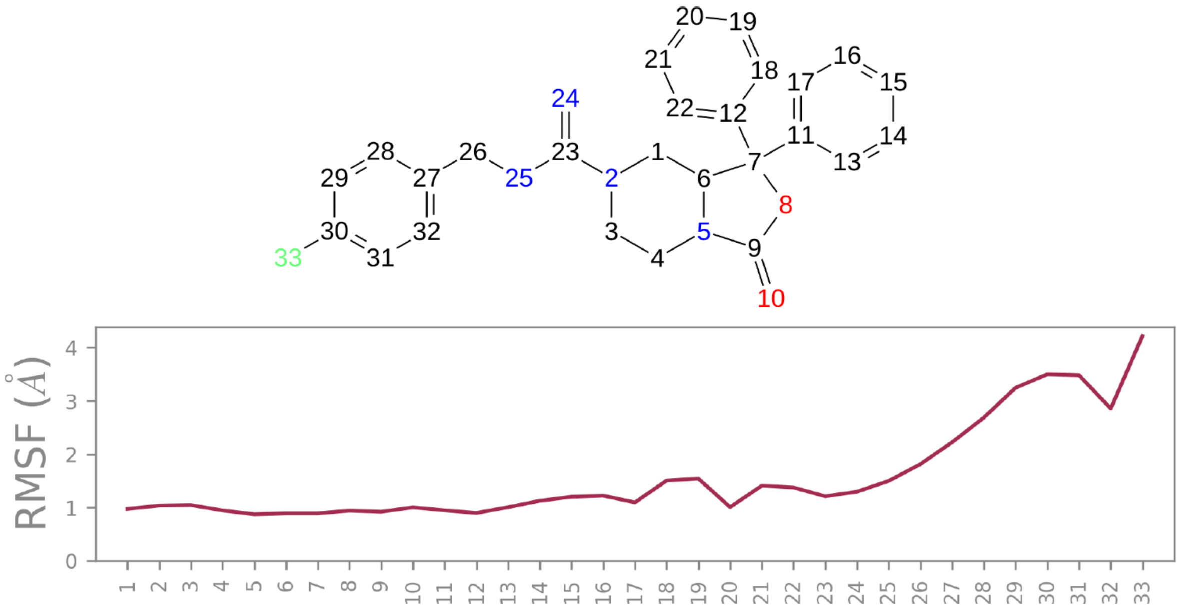

Figure S13. Ligand Root Mean Square Fluctuation (L-RMSF) of **16** in MD2 broken down by atom, corresponding to the 2D structure in the top panel.

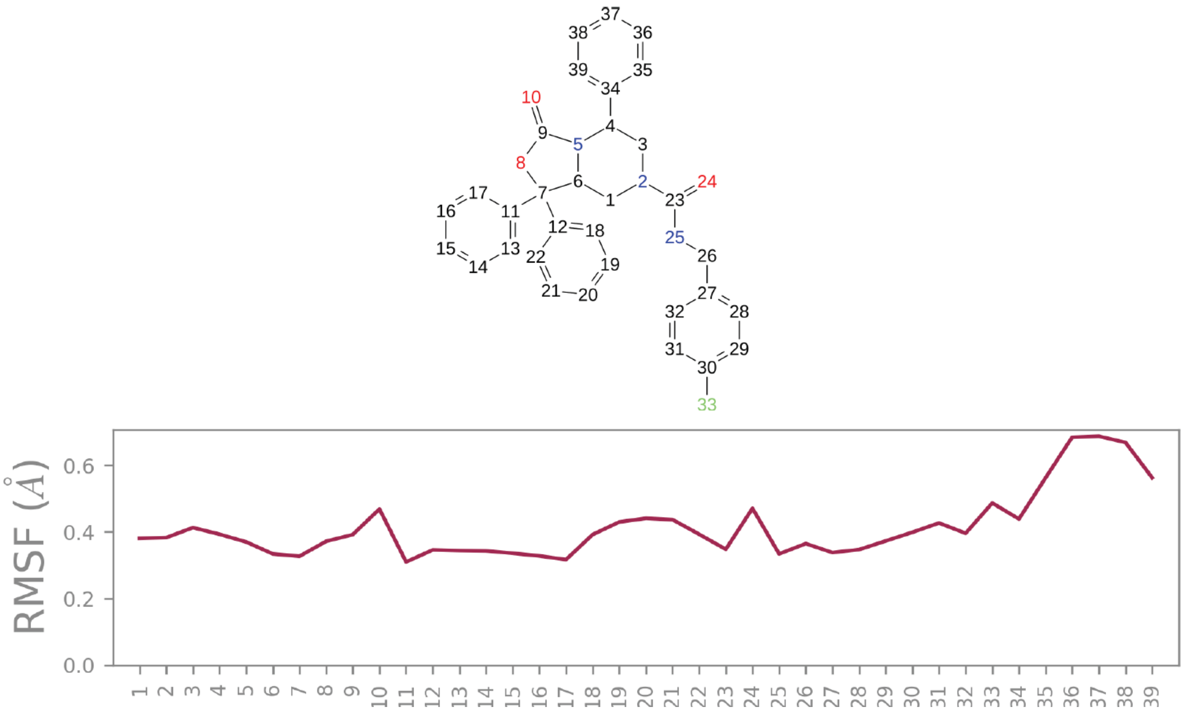

Figure S14. Ligand Root Mean Square Fluctuation (L-RMSF) of **21** in MD1 broken down by atom, corresponding to the 2D structure in the top panel.

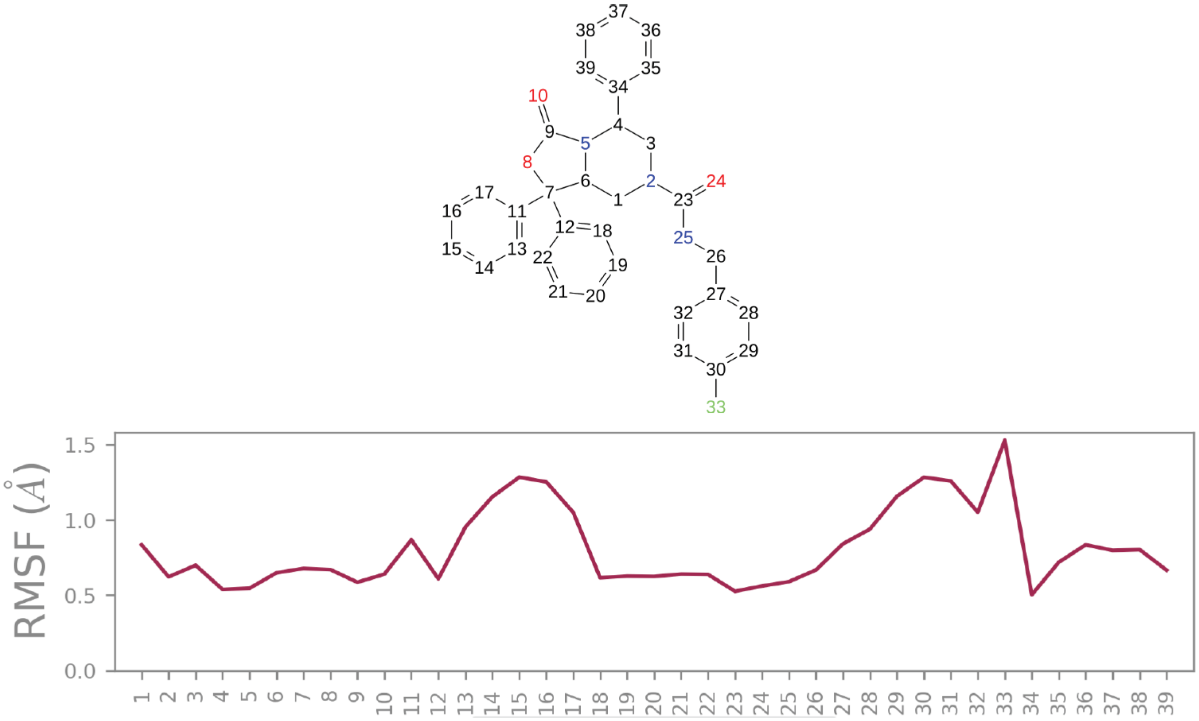

Figure S15. Ligand Root Mean Square Fluctuation (L-RMSF) of **21** in MD2 broken down by atom, corresponding to the 2D structure in the top panel.

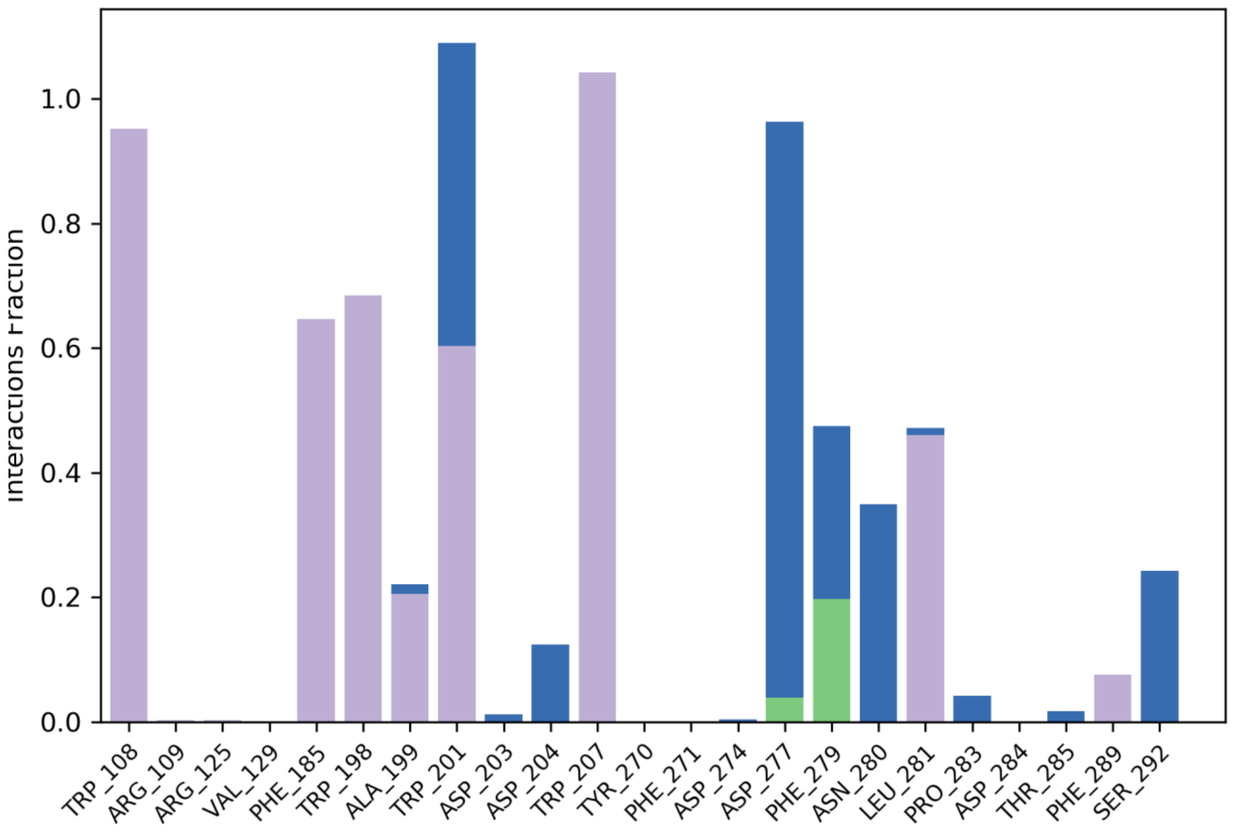

Figure S16. Plot of the ligand-protein interactions between **1** and NPSR in BM1. Protein-ligand interactions (or 'contacts') are categorized into four types: Hydrogen Bonds (green bars), Hydrophobic (violet bars), Ionic (magenta bars) and Water Bridges (blue bars). The stacked bar charts are normalized over the course of the trajectory: for example, a value of 0.7 suggests that 70% of the simulation time the specific interaction is maintained. Values over 1.0 are possible as some protein residue may make multiple contacts of same subtype with the ligand.

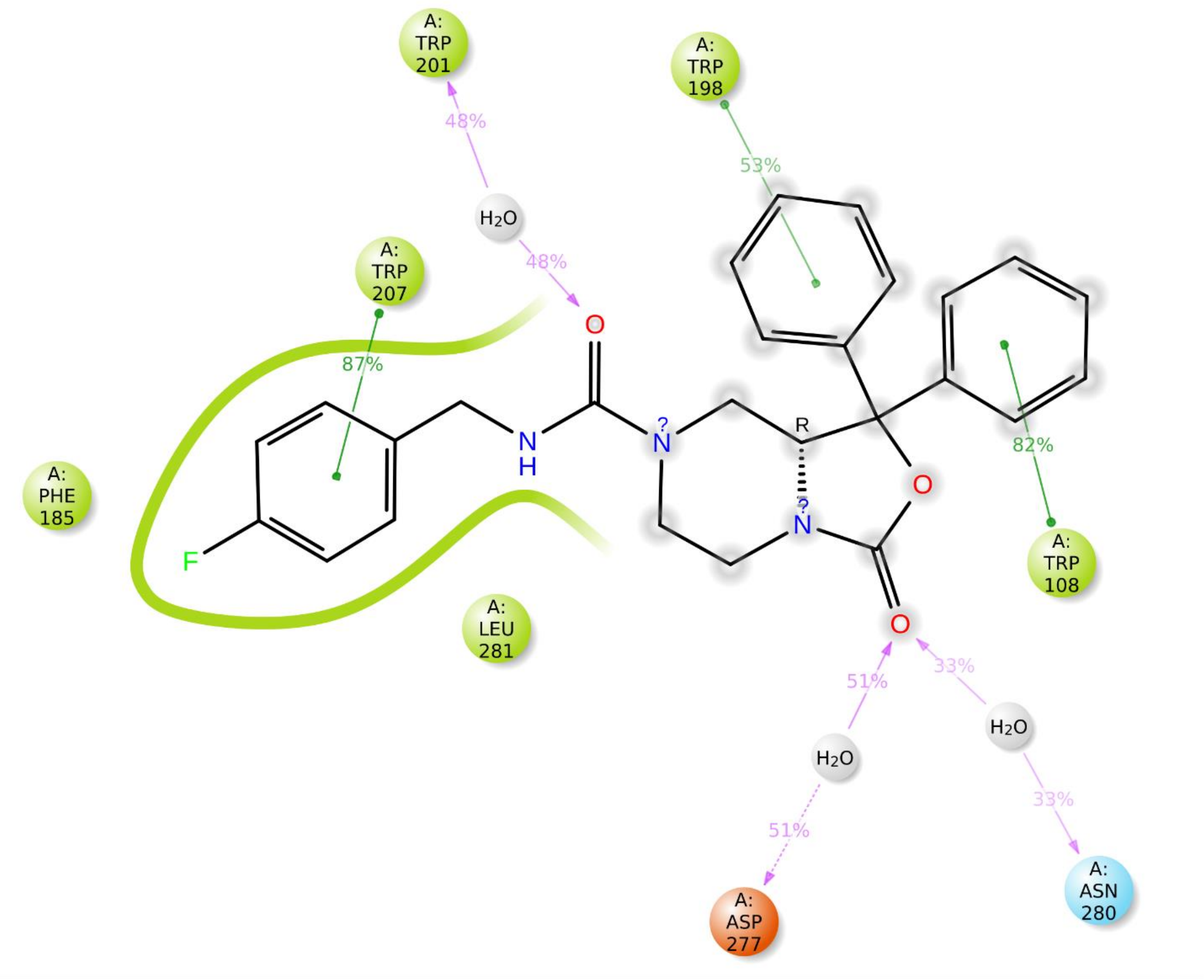

Figure S17. Schematic of detailed ligand atom interactions between **1** and NPSR during the MD simulation in BM1. Interactions that occur more than 30.0% of the simulation time in the selected trajectory (0.00 through 100.00 ns), are shown.

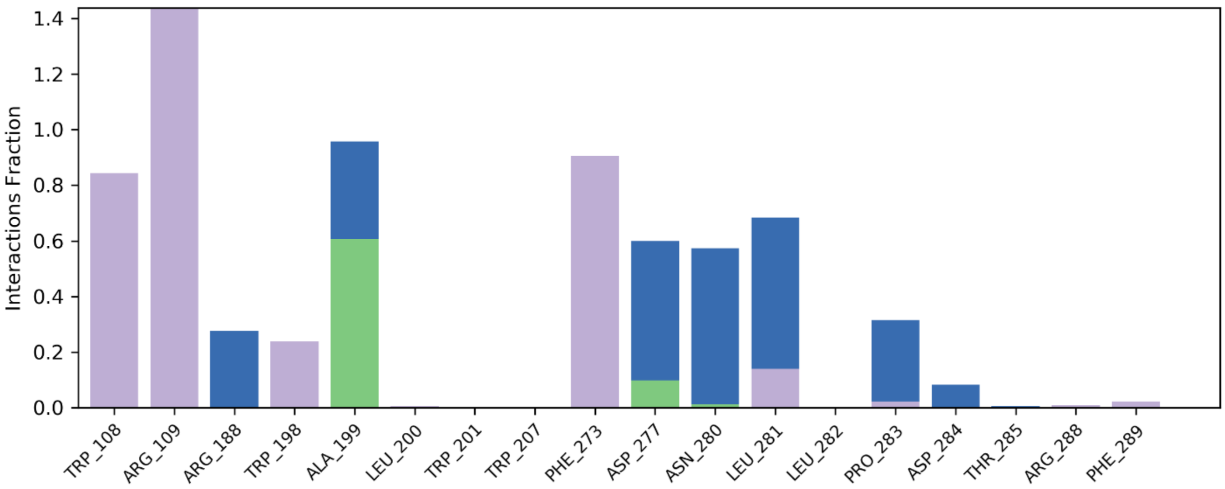

Figure S18. Plot of the ligand-protein interactions between **1** and NPSR in BM2 throughout the simulation. Protein-ligand interactions (or 'contacts') are categorized into four types: Hydrogen Bonds (green bars), Hydrophobic (violet bars), Ionic (magenta bars) and Water Bridges (blue bars). The stacked bar charts are normalized over the course of the trajectory: for example, a value of 0.7 suggests that 70% of the simulation time the specific interaction is maintained. Values over 1.0 are possible as some protein residue may make multiple contacts of same subtype with the ligand.

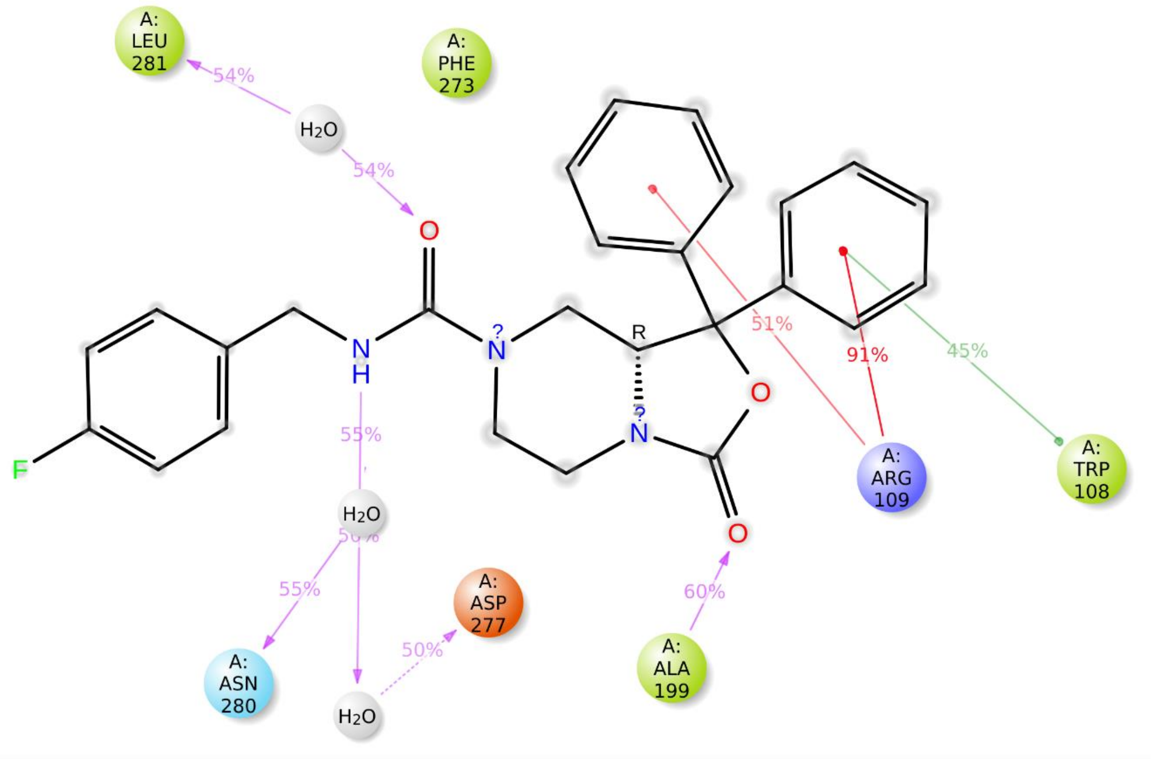

Figure S19. Schematic of detailed ligand atom interactions between **1** and NPSR during the MD simulation in BM2. Interactions that occur more than 30.0% of the simulation time in the selected trajectory (0.00 through 100.00 ns), are shown.

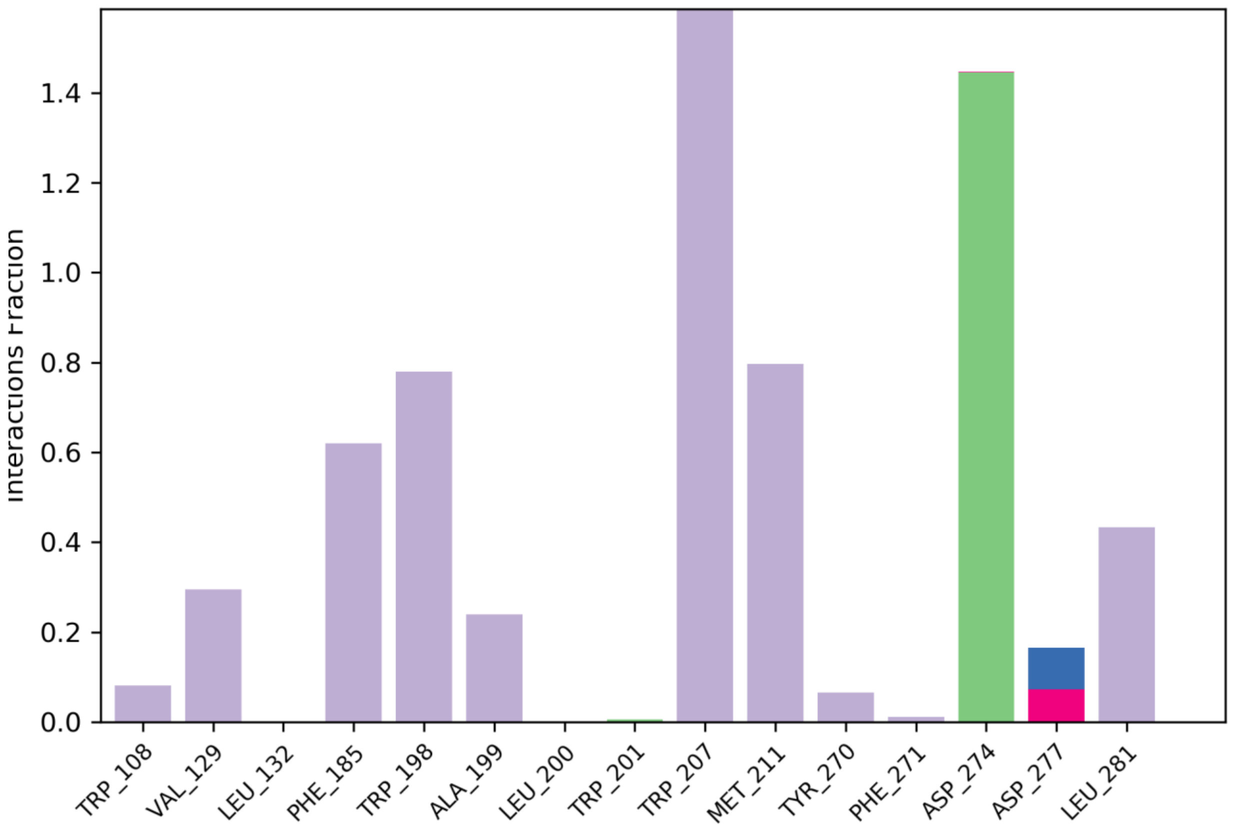

Figure S20. Plot of the ligand-protein interactions between **16** and NPSR in BM1 throughout the simulation. Protein-ligand interactions (or 'contacts') are categorized into four types: Hydrogen Bonds (green bars), Hydrophobic (violet bars), Ionic (magenta bars) and Water Bridges (blue bars). The stacked bar charts are normalized over the course of the trajectory: for example, a value of 0.7 suggests that 70% of the simulation time the specific interaction is maintained. Values over 1.0 are possible as some protein residue may make multiple contacts of same subtype with the ligand.

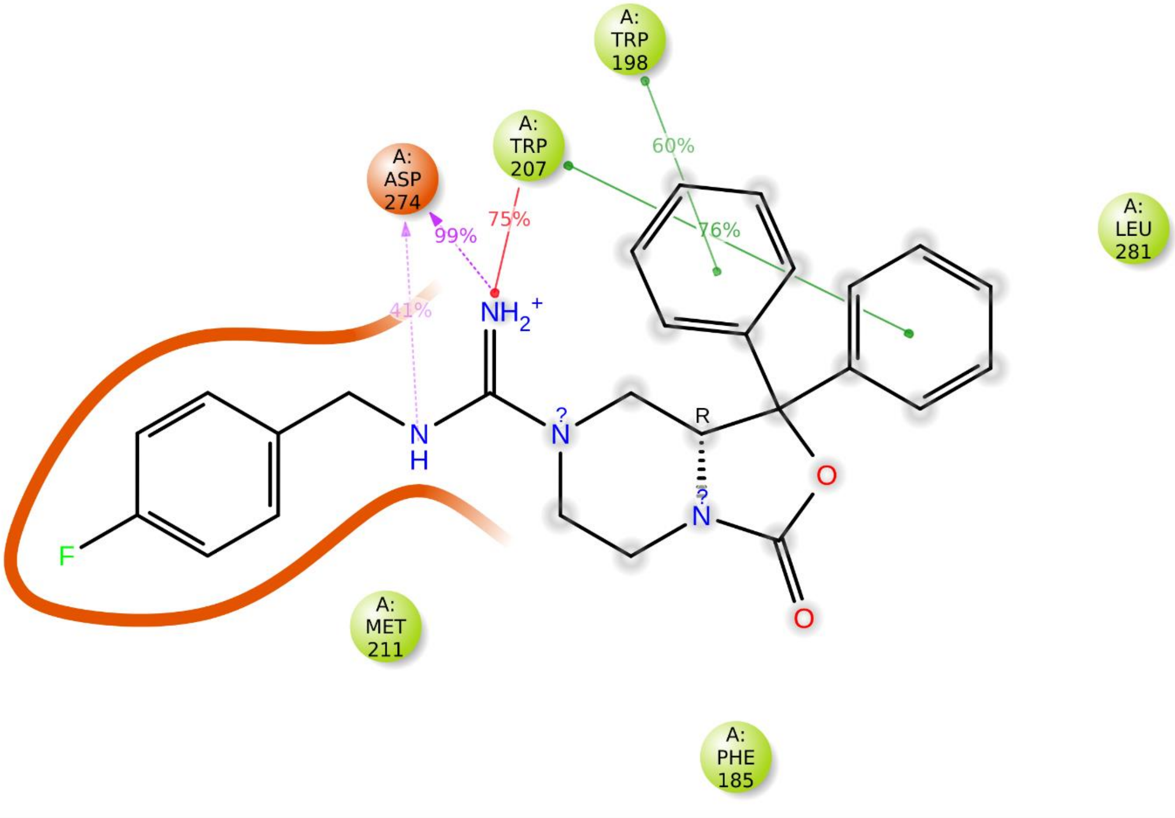

Figure S21. Schematic of detailed ligand atom interactions between **16** and NPSR during the MD simulation in BM1. Interactions that occur more than 30.0% of the simulation time in the selected trajectory (0.00 through 100.00 ns), are shown.

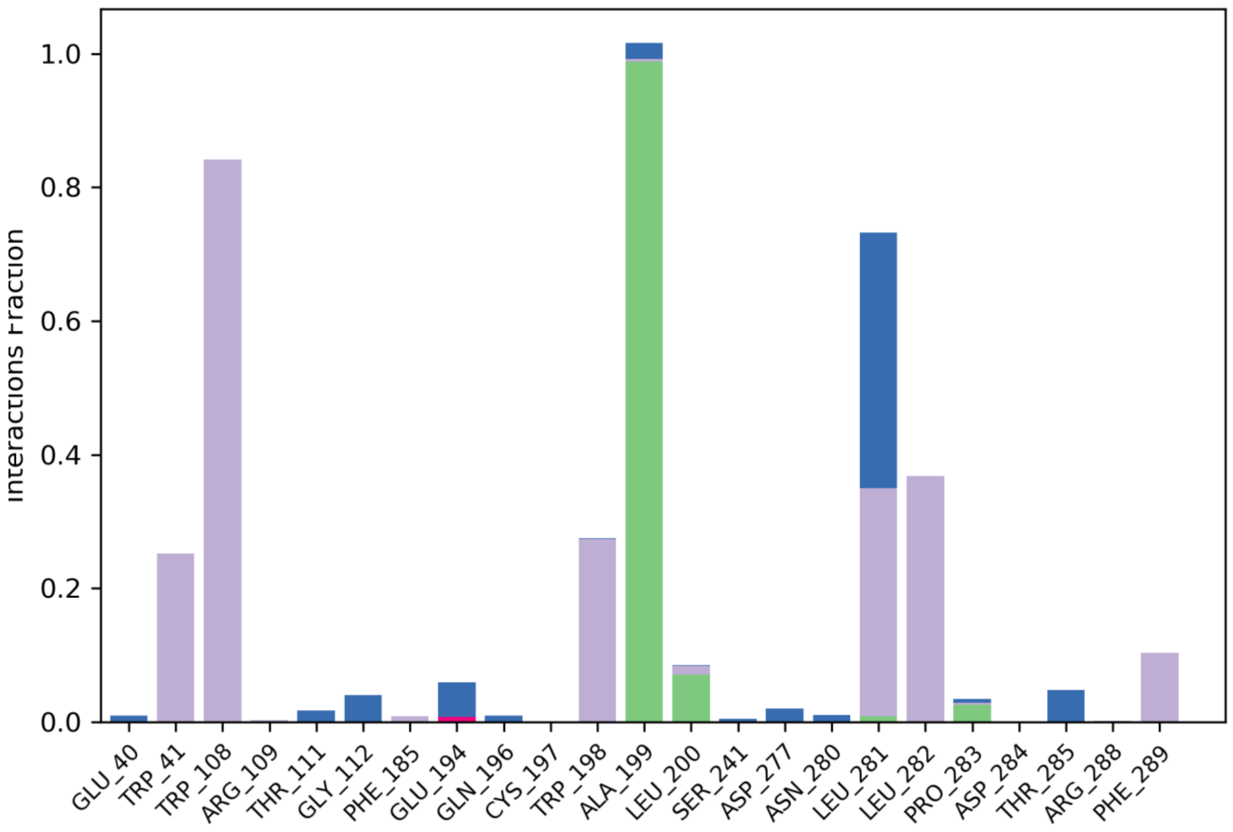

Figure S22. Plot of the ligand-protein interactions between **16** and NPSR in BM2 throughout the simulation. Protein-ligand interactions (or 'contacts') are categorized into four types: Hydrogen Bonds (green bars), Hydrophobic (violet bars), Ionic (magenta bars) and Water Bridges (blue bars). The stacked bar charts are normalized over the course of the trajectory: for example, a value of 0.7 suggests that 70% of the simulation time the specific interaction is maintained. Values over 1.0 are possible as some protein residue may make multiple contacts of same subtype with the ligand.

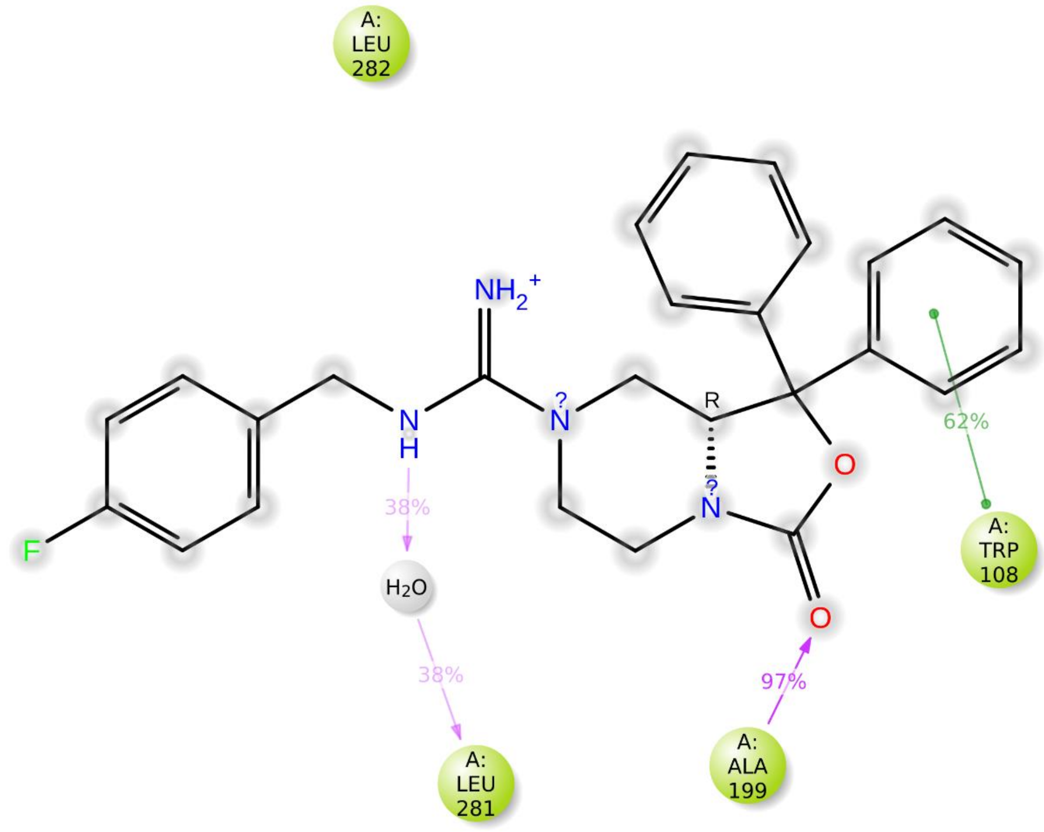

Figure S23. Schematic of detailed ligand atom interactions between **16** and NPSR during the MD simulation in BM2. Interactions that occur more than 30.0% of the simulation time in the selected trajectory (0.00 through 100.00 ns), are shown.

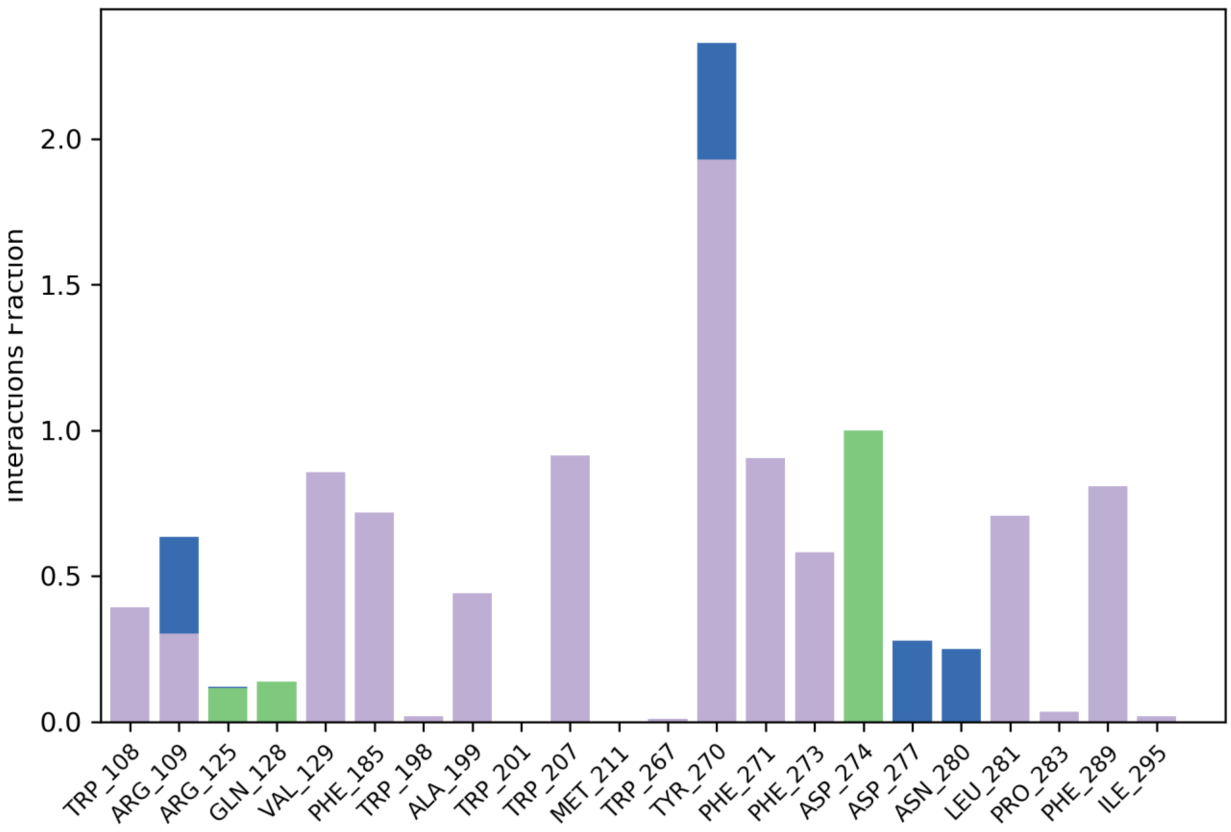

Figure S24. Plot of the ligand-protein interactions between **21** and NPSR in BM1 throughout the simulation. Protein-ligand interactions (or 'contacts') are categorized into four types: Hydrogen Bonds (green bars), Hydrophobic (violet bars), Ionic (magenta bars) and Water Bridges (blue bars).The stacked bar charts are normalized over the course of the trajectory: for example, a value of 0.7 suggests that 70% of the simulation time the specific interaction is maintained. Values over 1.0 are possible as some protein residue may make multiple contacts of same subtype with the ligand.

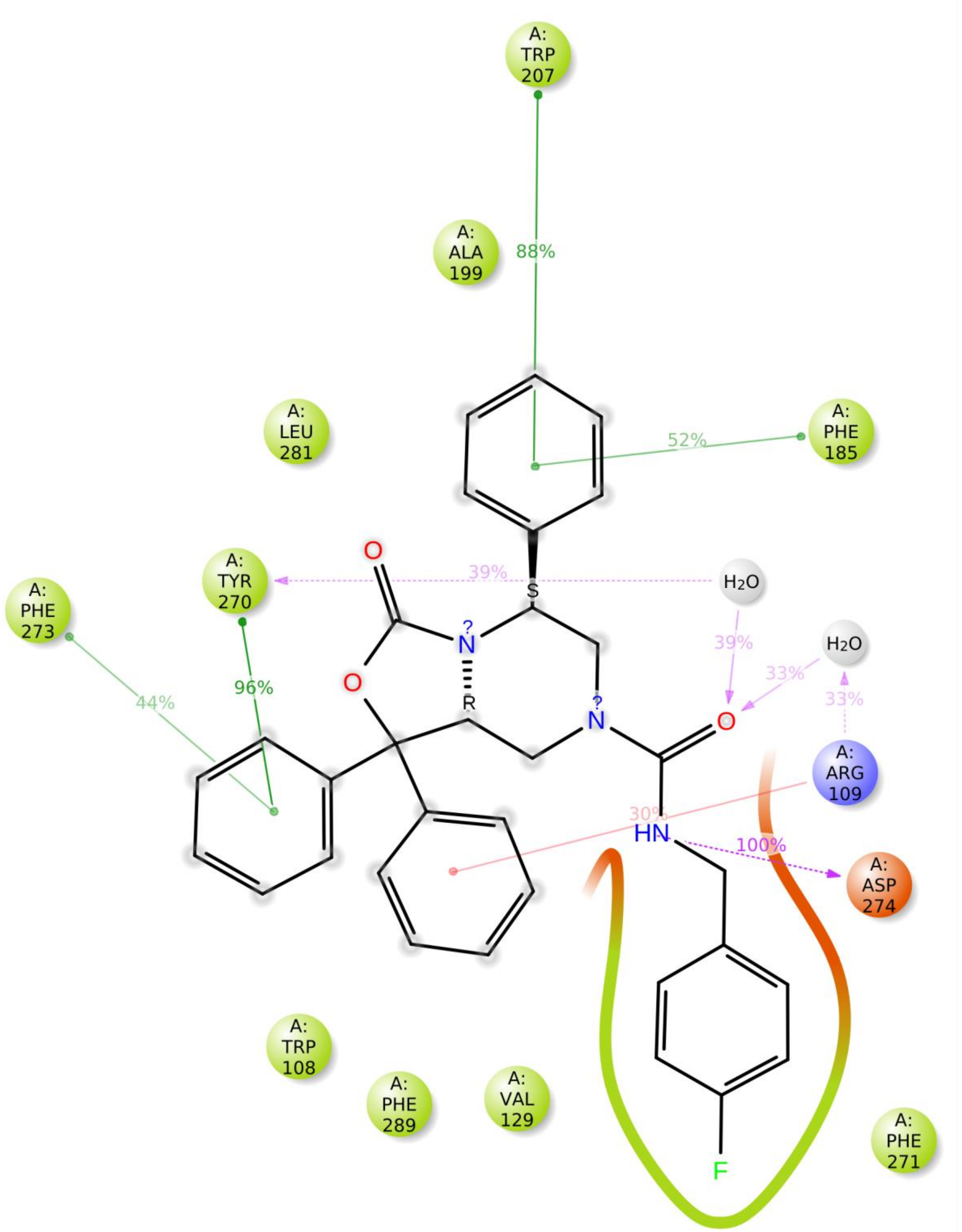

Figure S25. Schematic of detailed ligand atom interactions between **21** and NPSR during the MD simulation in BM1. Interactions that occur more than 30.0% of the simulation time in the selected trajectory (0.00 through 100.00 ns), are shown.

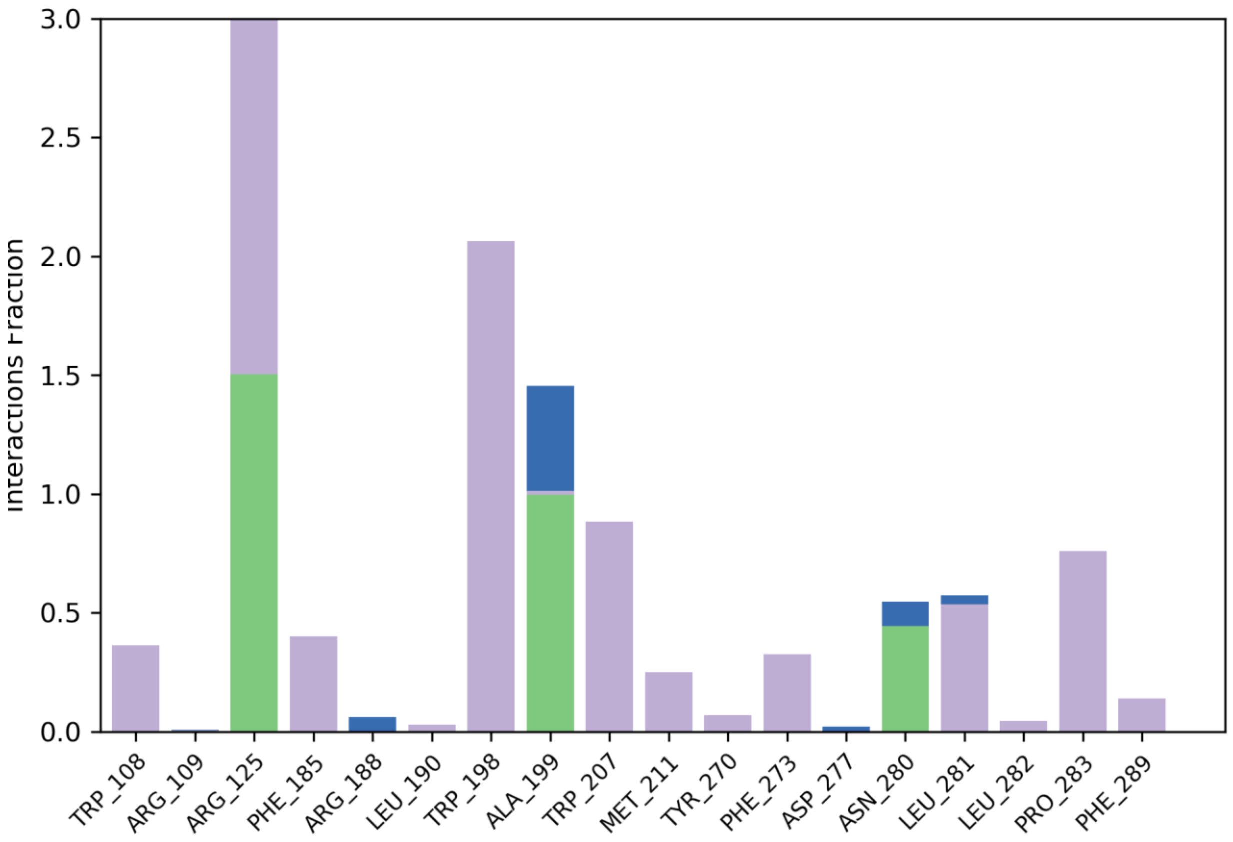

Figure S26. Plot of the ligand-protein interactions between **21** and NPSR in BM2 throughout the simulation. Protein-ligand interactions (or 'contacts') are categorized into four types: Hydrogen Bonds (green bars), Hydrophobic (violet bars), Ionic (magenta bars) and Water Bridges (blue bars). The stacked bar charts are normalized over the course of the trajectory: for example, a value of 0.7 suggests that 70% of the simulation time the specific interaction is maintained. Values over 1.0 are possible as some protein residue may make multiple contacts of same subtype with the ligand.

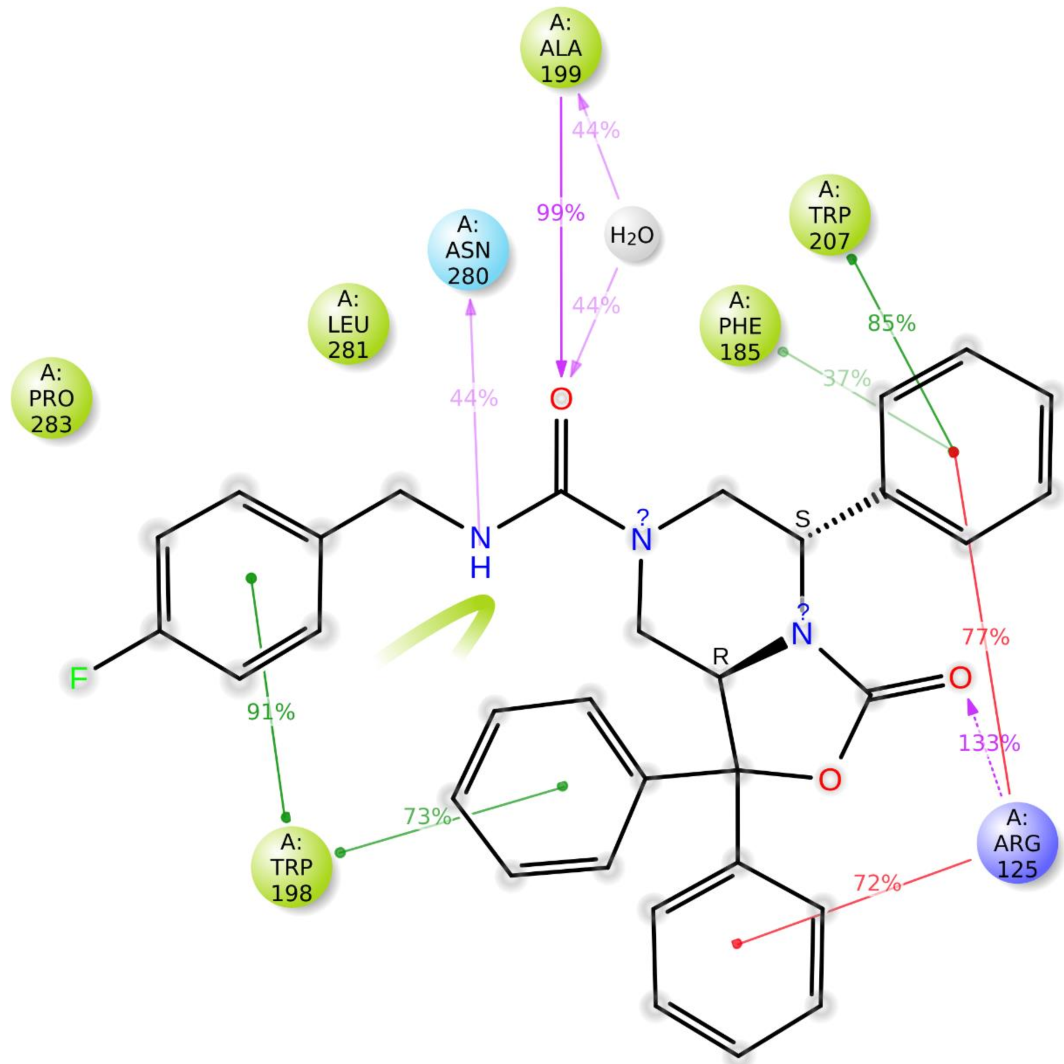

Figure S27. Schematic of detailed ligand atom interactions between **21** and NPSR during the MD simulation in BM2. Interactions that occur more than 30.0% of the simulation time in the selected trajectory (0.00 through 100.00 ns), are shown.

Compound 3

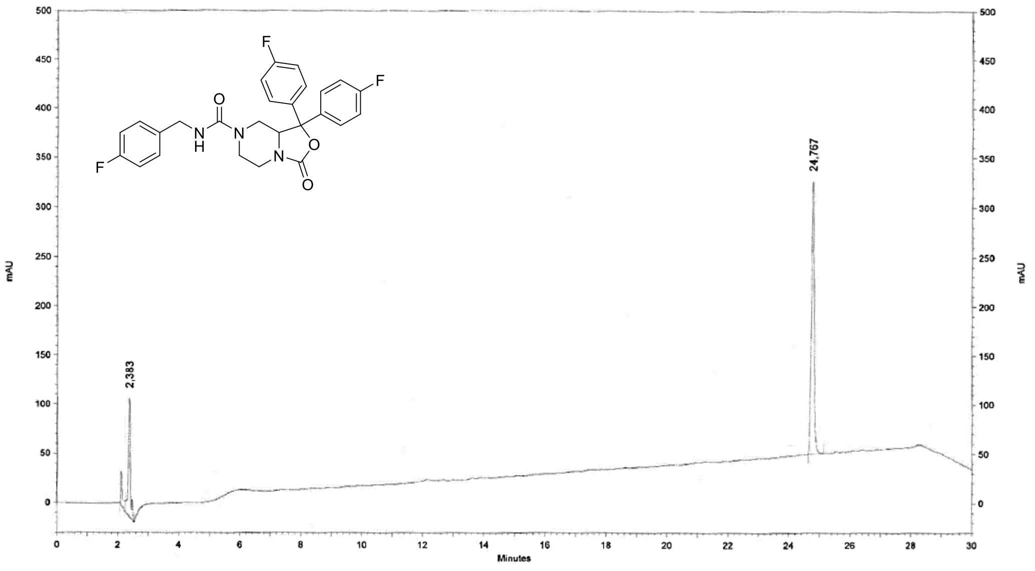

Compound 4

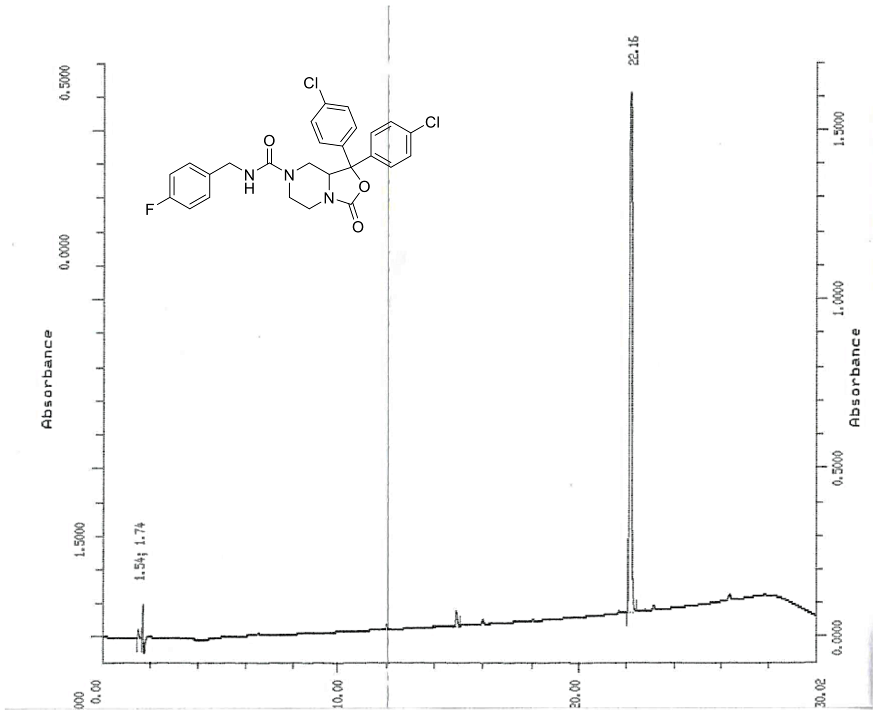

Compound 5

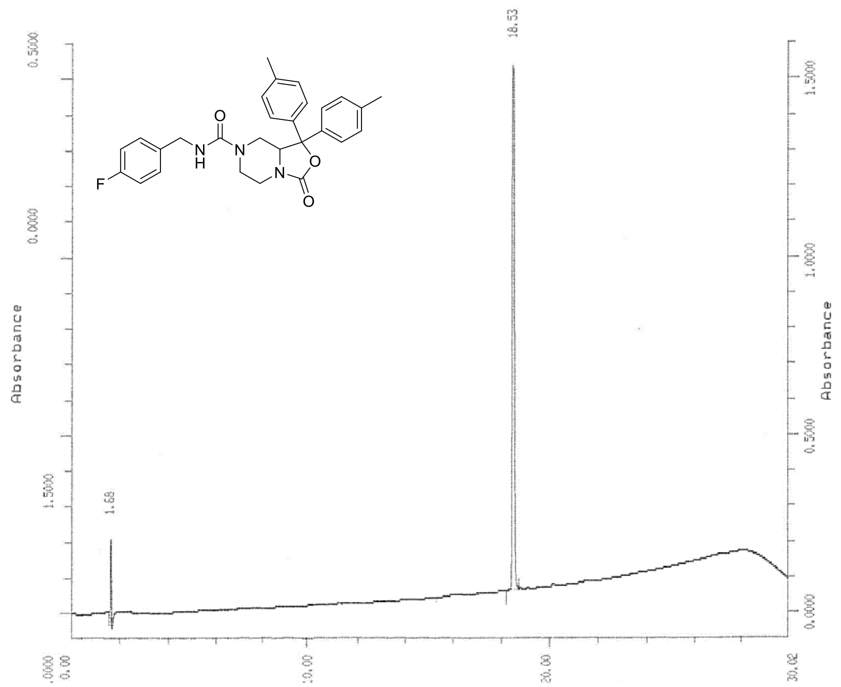

Compound 6

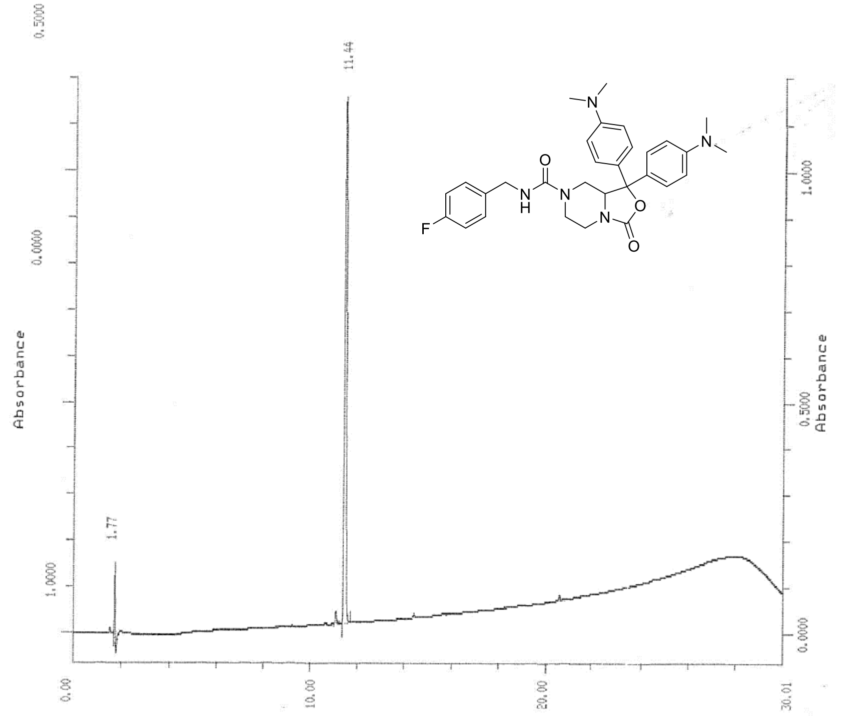

Compound 7

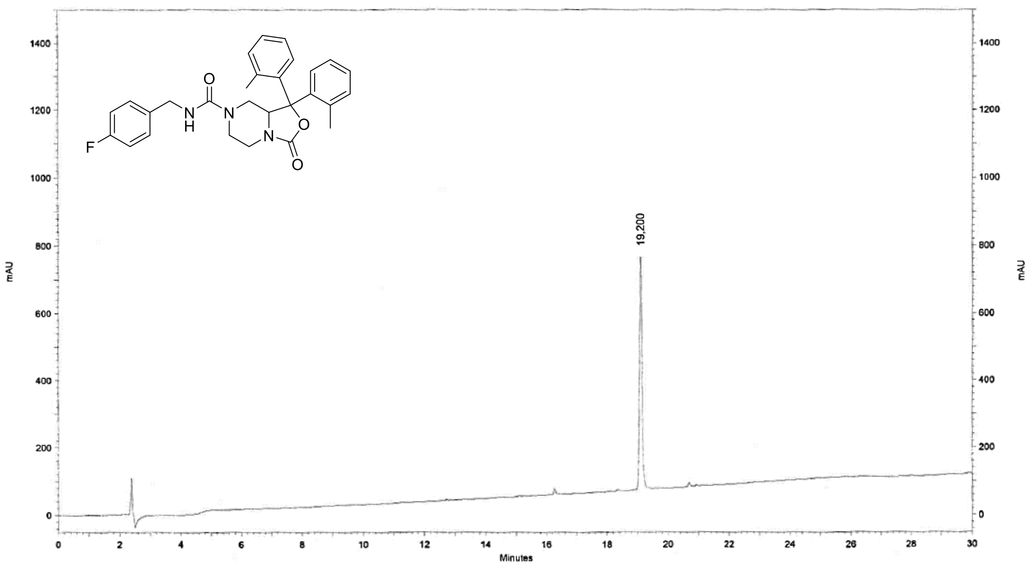

Compound 8

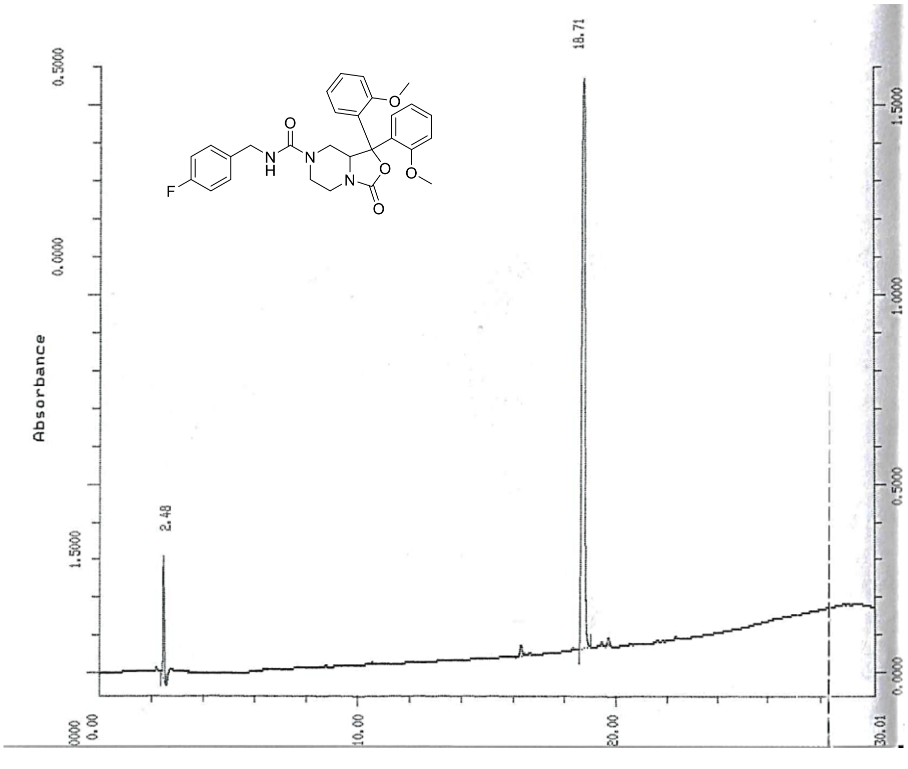

Compound 9

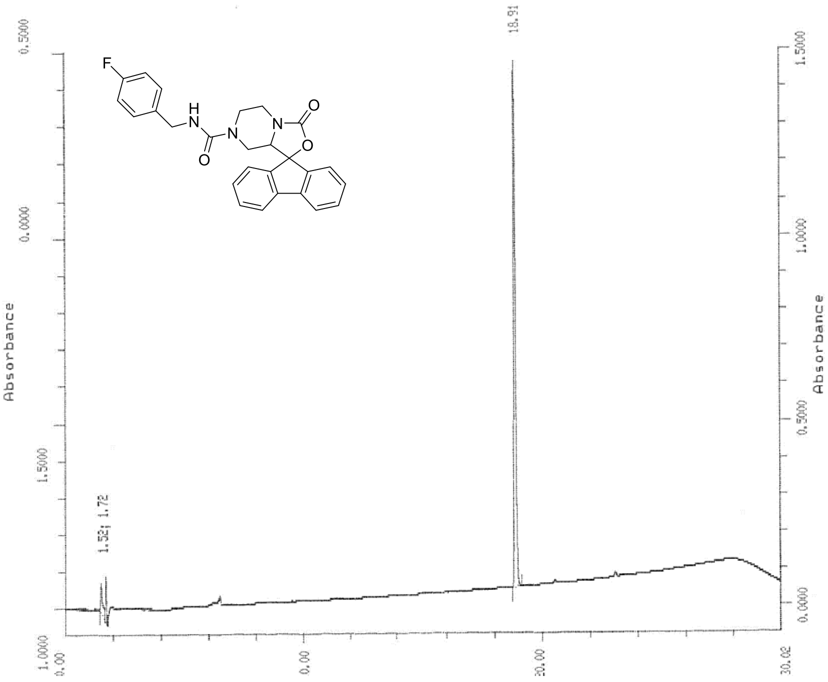

Compound 10

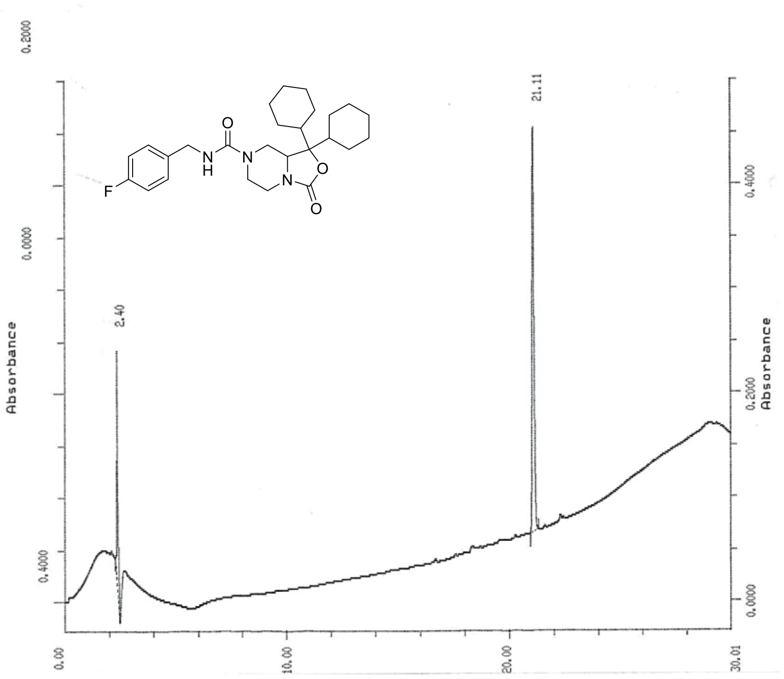

Compound 11

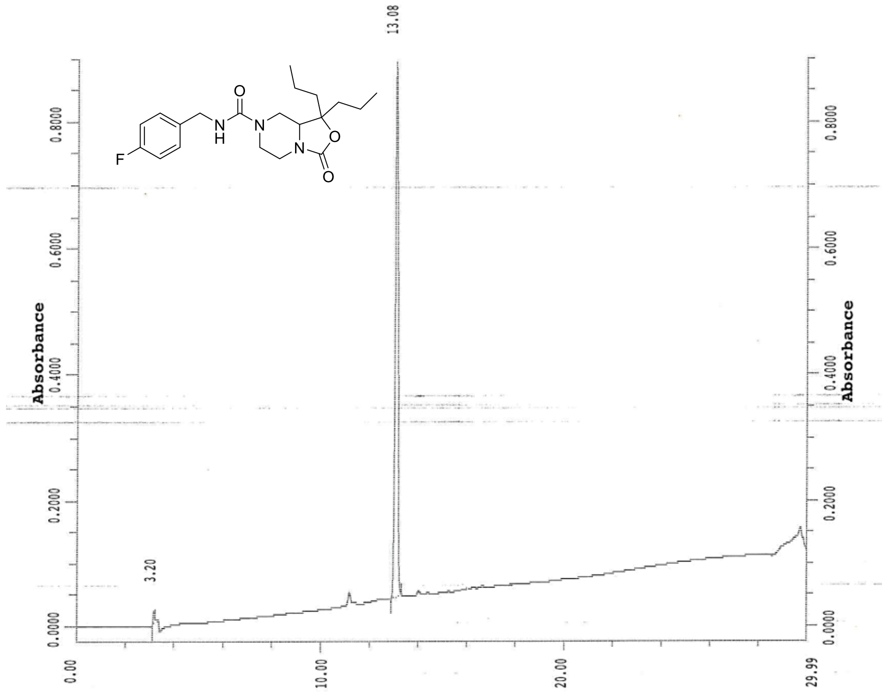

Compound 12

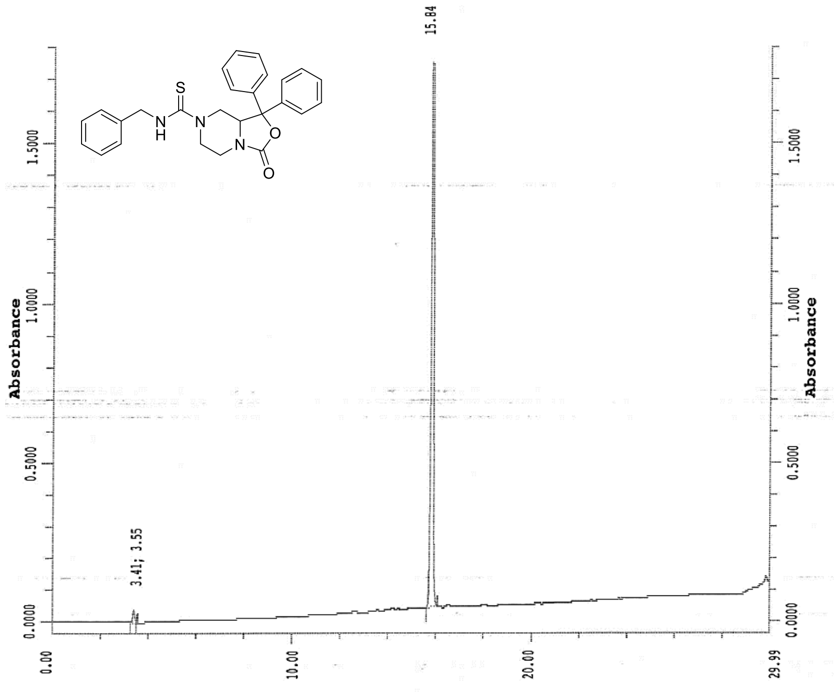

Compound 13

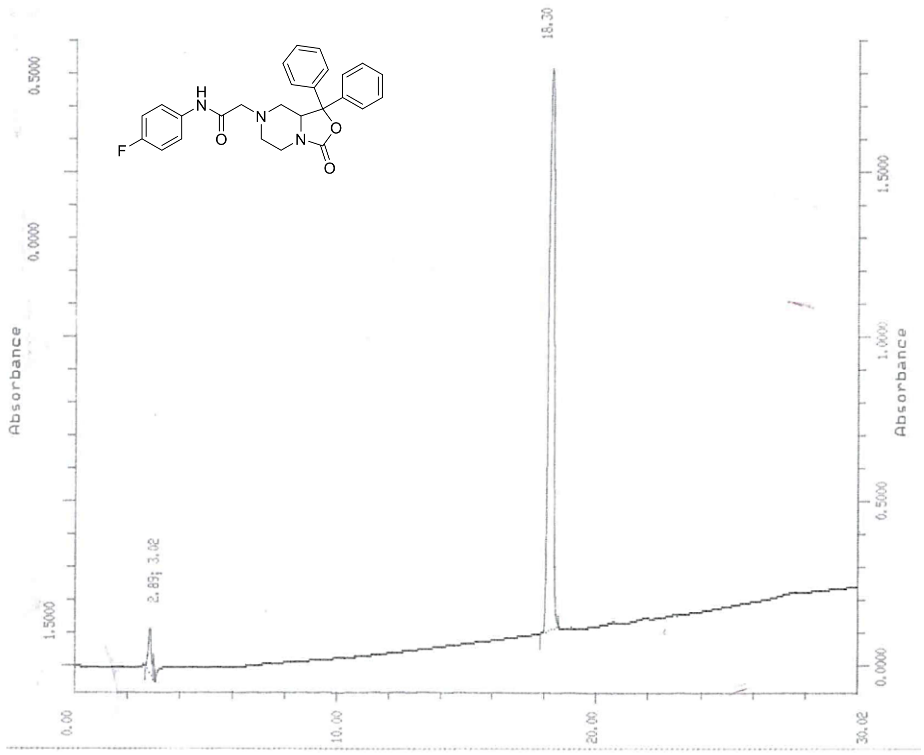

Compound 14

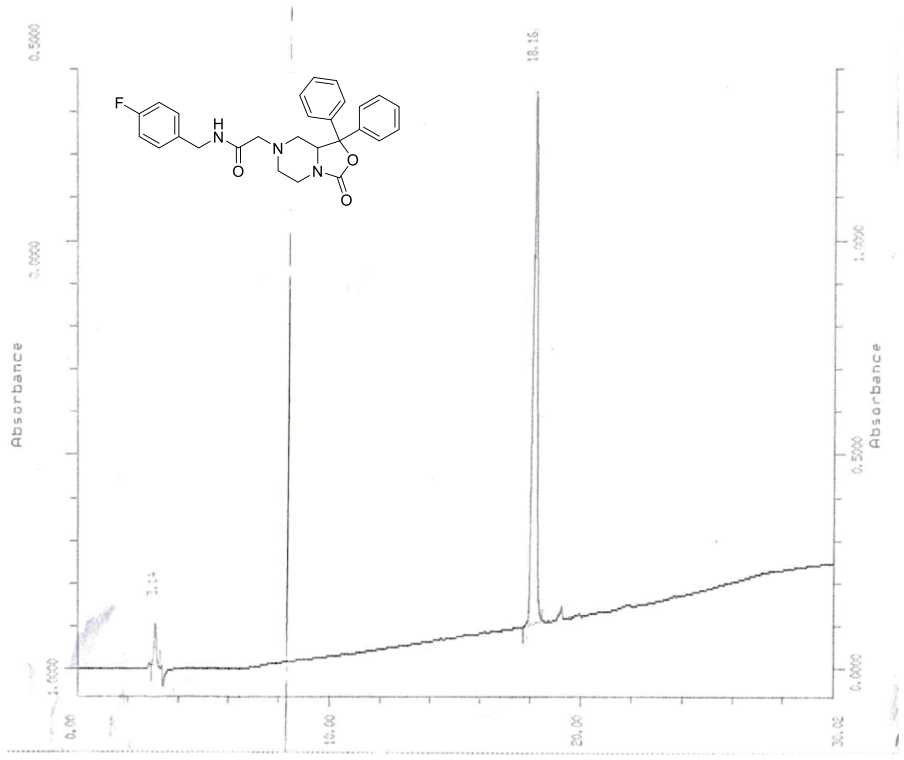

Compound 15

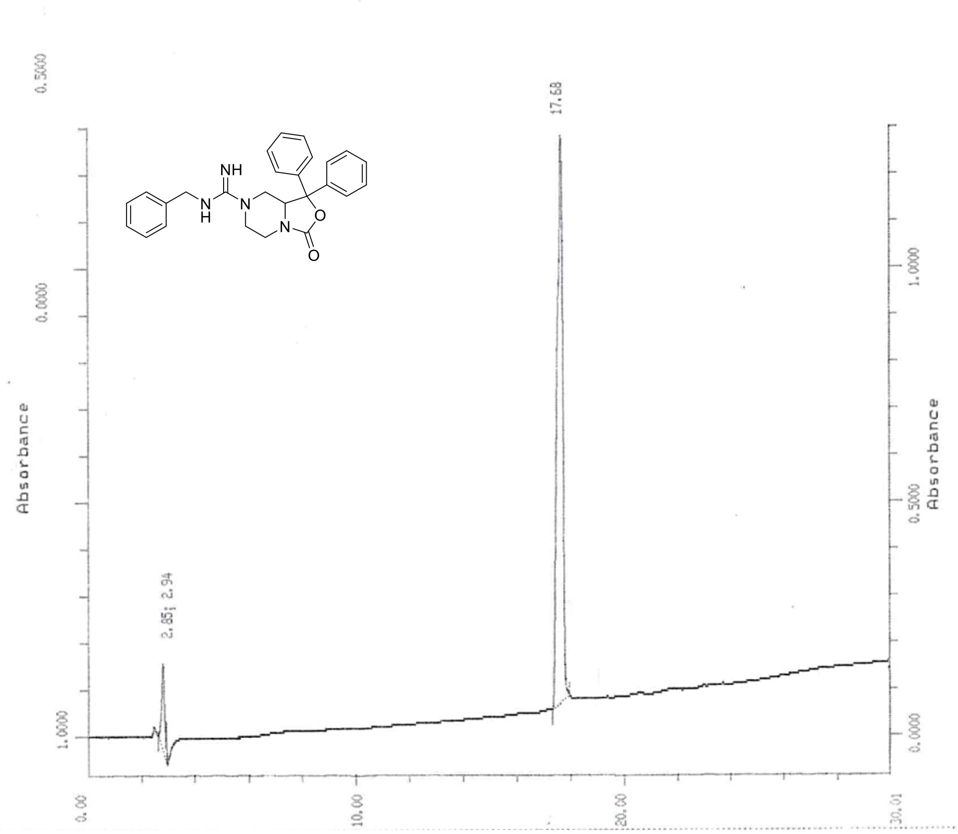

Compound 16

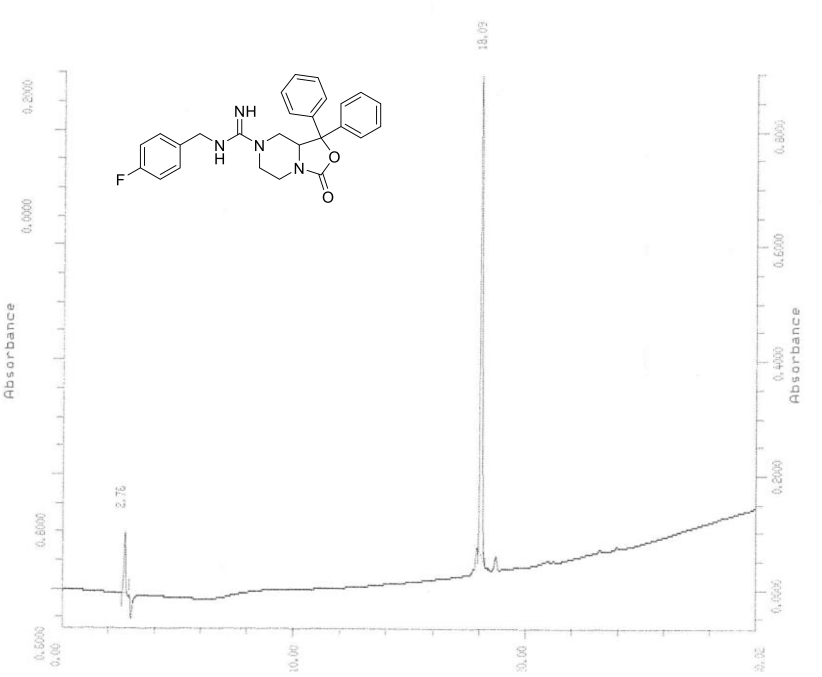

Compound 17

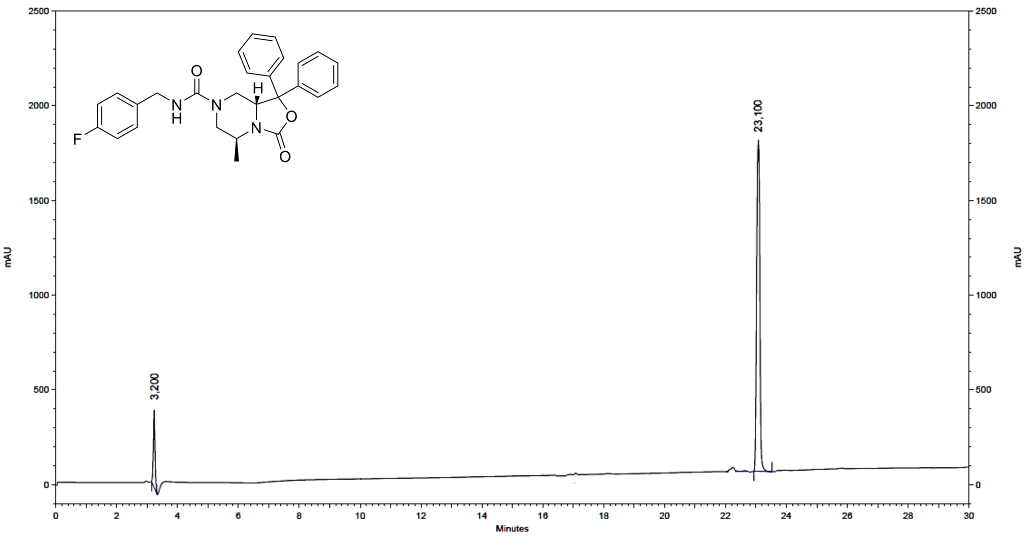

Compound 18

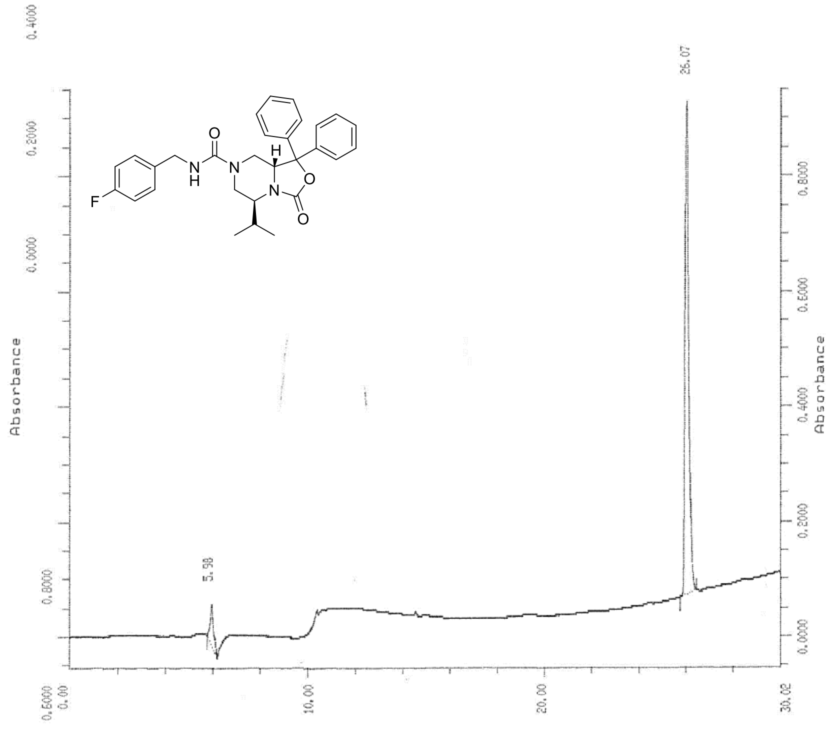

Compound 19

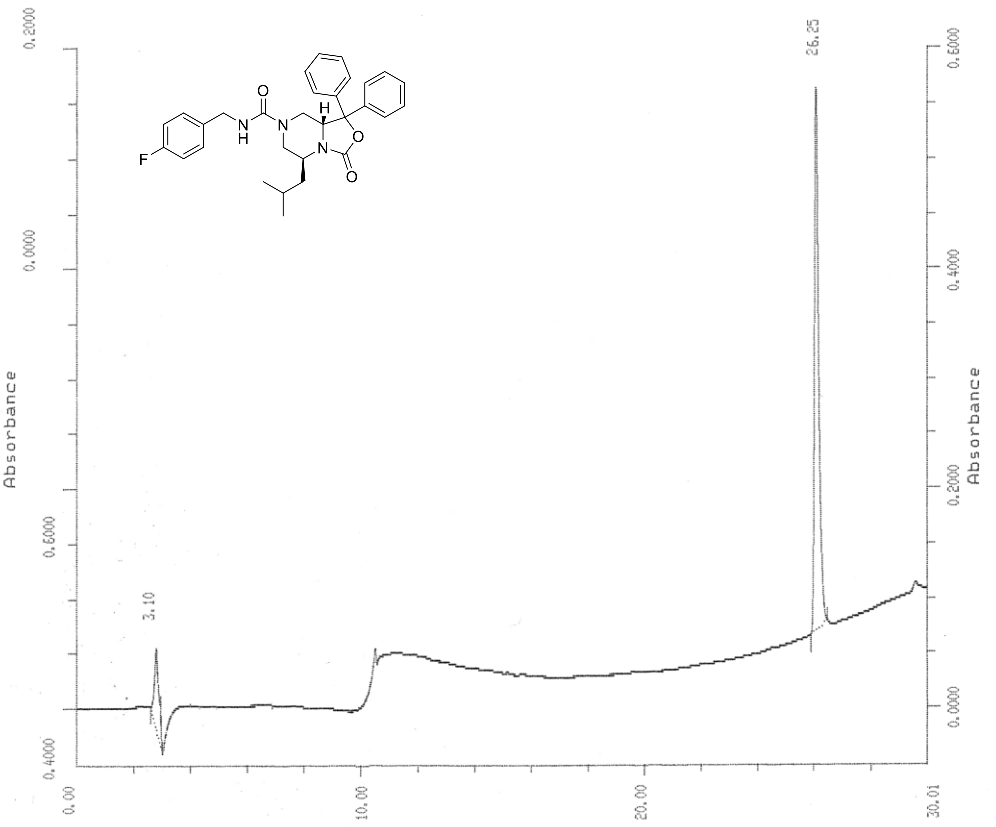

Compound 20

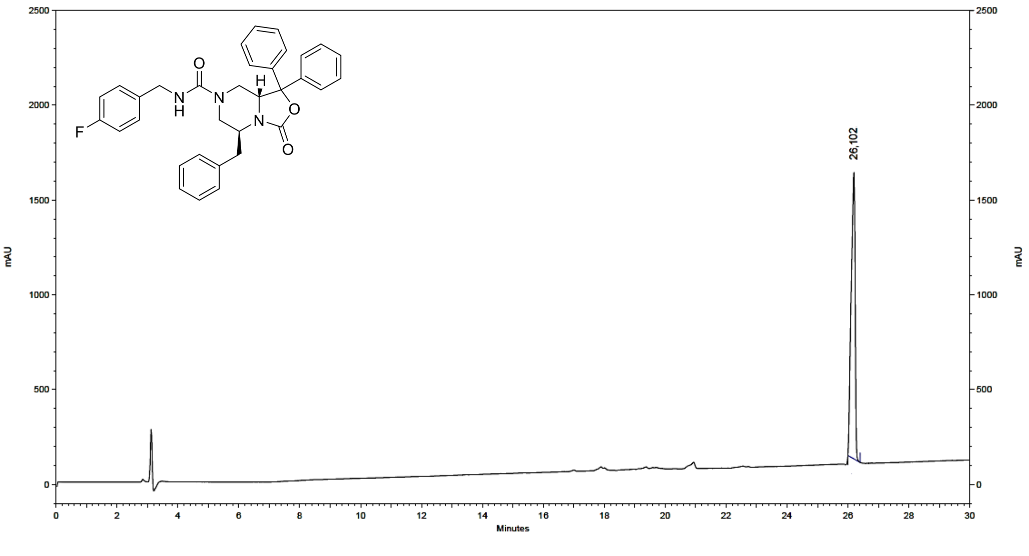

Compound 21

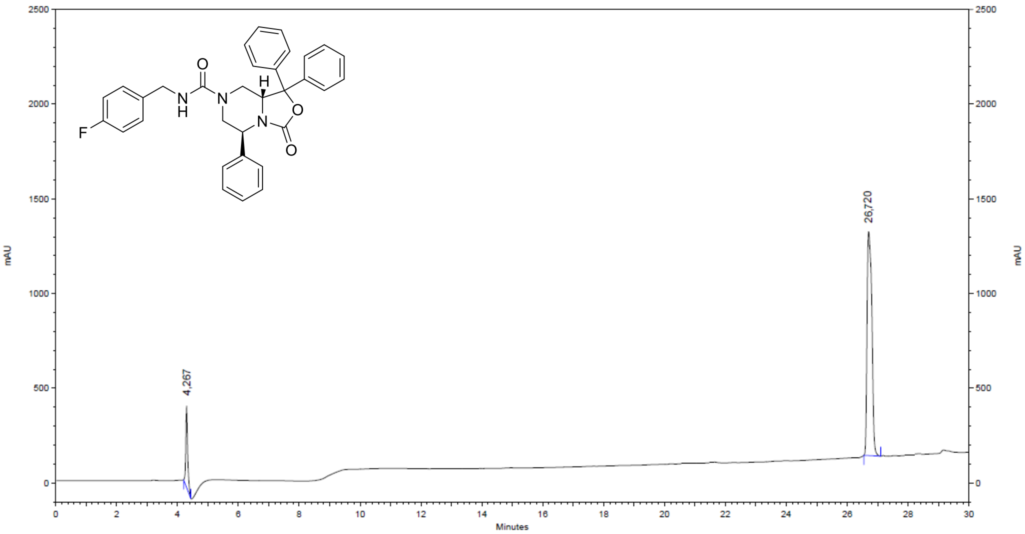

Supplement: Supplementary file 1 — jm0c02223_si_001.pdf [file jm0c02223_si_001.pdf]
